# Automated virtual reality cognitive therapy versus virtual reality mental relaxation therapy for the treatment of persistent persecutory delusions in patients with psychosis (THRIVE): a parallel-group, single-blind, randomised controlled trial in England with mediation analyses

**DOI:** 10.1016/S2215-0366(23)00257-2

**Published:** 2023-09-21

**Authors:** Daniel Freeman, Rachel Lister, Felicity Waite, Ushma Galal, Ly-Mee Yu, Sinéad Lambe, Ariane Beckley, Emily Bold, Lucy Jenner, Rowan Diamond, Miriam Kirkham, Eve Twivy, Chiara Causier, Lydia Carr, Simone Saidel, Rebecca Day, Alejandro Beacco, Aitor Rovira, Annabel Ivins, Ryan Nah, Mel Slater, David M Clark, Laina Rosebrock

**Affiliations:** Department of Experimental Psychology, University of Oxford, Oxford, UK; Oxford Health NHS Foundation Trust, Oxford, UK; Department of Experimental Psychology, University of Oxford, Oxford, UK; Black Country Healthcare NHS Foundation Trust, Dudley, UK; Department of Experimental Psychology, University of Oxford, Oxford, UK; Oxford Health NHS Foundation Trust, Oxford, UK; Oxford Primary Care Clinical Trials Unit, Nuffield Department of Primary Care Health Sciences, University of Oxford, Oxford, UK; Department of Experimental Psychology, University of Oxford, Oxford, UK; Oxford Health NHS Foundation Trust, Oxford, UK; Department of Experimental Psychology, University of Oxford, Oxford, UK; Department of Experimental Psychology, University of Oxford, Oxford, UK; Oxford Health NHS Foundation Trust, Oxford, UK; Department of Experimental Psychology, University of Oxford, Oxford, UK; Northamptonshire Healthcare NHS Foundation Trust, Kettering, UK; Event Lab, Faculty of Psychology Spain, University of Barcelona, Barcelona, Spain; Universitat Politècnica de Catalunya, Barcelona, Spain; Department of Experimental Psychology, University of Oxford, Oxford, UK; Oxford Health NHS Foundation Trust, Oxford, UK; Northamptonshire Healthcare NHS Foundation Trust, Kettering, UK; Northamptonshire Healthcare NHS Foundation Trust, Kettering, UK; Central and North West London NHS Foundation Trust, London, UK; Event Lab, Faculty of Psychology Spain, University of Barcelona, Barcelona, Spain; Institute of Neurosciences, University of Barcelona, Barcelona, Spain; Department of Experimental Psychology, University of Oxford, Oxford, UK; Oxford Health NHS Foundation Trust, Oxford, UK

## Abstract

**Background:**

Persecutory delusions are a major psychiatric problem that often do not respond sufficiently to standard pharmacological or psychological treatments. We developed a new brief automated virtual reality (VR) cognitive treatment that has the potential to be used easily in clinical services. We aimed to compare VR cognitive therapy with an alternative VR therapy (mental relaxation), with an emphasis on understanding potential mechanisms of action.

**Methods:**

THRIVE was a parallel-group, single-blind, randomised controlled trial across four UK National Health Service trusts in England. Participants were included if they were aged 16 years or older, had a persistent (at least 3 months) persecutory delusion held with at least 50% conviction, reported feeling threatened when outside with other people, and had a primary diagnosis from the referring clinical team of a non-affective psychotic disorder. We randomly assigned (1:1) patients to either THRIVE VR cognitive therapy or VR mental relaxation, using a permuted blocks algorithm with randomly varying block size, stratified by severity of delusion. Usual care continued for all participants. Each VR therapy was provided in four sessions over approximately 4 weeks, supported by an assistant psychologist or clinical psychologist. Trial assessors were masked to group allocation. Outcomes were assessed at 0, 2 (therapy mid-point), 4 (primary endpoint, end of treatment), 8, 16, and 24 weeks. The primary outcome was persecutory delusion conviction, assessed by the Psychotic Symptoms Rating Scale (PSYRATS; rated 0–100%). Outcome analyses were done in the intention-to-treat population. We assessed the treatment credibility and expectancy of the interventions and the two mechanisms (defence behaviours and safety beliefs) that the cognitive intervention was designed to target. This trial is prospectively registered with the ISRCTN registry, ISRCTN12497310.

**Findings:**

From Sept 21, 2018, to May 13, 2021 (with a pause due to COVID-19 pandemic restrictions from March 16, 2020, to Sept 14, 2020), we recruited 80 participants with persistent persecutory delusions (49 [61%] men, 31 [39%] women, with a mean age of 40 years [SD 13, range 18–73], 64 [80%] White, six [8%] Black, one [1%] Indian, three [4%] Pakistani, and six [8%] other race or ethnicity). We randomly assigned 39 (49%) participants assigned to VR cognitive therapy and 41 (51%) participants to VR mental relaxation. 33 (85%) participants who were assigned to VR cognitive therapy attended all four sessions, and 35 (85%) participants assigned to VR mental relaxation attended all four sessions. We found no significant differences between the two VR interventions in participant ratings of treatment credibility (adjusted mean difference –1·55 [95% CI –3·68 to 0·58]; p=0·15) and outcome expectancy (–0·91 [–3·42 to 1·61]; p=0·47). 77 (96%) participants provided follow-up data at the primary timepoint. Compared with VR mental relaxation, VR cognitive therapy did not lead to a greater improvement in persecutory delusions (adjusted mean difference –2·16 [–12·77 to 8·44]; p=0·69). Compared with VR mental relaxation, VR cognitive therapy did not lead to a greater reduction in use of defence behaviours (adjusted mean difference –0·71 [–4·21 to 2·79]; p=0·69) or a greater increase in belief in safety (–5·89 [–16·83 to 5·05]; p=0·29). There were 17 serious adverse events unrelated to the trial (ten events in seven participants in the VR cognitive therapy group and seven events in five participants in the VR mental relaxation group).

**Interpretation:**

The two VR interventions performed similarly, despite the fact that they had been designed to affect different mechanisms. Both interventions had high uptake rates and were associated with large improvements in persecutory delusions but it cannot be determined that the treatments accounted for the change. Immersive technologies hold promise for the treatment of severe mental health problems. However, their use will likely benefit from experimental research on the application of different therapeutic techniques and the effects on a range of potential mechanisms of action.

**Funding:**

Medical Research Council Developmental Pathway Funding Scheme and National Institute for Health and Care Research Oxford Health Biomedical Research Centre.

## Introduction

Persecutory delusions are characterised by an individual strongly but incorrectly believing that others are deliberately trying to harm them. Such delusions can lead to psychiatric hospital admission.^1^ In UK clinical services, half of patients diagnosed with non-affective psychosis still experience severe paranoia despite standard treatments.^2^ Effective and implementable interventions are needed for persecutory delusions. Here, we report the study of a brief automated virtual reality (VR) cognitive intervention for persecutory delusions compared with a credible alternative VR intervention. We examined mechanisms of action to enable understanding of how persecutory delusions might change with treatment.

We have argued that immersive VR—ie, interactive computer-generated environments—might provide a powerful means to study and treat psychosis. We did an experimental test of VR in the treatment of persecutory delusions.^3^ We randomly assigned 30 participants with persecutory delusions (despite taking antipsychotic medication) in the context of schizophrenia spectrum disorders to VR cognitive therapy or VR exposure therapy. Both treatments were informed by the mechanistic principle that by entering VR simulations of social situations participants would relearn that they are safe around other people to counteract paranoia. In VR cognitive treatment, participants were also encouraged to drop their within-situation defences (eg, avoiding eye contact, keeping their distance from people, and planning escape). Such defences normally prevent the processing of disconfirmatory evidence for an inaccurate threat belief because the absence of harm is attributed to the use of such behaviours (eg, “I would have been harmed if I hadn’t been alert”).^4^ In both conditions we used a single experimental session of 30 minutes in graded VR environments (train and lift scenarios) with psychological advice provided by a therapist. Before and after the VR session, participants completed a challenging real-world task (eg, going into a shop), and degree of belief in the delusion was assessed. Compared with VR exposure therapy, VR cognitive treatment led to large reductions in delusions and in distress in the real world. Subsequently, a randomised controlled trial in the Netherlands with 116 patients with psychosis selected for having at least mild paranoid ideation showed that 16 sessions with a CBT therapist using VR produced significant reductions in persecutory ideation compared with a waiting list control group.^5^ Reduction in defence behaviours mediated the treatment effect. This work is encouraging for the potential of immersive technologies for severe mental health conditions.

Our VR development has focused on automating the delivery of psychological therapy by including a virtual coach in the programmes.^6^ This approach is well received by patients.^7^ Automation of therapy means that a wide range of the mental health workforce can support the intervention, enabling greater provision of psychological therapy for patients. Compared with treatment as usual, our six-session automated gameChange VR intervention targeting anxious avoidance led to moderate reductions in paranoia for patients with psychosis who had the most severe difficulties.^8^ Automation of therapy will facilitate greater standardisation of psychological treatment and hence outcomes. Results from trials should translate to clinical practice. We therefore set out to assess an automated version of VR cognitive treatment for persecutory delusions (Therapeutic Realistic Immersive Virtual Environments [THRIVE]), with the emphasis on understanding the potential mechanisms of action. We aimed to investigate the mediators of VR cognitive treatment that may explain delusion change beyond the non-specific benefits of intervention such as time with a mental health professional or in VR. Meta-analyses indicate that better treatment outcomes are predicted by the patient’s positive initial views of the credibility and potential benefits (ie, expectancy) of a therapy.^9,10^ Therefore, we aimed to test VR cognitive treatment against a credible alternative VR therapy for persecutory delusions that might have benefits but potentially via different mechanisms. Given the close connection between anxiety and persecutory delusions—paranoia probably builds on anxious threat cognitions^11,12^—we chose VR mental relaxation as the comparison condition. Relaxation can reduce anxiety^13^ and, therefore, might plausibly reduce paranoia. We included both mindfulness^14^ and progressive muscle relaxation^15^ exercises in various calming VR environments. Meta-analysis indicates that acceptance-based and mindfulness-based interventions might have small effects in reducing positive psychotic symptoms.^16^ Evidence suggests that VR relaxation is feasible, acceptable, and might reduce stress.^17–19^ In a randomised controlled trial with 81 patients with psychosis, social cognition training in VR was tested against VR relaxation.^20^ Participants were selected on the basis of social cognition difficulties and not the presence of current psychotic experiences. The VR interventions were provided within 16 twice-weekly sessions with a psychologist. The study found no significant outcome differences in paranoia (or in other psychiatric symptoms) between the two interventions. There was little evidence that VR social cognition training or VR relaxation reduced persecutory ideation or positive symptoms.

Our primary hypothesis was that, compared with VR mental relaxation, automated VR cognitive therapy would lead to a greater reduction in degree of conviction in the persecutory delusion at the end of treatment. The secondary hypotheses were that, compared with VR mental relaxation, VR cognitive treatment would lead to reductions in distress in real-world situations, overall paranoia, delusion severity, and suicidal ideation, and increases in activity, psychological wellbeing, and quality of life at the end of treatment. We also hypothesised that treatment effects would be maintained at follow-up. Mediation was built into the trial design to test how the VR therapy might work. We hypothesised that change in delusion conviction via VR cognitive therapy would be mediated by changes in beliefs about safety and use of defence behaviours.

## Methods

### Study design and participants

THRIVE was a parallel-group, single-blind, randomised controlled trial across four National Health Service (NHS) trusts in England. Research assistants sought referrals from four NHS mental health trusts: Oxford Health NHS Foundation Trust, Berkshire Healthcare NHS Foundation Trust, Northamptonshire Healthcare NHS Foundation Trust, and Milton Keynes locality of Central and North West London NHS Foundation Trust.

Eligible participants were individuals aged 16 years or older with persistent (≥3 months) persecutory delusions (as defined by Freeman and Garety^21^) held with at least 50% conviction, who reported feeling threatened when outside with other people, and who had been given a clinical diagnosis of non-affective psychosis. Exclusion criteria were a primary diagnosis of alcohol or substance use disorder; photosensitive epilepsy; significant visual, auditory, or balance impairment; current receipt of another psychological therapy; insufficient comprehension of English; in forensic settings; organic syndrome; significant learning disability; or current active significant suicidal intent and plan. Written informed consent was obtained before participation.

The trial received approval from an NHS Research Ethics Committee (NHS South Central-Oxford B Research Ethics Committee, 18/SC/0316); a notice of no objection from the UK’s Medicines and Healthcare products Regulatory Agency (CI/2018/0041) for a trial of a medical device; was registered prospectively (ISRCTN12497310); and the protocol was published.^22^

### Randomisation and masking

We randomly assigned participants (1:1) to VR cognitive therapy plus usual care or VR mental relaxation plus usual care. Randomisation was done by the trial co-ordinator using a validated online system. Randomisation was done using a permuted blocks algorithm, with randomly varying block size, stratified by severity of delusion (moderate [50–75%] conviction or high [≥76%] conviction).

Trial assessors were masked to group allocation. If group allocation was revealed, another masked assessor replaced the unmasked assessor. Assessors were unmasked on six occasions and five of these assessments were successfully re-masked (one 2-week assessment was done unmasked).

### Procedures

Both types of VR therapy were delivered by either a clinical psychologist or assistant psychologist. Each was provided over four sessions of 30 min in VR over 4 weeks. The staff member was present throughout. The hardware used was an HTC Vive Pro headset (HTC, New Taipei City, Taiwan) and a Dell G5 15 5590 laptop (Dell Technologies, Round Rock, TX, USA). Assessments were done at 0, 2, 4 (end of treatment), 8, 16, and 24 weeks after randomisation.

The treatment aim for VR cognitive therapy was for participants to test their fear expectations around other people in order to relearn safety. VR cognitive treatment comprised repeated behavioural experiment tests to help participants learn that they are safer than they had thought. The VR treatment was set in a (virtual) shopping centre. A virtual coach guided the person through the treatment, including encouraging the dropping of defence behaviours and eliciting feedback to tailor the progression of the treatment. At the beginning of the first session, the virtual coach, in a virtual office in the shopping centre, explained the rationale behind the treatment. The participant then selected one of four VR scenarios in which to begin. The scenarios were a lift, a central atrium area, a cafe, and a clothes shop. Each offered five levels of difficulty (eg, the number and proximity of people in the social situation increased) and participants worked their way through each level. The participant could choose a different scenario in each session or repeat a previous scenario. The participant could walk around the scenarios. Throughout the sessions, a participant’s responses to questions from the virtual coach were given by means of a pop-up screen from a virtual watch on the participant’s virtual arm. Safety-belief ratings were repeated within VR at the beginning and end of each treatment session. To clarify the purpose of the treatment for participants the procedure was described as VR confidence building because we wanted people to feel confident being in everyday situations around other people. The staff member set up the VR equipment, responded to questions, and at the end of the session helped the participant plan real-world tests to conduct between sessions. The THRIVE VR cognitive therapy was a different programme to gameChange.^6^

In VR mental relaxation, the staff member explained to participants that the way to deal with a fear about other people is to be calm in your own mind. Participants were told that VR mental relaxation achieves this relaxation by helping switch off our alarm systems, resulting in reduced anxiety and creating a sense of safety. Participants were told that VR mental relaxation would help participants improve at using mental relaxation techniques when they felt anxious. In each session, participants chose from a selection of calm VR environments (eg, beach, forest, or lake) in which to practise these techniques. The environments were non-social. The environments, but not the relaxation exercises, were taken from a commercially available VR relaxation programme (Guided Meditation VR, Cubicle Ninjas, Glen Ellyn, IL, USA). Patients completed two relaxation exercises per session, each lasting approximately 10 min. These exercises were progressive muscle relaxation, mindfully allowing thoughts to float by, mindfully shifting attention from negative thoughts to the external environment, coloured breathing, soothing breathing and letting worries float by, and soothing rhythmic breathing.^14,15,23,24^ The exercises were played via an audio file recorded by our team. Participants were encouraged by the staff member to select a different VR environment for each exercise. Exercises were completed while seated and participants were offered a break between exercises. At the end of the session, participants were encouraged to reflect on which exercises they found useful and schedule time each day to practise the exercises, both at home and while outside in anxiety-provoking situations. The staff member was responsible for providing the rationale for the therapy, setting up the VR equipment, helping participants choose the environments, playing the audio files, reviewing the exercises with participants, and assisting with homework setting (including making a check-in telephone call or text during the week).

At the beginning and end of the first VR session for each treatment, participants were asked to complete the Simulator Sickness Questionnaire.^25^ We used a simple raw total score. The total score ranged between 0 and 48, with higher scores indicating greater discomfort.

Usual care, and other relevant health economic data, were recorded using an adapted version of the Economic Patient Questionnaire,^26^ and usually comprised prescription of antipsychotic medications, visits from a community mental health worker, and occasional outpatient appointments with a psychiatrist.

### Outcomes

The primary outcome was degree of conviction in the persecutory delusion assessed in the Psychotic Symptoms Rating Scale–Delusions (PSYRATS)^27^ interview using a visual analogue scale (VAS; 0% [do not believe it] to 100% [absolutely believe it]). Secondary outcomes were a real-world behavioural test assessing avoidance and distress using the Oxford-Behavioural Avoidance Task (O-BAT),^3^ activity levels assessed using actigraphy (over 7 days) and a time-budget for meaningful activity,^28^ quality of life using the EQ-5D-5L,^29^ suicidal ideation measured by the Columbia Suicide Severity Rating Scale,^30^ overall paranoia assessed by the Revised-Green et al Paranoid Thoughts Scale (R-GPTS),^31^ delusion severity using the PSYRATS interview,^27^ and psychological wellbeing using the Warwick-Edinburgh Mental Wellbeing Scale^32^ and the Questionnaire about the Process of Recovery.^33^ For mediation, we assessed the use of defence behaviours using the interviewer-rated Safety Behaviours Questionnaire–Persecutory delusions (SBQ)^34^ and strength of safety beliefs using a self-report 0–100% VAS.^35^ The main delusion measures (conviction rating, PSYRATS, and R-GPTS scores) and mediation measures (SBQ and strength of safety beliefs scores) were completed at all assessment timepoints (0, 2, 4, 8, 16, and 24 weeks), and the full assessment battery was completed at 0, 4, and 24 weeks. The O-BAT was completed only at 0 and 4 weeks. The credibility and expectancy of the treatments was assessed with the Credibility/Expectancy Questionnaire^36^ at the beginning of the first treatment session after the treatment rationale was provided. Original GPTS scores were analysed post hoc.

At the end of trial participation, we checked medical notes for adverse events. An independent data monitoring and ethics committee (DMEC) chair rated whether any serious adverse event was related to treatment or trial procedures. A report was written by the trial team and a clinical judgement was made, concerning the type of event, timing, and what was known about what may have led to the event.

### Choice of primary outcome

The primary outcome measure was degree of conviction in the persecutory delusion, measured using PSYRATS. We used a dimensional score from the VAS (0–100%), as in the pilot study^3^ and Feeling Safe Trial.^35^ This measure was chosen as the primary outcome since the intention in the interventions was to reduce how much the delusional beliefs are believed, a key criterion for the presence of a delusion. PSYRATS is a brief, easy-to-use assessment that requires no permissions to use. The minimum clinically important difference on this scale is unknown.

### Statistical analysis

We aimed to enrol 90 patients into the trial, with 45 in each group. This sample size took into consideration an attrition rate of 15% and would provide approximately 90% power to detect a statistically significant treatment effect at 4 weeks, using an independent groups *t* test and a significance level of 0·05, if the true standardised effect size was 0·75, reflecting a 15-point reduction in delusional conviction assuming an SD of 20 as in the pilot study.^3^ Further power is likely to be gained by use of mixed-effects models.

There was a planned blinded interim analysis for the 4-week data for the first 30 patients, providing simple descriptive statistics and an initial estimate of the 95% CI for the treatment effect. This interim analysis provided an estimate of conditional power (ie, power given the data obtained so far).^37,38^ We planned to stop the trial if the interim estimate of effect size, d, was 0·1 or lower, implying that the conditional power of a full trial, on the basis of the interim results and the hypothesised effect size of 0·75, would be 60% or lower. The interim analysis was shared by the trial statisticians with the independent DMEC members and the main trial team were simply informed to continue the trial.

The full statistical analysis plan and report are provided in the appendix (pp 35–124). The primary analysis included all participants for whom data were available and according to the group to which participants were randomly assigned. We used Stata (version 16.1) for all analyses.

Analysis of the primary outcome was done using linear mixed-effects regression, which facilitated modelling of the response at all timepoints simultaneously. We fitted the baseline outcome measure score, the severity of delusion stratification variable, time, and treatment assignment as fixed effects with a patient-specific random intercept. We also fitted an interaction between time and randomised group as a fixed effect to allow estimation of treatment effect at each timepoint. We used a similar approach for the secondary outcome analysis. We used p<0·05 as the level of significance for all tests. Results are reported as mean differences between treatment groups, with 95% CIs. Treatment differences estimated from the linear mixed-effects models were additionally reported for the primary outcome as standardised mean differences (mean group difference divided by whole group SD at baseline).

We used linear mixed-effects regression models to test for mediation of VR therapy effects on the outcome through the putative mediators. Analyses were adjusted for baseline measures of the mediator, outcomes, and possible measured confounders. We included repeated measurement of mediators and outcomes to account for classical measurement error and baseline confounding. Mediation was estimated separately using the values of the mid-treatment mediators to examine temporal precedence of changes and the end-of-treatment values of the mediators to examine contemporaneous changes.

### Changes to the protocol

Recruitment had to be suspended because of the first COVID-19 lockdown in March, 2020. At this time collection of O-BAT data (the real-world behavioural task) had to be suspended. The measure was removed from the assessment battery in July, 2020, because it involved face-to-face visits with a researcher to many locations that were now closed. Actigraphy data collection was largely suspended during this time too. The possibility for remote administration of self-report measures was added in March, 2020. There were two post hoc analyses to aid in interpretation of the results. We tested whether treatment credibility and expectancy scores predicted patient outcomes across the two VR interventions. We also tested whether change in the hypothesised mechanisms of action explained change in delusions across the two VR interventions.

### Role of the funding source

The funders of the study had no role in study design, data collection, data analysis, data interpretation, or writing of the report.

## Results

Recruitment took place from Sept 21, 2018, to May 13, 2021. Because of the COVID-19 pandemic, recruitment was suspended on March 16, 2020, and began again from Sept 14, 2020. Final follow-up data were collected on Oct 28, 2021. We assessed 194 patients for eligibility, of whom 80 (41%) were enrolled and randomly assigned to the VR cognitive treatment therapy plus usual care group (n=39 [49%]) or the VR mental relaxation plus usual care group (n=41 [51%]; figure). 49 (61%) participants were men, 31 (39%) were women, with a mean age 40 years (SD 13; range 18–73), and 64 (80%) were White, six (8%) were Black, one (1%) was Indian, three (4%) were Pakistani, and six (8%) were of other race or ethnicity (table 1).

Uptake of the VR therapies was high. The mean number of sessions attended was 3·7 (SD 0·72, median 4) for VR cognitive treatment and 3·6 (SD 1·0, median 4) for VR mental relaxation. The total number of sessions attended for VR cognitive treatment was one (n=1 [3%]), two (n=3 [8%]), three (n=2 [5%]), and four (n=33 [85%]). The total number of sessions attended for VR mental relaxation was zero (n=2 [5%]), two (n=3 [7%]), three (n=1 [2%]), and four (n=35 [85%]). Five (13%) patients had VR cognitive treatment sessions at home and 34 (87%) in clinic settings. Ten (24%) patients had VR mental relaxation sessions at home and 29 (71%) in clinic settings. Three patients who were allocated to VR cognitive treatment and one patient allocated to VR mental relaxation had a reduced number of sessions because of the first COVID-19 lockdown since face-to-face appointments were not allowed. 73 (91%) patients provided Simulator Sickness Questionnaire (SSQ) data. The mean SSQ score in the VR cognitive treatment group was 7·3 (SD=5·4; n=36 [92%]) before entering VR in the first session and 5·9 (SD 4·5) at the end of the session, which was a significant reduction (mean reduction 1·43 [95% CI 0·03–2·84]; p=0·048). The mean SSQ score in the VR mental relaxation group was 7·6 (SD 6·3; n=37 [90%]) before entering VR in the first session and 5·9 (SD 5·5) at the end of the session, which was a significant reduction (mean reduction 1·72 [0·54–2·91]; p=0·0056). Controlling for initial SSQ score, the SSQ score did not differ between the groups after being in VR (B –0·17 [SE 0·77; p=0·83).

The mean degree of conviction in the persecutory delusion reduced from 78·1% (SD 18·1; n=39 [100%]) to 52·1% (SD 23·8; n=38 [97%]) at the end of treatment (4 weeks) in the VR cognitive treatment group (Cohen’s d=1·6) and reduced from 79·0% (SD 15·5; n=41 [100%]) to 56·6% (SD 25·0; n=39 [95%]) at the end of treatment in the VR mental relaxation group (Cohen’s d=1·3). We found no statistically significant difference between the two groups in delusional conviction at the end of treatment (table 2). We found no statistically significant differences between the two groups at any timepoint in the primary outcome (table 2). A similar pattern was found for the secondary measures, with most showing improvements in both VR treatment groups, with no statistically significant group differences on any of the measures at any of the timepoints (table 2). In a post hoc analysis we found no moderation of treatment effects by gender for the primary outcome at 4 weeks (appendix p 124). Using the original GPTS Part B to allow for comparisons with previous trials, persecutory ideation reduced in the VR cognitive treatment group from 55·5 (SD 12·7; n=39 [100%]) at baseline to 42·8 (SD 15·9; n=37 [95%]) at the end of treatment and 40·6 (SD 20·9; n=34 [87%]) at 24 weeks. In the VR mental relaxation group, persecutory ideation reduced from 51·7 (SD 13·7; n=41 [100%]) at baseline to 42·5 (SD 16·2; n=39 [95%]) at the end of treatment, and 41·2 (SD 18·1; n=37 [90%]) at 24 weeks. Using the original GPTS Part A, social reference ideation reduced in the VR cognitive treatment group from 48·3 (SD 15·0; n=39 [100%]) at baseline to 39·4 (SD 14·5; n=37 [95%]) at the end of treatment and 36·9 (SD 17·2; n=34 [87%]) at 24 weeks. In the VR mental relaxation group, social reference ideation reduced from 48·5 (SD 13·9; n=41 [100%]) at baseline to 40·1 (SD 14·4; n=39 [95%]) at the end of treatment, and 39·7 (SD 16·3; n=37 [90%]) at 24 weeks.

We found no evidence that either use of defence behaviours or strength of safety belief were differentially altered by one of the two treatments (path A; table 3). We also found no evidence of mediation (indirect effect; table 3). Across the whole group from baseline to 4 weeks, the use of defence behaviours reduced (adjusted mean difference –6·17 [95% CI –8·34 to –4·01; p<0·0001; n=70 [88%]) and safety beliefs increased (17·96 [95% CI 10·94 to 24·98; p<0·0001; n=75 [94%]; appendix p 106).

We found no significant differences between the two VR interventions in patient ratings of treatment credibility (adjusted mean difference –1·55 [95% CI –3·68 to 0·58; p=0·15) and outcome expectancy (–0·91 [–3·42 to 1·61; p=0·47). Across the whole group we found no association between baseline level of the persecutory delusion and ratings of treatment credibility (*r*=–0·02; p=0·84; n=76 [95%]) or expectancy (*r*=–0·14; p=0·228; n=76 [95%]; appendix p 105). Across the whole group higher ratings of treatment credibility (*r*=–0·28; p=0·015; n=74 [93%]) and higher levels of treatment expectancy (*r*=–0·28; p=0·015; n=74 [93%]) were significantly associated with a lower degree of conviction in the delusion at 4 weeks (end of treatment; appendix p 105).

Usual care was similar across the groups during the trial (table 4). No serious adverse event was judged as related to the VR therapies or trial procedures (table 5). The serious adverse events that occurred were primarily psychiatric (n=4) or acute (n=12) hospital admissions. There was one death, which was due to long-standing physical health difficulties.

## Discussion

We tested two different VR interventions with patients diagnosed with psychosis who had long-standing persecutory delusions. The trial had a high follow-up rate and the outcome assessments were done by assessors masked to group allocation. Most patients attended all the VR sessions that were offered. We found no evidence of the VR interventions causing serious adverse effects or simulator sickness. This evidence of adherence to VR and the infrequent occurrence of adverse effects is consistent with the gameChange trial.^7^ However, contrary to expectation, the two VR interventions had no difference in efficacy at any timepoint. This finding is in contrast with tests, for example, of cognitive approaches against exposure and relaxation in the treatment of anxiety disorders.^39^ Both VR groups showed large improvements over time but we found no significant differences on any primary or secondary outcome measure. Both interventions might have been effective; however, the absence of a third group, the primary limitation in the trial design, to control for the influence of time makes this conclusion uncertain. If both interventions are effective, then this finding could enable patient choice in treatment provision. Future research could also test the combination of the interventions.

There was a general broad commonality between the two VR therapies in how paranoia might be affected. At the beginning of the trial, we considered that both VR therapies could plausibly treat severe paranoia via anxiety reduction, specifically by lessening the influence of threat cognitions. However, the content of the VR in each therapy and the techniques taught were markedly different and we expected differences in the effects on specific cognitive processes. We hypothesised that the VR cognitive therapy would be significantly more effective than VR mental relaxation. In VR cognitive treatment, patients were exposed to everyday social situations in which paranoia is triggered and were encouraged to drop defence behaviours in order to relearn safety. The use of defence behaviours and strength of safety beliefs were accordingly assessed repeatedly during the trial. In VR mental relaxation, patients entered non-social, non-threatening situations, and were taught how to relax and create psychological distance from distressing thoughts. That we found no differences between the two interventions in effects on the proposed mechanisms of action was surprising. We found substantial reductions in the use of defence behaviours and large increases in safety beliefs in both conditions, although there was little evidence that these accounted for the changes in the delusions. Perhaps these findings are the result of placebo effects. The perceived credibility and expectancy of the therapies explained a small proportion of change in the persecutory delusions. However, our previous investigation of psychological treatment for persecutory delusions has shown that theoretically driven intervention can produce significantly greater change than equally credible but generic therapy.^35^ The cognitive intervention might also have been too brief. If the treatments were extended in length, then advantages for the VR cognitive treatment might emerge. This advantage has been seen in a randomised controlled trial of assisted online therapy for post-traumatic stress disorder, in which a cognitive-therapy approach was similar in efficacy at the mid-point of treatment (6 weeks) to that of stress management, but over the following 6 weeks of intervention cognitive therapy outperformed stress management.^40^

Another limitation in the trial was the low participant sample size, which means that potential smaller differences between the conditions could not be detected.

Important changes to the hardware have occurred since the trial began, so that the delivery model would now be different. Affordable untethered headsets can now be left with patients, allowing greater treatment doses at times that are most convenient for patients, which might affect efficacy.

In this trial of automated VR cognitive therapy versus VR mental relaxation, both had similar beneficial effects on persecutory delusions, use of defence behaviours, and belief in safety, although the absence of a control group means that efficacy cannot be confirmed. Further mechanistic research and treatment development is needed. A programme of experimental research testing the effects of implementation within VR of different therapeutic techniques on a wide mechanistic battery could substantially increase learning about the techniques and how best to apply them. This research could form the basis for the development of more efficacious VR treatment.

## Supplementary Material

Supplementary appendix

## Figures and Tables

**Figure:**
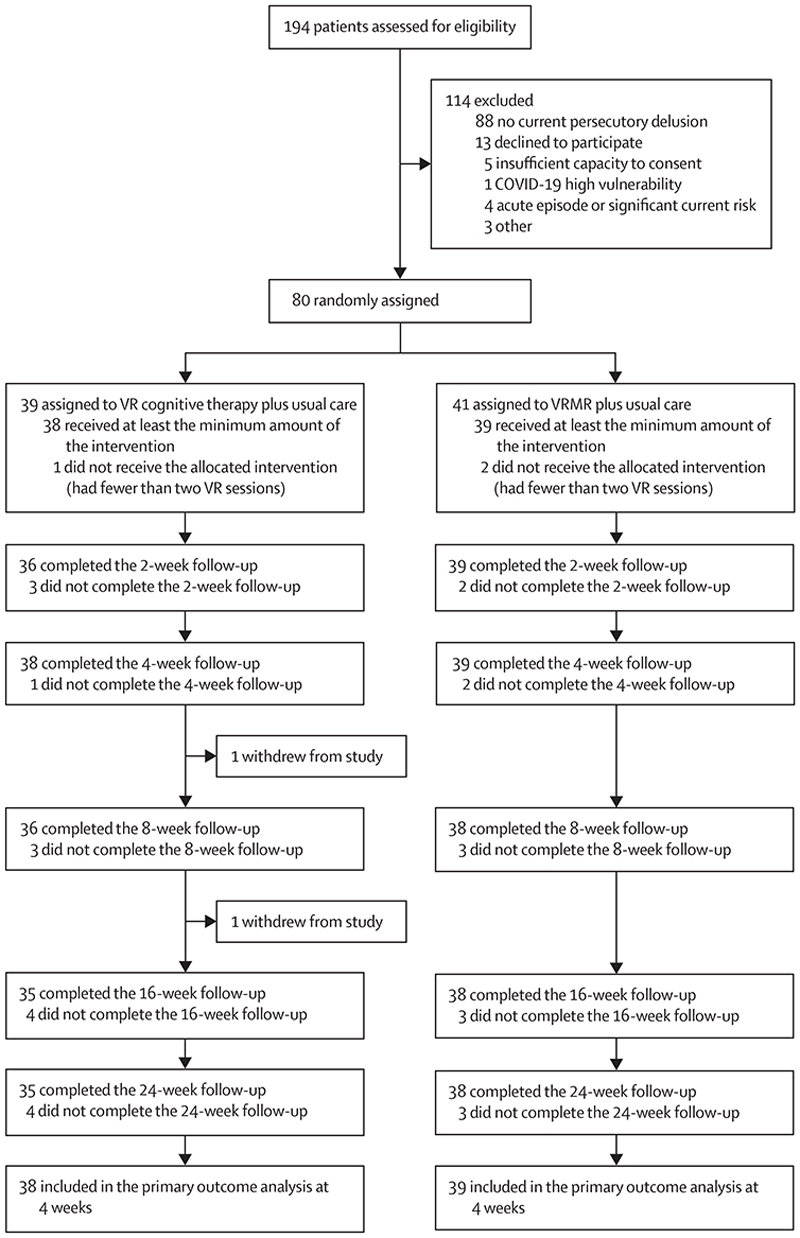
Trial profile VR=virtual reality. VRMR=VR mental relaxation.

**Table 1:** Baseline characteristics

	THRIVE VR cognitive treatment (n=39)	THRIVE VR mental relaxation (n=41)	Total (n=80)
Age, years	41·1 (13·4)	39·4 (13·0)	40·3 (13·1)
Gender			
Men	25 (64·1%)	24 (58·5%)	49 (61·3%)
Women	14 (35·9%)	17 (41·5%)	31 (38·8%)
Relationship status			
Single	26 (66·7%)	31 (75·6%)	57 (71·3%)
Cohabiting	3 (7·7%)	3 (7·3%)	6 (7·5%)
Married or civil partnership	7 (17·9%)	5 (12·2%)	12 (15·0%)
Divorced	3 (7·7%)	2 (4·9%)	5 (6·3%)
Ethnicity			
White	28 (71·8%)	36 (87·8%)	64 (80·0%)
Black Caribbean	3 (7·7%)	0	3 (3·8%)
Black African	2 (5·1%)	0	2 (2·5%)
Black other	1 (2·6%)	0	1 (1·3%)
Indian	1 (2·6%)	0	1 (1·3%)
Pakistani	2 (5·1%)	1 (2·4%)	3 (3·8%)
Chinese	1 (2·6%)	0	1 (1·3%)
Other	1 (2·6%)	4 (9·8%)	5 (6·3%)
Employment			
Unemployed	32 (82·1%)	36 (87·8%)	68 (85·0%)
Employed full-time	1 (2·6%)	··	1 (1·3%)
Employed part-time	3 (7·7%)	1 (2·4%)	4 (5·0%)
Self-employed	1 (2·6%)	1 (2·4%)	2 (2·5%)
Retired	2 (5·1%)	2 (4·9%)	4 (5·0%)
Housewife or househusband	0	1 (2·4%)	1 (1·3%)
Usual living arrangement			
Living alone (with or without children)	15 (38·5%)	17 (41·5%)	32 (40·0%)
Living with husband or wife (with or without children)	8 (20·5%)	5 (12·2%)	13 (16·3%)
Living together as a couple	2 (5·1%)	2 (4·9%)	4 (5·0%)
Living with parents	7 (17·9%)	12 (29·3%)	19 (23·8%)
Living with other relatives	3 (7·7%)	1 (2·4%)	4 (5·0%)
Living with others	4 (10·3%)	4 (9·8%)	8 (10·0%)
Diagnosis			
Schizophrenia	20 (51·3%)	16 (39·0%)	36 (45·0%)
Schizo-affective disorder	5 (12·8%)	9 (22·0%)	14 (17·5%)
Delusional disorder	2 (5·1%)	3 (7·3%)	5 (6·3%)
Psychosis not otherwise specified	12 (30·8%)	13 (31·7%)	25 (31·3%)
Prescribed an antipsychotic medication			
No	1 (2·6%)	4 (9·8%)	5 (6·3%)
Yes	38 (97·4%)	37 (90·2%)	75 (93·8%)
Prescribed an antidepressant medication			
No	11 (28·2%)	15 (36·6%)	26 (32·5%)
Yes	28 (71·8%)	26 (63·4%)	54 (67·5%)
Prescribed an anxiolytic medication			
No	34 (87·2%)	35 (85·4%)	69 (86·3%)
Yes	5 (12·8%)	6 (14·6%)	11 (13·8%)
Prescribed a mood stabiliser medication			
No	31 (79·5%)	33 (80·5%)	64 (80·0%)
Yes	8 (20·5%)	8 (19·5%)	16 (20·0%)
Severity of delusion			
Moderate	18 (46·2%)	18 (43·9%)	36 (45·0%)
High	21 (53·8%)	23 (56·1%)	44 (55·0%)

THRIVE=Therapeutic Realistic Immersive Virtual Environments. VR=virtual reality.

**Table 2:** Summary statistics for the primary and secondary outcomes

	THRIVE VR cognitive treatment	THRIVE VR mental relaxation	Adjusted mean difference (95% CI)*	p value
**Primary outcome**				
Persecutory delusion conviction				
Baseline	78·1 (18·1); n=39 (100%)	79·0 (15·5); n=41 (100%)	··	··
2 weeks	62·4 (23·9); n=36 (92%)	65·2 (19·3); n=39 (95%)	–0·94 (–11·63 to 9·75); SES –0·06 (–0·70 to 0·58)	0·86
4 weeks	52·1 (23·8); n=38 (97%)	56·6 (25·0); n=39 (95%)	–2·16 (–12·77 to 8·44); SES –0·13 (–0·76 to 0·50)	0·69
8 weeks	53 V (25·0); n=36 (92%)	55·7 (26·6); n=38 (93%)	0·93 (–9·80 to 11·66); SES 0·06 (–0·59 to 0·70)	0·87
16 weeks	53·3 (23·5); n=35 (90%)	49·7 (28·9); n=38 (93%)	5·78 (–5·00 to 16·56); SES 0·35 (–0·30 to 0·99)	0·29
24 weeks	51·9 (27·4); n=35 (90%)	53·0 (29·0); n=38 (93%)	–1·37 (–12·12 to 9·39); SES –0·08 (–0·72 to 0·56)	0·80
**Secondary outcomes**				
R-GPTS Part A (paranoia, social reference) score				
Baseline	15·1 (8·0); n=39 (100%)	15·5 (7·4); n=41 (100%)	··	··
2 weeks	14·2 (7·7); n=36 (92%)	12·7 (6·8); n=39 (95%)	2·11 (–0·75 to 4·96)	0·15
4 weeks	11·3 (7·5); n=37 (95%)	11·7 (7·6); n=39 (95%)	0·55 (–2·31 to 3·40)	0·71
8 weeks	11·7 (7·5); n=36 (92%)	11·4 (7·7); n=38 (93%)	1·08 (–1·79 to 3·96)	0·46
16 weeks	10·0 (7·8); n=34 (87%)	10·7 (7·8); n=38 (93%)	0·38 (–2·52 to 3·27)	0·80
24 weeks	10·6 (8·8); n=34 (87%)	11·4 (8·1); n=37 (90%)	–0·66 (–3·56 to 2·24)	0·66
R-GPTS Part B (paranoia, persecution) score				
Baseline	23·7 (8·1); n=39 (100%)	21·3 (8·8); n=41 (100%)	··	··
2 weeks	20·9 (9·7); n=36 (92%)	18·0 (9·4); n=39 (95%)	2·16 (–1·82 to 6·14)	0·29
4 weeks	16·5 (9·8); n=37 (95%)	16·0 (10·1); n=39 (95%)	–0·51 (–4·48 to 3·46)	0·80
8 weeks	17·1 (9·7); n=36 (92%)	15·0 (10·2); n=38 (93%)	1·17 (–2·83 to 5·17)	0·57
16 weeks	14·1 (10·5); n=34 (87%)	14·3 (11·7); n=38 (93%)	–0·98 (–5·01 to 3·05)	0·63
24 weeks	15·1 (13·1); n=34 (87%)	14·9 (10·8); n=37 (90%)	–1·60 (–5·64 to 2·45)	0·44
R-GPTS total score (paranoia)				
Baseline	38·8 (15·1); n=39 (100%)	36·8 (14·6); n=41 (100%)	··	··
2 weeks	35·1 (16·7); n=36 (92%)	30·7 (14·7); n=39 (95%)	4·38 (–2·01 to 10·76)	0·18
4 weeks	27·9 (16·1); n=37 (95%)	27·8 (16·5); n=39 (95%)	0·17 (–6·20 to 6·55)	0·96
8 weeks	28·8 (16·0); n=36 (92%)	26·4 (16·2); n=38 (93%)	2·39 (–4·02 to 8·81)	0·47
16 weeks	24·2 (17·6); n=34 (87%)	25·0 (18·4); n=38 (93%)	–0·48 (–6·94 to 5·98)	0·89
24 weeks	25·8 (21·3); n=34 (87%)	26·3 (18·2); n=37 (90%)	–2·15 (–8·63 to 4·32)	0·52
PSYRATS score				
Baseline	16·9 (3·2); n=39 (100%)	16·9 (3·5); n=41 (100%)	··	··
2 weeks	14·4 (4·2); n=36 (92%)	15·6 (3·8); n=39 (95%)	–0·91 (–2·74 to 0·92)	0·33
4 weeks	12·9 (4·4); n=37 (95%)	13·8 (4·6); n=38 (93%)	–0·73 (–2·57 to 1·10)	0·43
8 weeks	13·0 (4·1); n=36 (92%)	13·9 (4·4); n=38 (93%)	–0·68 (–2·52 to 1·16)	0·47
16 weeks	12·6 (4·5); n=35 (90%)	13·0 (5·4); n=38 (93%)	–0·26 (–2·11 to 1·58)	0·78
24 weeks	12·4 (5·9); n=34 (87%)	13·0 (5·5); n=36 (88%)	–0·99 (–2·86 to 0·88)	0·30
O-BAT maximum number of steps avoided†				
Baseline	2·5 (1·4); n=30 (77%)	2·3 (1·6); n=26 (63%)	··	··
4 weeks	2·4 (1·9); n=22 (56%)	1·6 (1·8); n=21 (51%)	0·83 (–0·19 to 1·85)	0·11
O-BAT mean distress score				
Baseline	5·2 (2·2); n=28 (72%)	6·0 (1·8); n=24 (59%)	··	··
4 weeks	4·5 (2·5); n=17 (44%)	4·6 (1·9); n=18 (44%)	0·06 (–1·33 to 1·45)	0·93
Actigraphy mean number of steps (daily)				
Baseline	5060·9 (3509·4); n=29 (74%)	5038·7 (4137·4); n=17 (41%)	··	··
4 weeks	4511·9 (3448·8); n=19 (49%)	4239·4 (4187·8); n=17 (41%)	–418·3 (–1925·2 to 1088·6)	0·59
24 weeks	5827·3 (4765·5); n=10 (26%)	3138·1 (3287·1); n=12 (29%)	121·7 (–1557·2 to 1800·5)	0·89
Time budget score				
Baseline	49·3 (15·4); n=35 (90%)	47·7 (13·3); n=36 (88%)	··	··
4 weeks	48·9 (16·7); n=29 (74%)	46·9 (14·2); n=34 (83%)	1·83 (–4·33 to 7·98)	0·56
24 weeks	49·0 (17·5); n=25 (64%)	49·6 (14·7); n=29 (71%)	–1·94 (–8·52 to 4·64)	0·56
EQ-5D-5L index				
Baseline	0·5 (0·3); n=36 (92%)	0·5 (0·2); n=38 (93%)	··	··
4 weeks	0·6 (0·3); n=36 (92%)	0·6 (0·3); n=37 (90%)	0·01 (–0·09 to 0·11)	0·89
24 weeks	0·6 (0·3); n=32 (82%)	0·5 (0·3); n=34 (83%)	0·04 (–0·06 to 0·14)	0·43
EQ-5D-5L visual analogue scale				
Baseline	51·9 (19·7); n=36 (92%)	42·7 (23·3); n=38 (93%)	··	··
4 weeks	58·1 (20·5); n=36 (92%)	54·0 (23·9); n=37 (90%)	–0·75 (–9·24 to 7·75)	0·86
24 weeks	58·8 (23·8); n=32 (82%)	52·0 (20·3); n=33 (80%)	0·85 (–8·17 to 9·88)	0·85
C-SSRS total scoret				
Baseline	0·7 (1·0); n=37 (95%)	0·9 (1·2); n=37 (90%)	··	··
4 weeks	0·5 (1·0); n=35 (90%)	0·5 (0·9); n=35 (85%)	0·12 (–0·29 to 0·52)	0·57
24 weeks	0·6 (1·2); n=31 (X%)	0·8 (1·2); n=32 (78%)	–0·22 (–0·85 to 0·41)	0·50
WEMWBS score				
Baseline	33·7 (8·9); n=38 (97%)	34·6 (8·6); n=37 (90%)	··	··
4 weeks	38·6 (10·4); n=36 (92%)	38·7 (10·1); n=38 (93%)	0·27 (–2·97 to 3·50)	0·87
24 weeks	38·9 (10·7); n=32 (79%)	38·1 (9·9); n=33 (80%)	0·96 (–2·42 to 4·34)	0·58
QPR total score				
Baseline	25·1 (10·4); n=37 (95%)	25·4 (10·0); n=41 (100%)	··	··
4 weeks	28·7 (11·9); n=36 (92%)	30·1 (11·1); n=39 (95%)	–1·27 (–5·18 to 2·64)	0·52
24 weeks	31·5 (11·8); n=33 (85%)	29·7 (12·0); n=36 (88%)	2·29 (–1·74 to 6·31)	0·27

Data are mean (SD), unless otherwise indicated. C-SSRS=Columbia Suicide Severity Rating Scale. O-BAT=Oxford-Behavioural Avoidance Task. QPR=Questionnaire about the Process of Recovery. R-GPTS=Revised-Green et al Paranoid Thoughts Scale. SES=standardised effect size. THRIVE=Therapeutic Realistic Immersive Virtual Environments. VR=virtual reality. WEMWBS=Warwick-Edinburgh Mental Wellbeing Scale.

* For the primary outcome (VR cognitive treatment *vs* VR mental relaxation), the mean difference was estimated from a linear mixed-effects model adjusting for baseline conviction as a fixed effect and participant as the random effect. SES was the estimated mean difference divided by baseline SD. For secondary outcomes, the mean difference was estimated from a linear mixed-effects model adjusting for severity of delusion (binary) and baseline values of the outcome as fixed effects and participant as the random effect for all outcomes except for the O-BAT outcomes, which were fitted using linear regression. Estimates and CIs for C-SSRS were obtained using bootstrapping as the distribution of the outcome and residuals were skewed.

† Baseline values of the outcomes are modelled as a categorical measure rather than continuous measures.

**Table 3:** Mediation analysis on persecutory belief conviction using mixed effects models

	2 weeks (mid-treatment)	4 weeks (end of treatment)
**Defence behaviours**		
Total effect	–1·61 (–12·66 to 9·45); p=0·78	–0·07 (–11·03 to 10·89); p=0·99
Direct effect	–0·66 (–11·04 to 9·72); p=0·90	2·27 (–8·10 to 12·65); p=0·67
Indirect effect	0·51 (–2·06 to 3·08); p=0·70	–0·52 (–3·09 to 2·05); p=0·69
Path A (group allocation to mechanism)	0·70 (–2·80 to 4·20); p=0·70	–0·71 (–4·21 to 2·79); p=0·69
Path B (mechanism to treatment outcome)	0·73 (0·42 to 1·04); p<0·001	0·73 (0·42 to 1·04); p<0·001
Proportion mediated*	0·32	7·51
**Safety beliefs**		
Total effect	2·07 (–8·60 to 12·74); p=0·70	3·59 (–7·00 to 14·17); p=0·51
Direct effect	2·68 (–7·52 to 12·89); p=0·61	3·06 (–7·12 to 13·24); p=0·56
Indirect effect	–0·46 (–2·62 to 1·71); p=0·68	1·16 (–1·06 to 3·37); p=0·31
Path A (group allocation to mechanism)	2·32 (–8·66 to 13·30); p=0·68	–5·89 (–16·83 to 5·05); p=0·29
Path B (mechanism to treatment outcome)	–0·20 (–0·29 to –0·11); p<0·001	–0·20 (–0·29 to –0·11); p<0·001
Proportion mediated*	0·22	0·32

Data are estimate (95% CI), unless otherwise indicated.

* Indirect/total effect.

**Table 4:** Provision of usual care

	THRIVE VR cognitive treatment (n=39)	THRIVE VR mental relaxation (n=41)
**Antipsychotic chlorpromazine equivalent mean dose, mg per day**		
Baseline	605·1 (447·7); n=38 (97%)	470·2 (314·2); n=37 (90%)
4 weeks	610·9 (454·3); n=38 (97%)	480·1 (308·6); n=37 (90%)
24 weeks	618·7 (418·3); n=37 (95%)	501·4 (318·0); n=38 (93%)
**Patients who had a psychiatric hospital admission, n**		
24 weeks before baseline	2 (5%)	5 (12%)
0–4 weeks	0	0
4–24 weeks	2 (5%)	1 (2%)
**Patients who had psychological therapy outside the trial, n**		
24 weeks before baseline	8 (21%)	6 (15%)
0–4 weeks	3 (8%)	1 (2%)
4–24 weeks	4 (10%)	7 (17%)
**Number of hours of psychological therapy outside the trial**		
24 weeks before baseline	1·2 (2·9)	0·8 (2·5)
0–4 weeks	0·1 (0·5)	0·1 (0·3)
4–24 weeks	0·5 (2·4)	0·8 (2·0)
**Number of psychiatrist appointments**		
24 weeks before baseline	1·1 (1·6); n=33 (85%)	1·9 (1·4); n=37 (90%)
0–24 weeks	1·2 (1·4); n=30 (77%)	1·3 (1·0); n=31 (76%)
**Number of community psychiatric nurse appointments**		
24 weeks before baseline	7·6 (8·5); n=33 (85%)	5·8 (6·3); n=36 (88%)
0–24 weeks	6·5 (6·9); n=29 (74%)	6·9 (7·9); n=32 (78%)
**Number of social worker appointments**		
24 weeks before baseline	0·7 (2·1); n=37 (95%)	1·8 (5·4); n=37 (90%)
0–24 weeks	0·6 (1·7); n=30 (77%)	1·8 (4·5); n=31 (76%)

Data are mean (SD), unless otherwise indicated. THRIVE=Therapeutic Realistic Immersive Virtual Environments. VR=virtual reality.

**Table 5:** Summary of adverse events by sex

	THRIVE VR cognitive treatment (n=39)	THRIVE VR mental relaxation (n=41)
**Adverse events**		
Male	3 (1 [3%])	0
Female	1 (1 [3%])	3 (2 [5%])
**Serious adverse events**		
Male	6 (4 [10%])	3 (3 [7%])
Female	4 (3 [8%])	4 (2 [5%])

Data are number of events or number of patients (%). THRIVE=Therapeutic Realistic Immersive Virtual Environments. VR=virtual reality.

## Data Availability

De-identified participant data will be available in anonymised form from the corresponding author (DF) on reasonable request (including a study outline), subject to review and contract with the University of Oxford, following the publication of results. The trial protocol is published. The data analysis plan and the full statistical report are available in the appendix.

## Research in context

### Evidence before this study

Persecutory delusions are a key difficulty experienced by patients diagnosed with severe mental health difficulties. This severe form of paranoia often does not respond sufficiently to standard pharmacological and psychological treatments. On Jan 25, 2023, we searched PubMed without date or language restrictions using the following search terms: (psychosis OR psychotic OR schizophrenia) AND (virtual reality OR VR) AND (delusions OR persecutory). 46 papers were identified. We found no clinical trials of a VR intervention for patients selected for the presence of persecutory delusions. An experimental study with 30 patients with persecutory delusions had taken place, which was the proof-of-concept test for the current trial. A randomised controlled clinical trial in the Netherlands showed that 16 sessions with a CBT therapist that included VR reduced paranoia for patients selected for having at least mild paranoid ideation. Our group’s six-session automated VR therapy for agoraphobic avoidance (gameChange) produced significant improvements in paranoia for patients diagnosed with psychosis who had severe agoraphobia.

### Added value of this study

To our knowledge, we did the first clinical trial using virtual reality (VR) to treat persistent persecutory delusions. We tested a new four-session automated VR cognitive intervention for patients with persistent persecutory delusions in the context of psychosis against a credible alternative VR therapy (mental relaxation) that used different scenarios and intervention techniques. We found large reductions in the severity of persecutory delusions but no difference in outcomes between the two VR interventions. Similarly, we found moderate to large reductions in the targeted cognitive processes, but no differences in mechanistic outcomes between the two VR interventions. Two VR interventions with different content were found to perform similarly. Higher patient ratings of the credibility of a VR intervention and higher expectations of improvement predicted a small amount of the reductions in the persecutory delusions.

### Implications of all the available evidence

VR interventions are safe and have high uptake in patients with psychosis. Delivery of automated therapy within VR, supported by a mental health professional, is feasible for patients with current and long-standing psychotic experiences. However, the results of the trial pose a challenge. Two VR interventions, different in content and targeted to different cognitive mechanisms, had similar effects on cognitive processes and outcomes for patients with delusions. This finding implies that the development of immersive technologies for mental health would benefit from sustained research into the implementation of various techniques, including the necessary doses, and the effects of these techniques on various potential mechanisms of action. Just as for other treatments, VR therapies also need to be designed to foster accurate belief in intervention credibility and expectancy.
