# Supplementary material for: Automated virtual reality cognitive therapy versus virtual reality mental relaxation therapy for the treatment of persistent persecutory delusions in patients with psychosis (THRIVE): a parallel-group, single-blind, randomised controlled trial in England with mediation analyses
Source: Lancet Psychiatry. Author manuscript; Available in PMC 2025 Jul 8. (PMC7617886; doi:10.1016/S2215-0366(23)00257-2)
Supplement: Supplementary appendix [file EMS206644-supplement-Supplementary_appendix.pdf]

THE LANCET
Psychiatry

Supplementary appendix

This appendix formed part of the original submission and has been peer reviewed. We post it as supplied by the authors.

Supplement to: Freeman D, Lister R, Waite F, et al. Automated virtual reality cognitive therapy versus virtual reality mental relaxation therapy for the treatment of persistent persecutory delusions in patients with psychosis (THRIVE): a parallel-group, single-blind, randomised controlled trial in England with mediation analyses. *Lancet Psychiatry* 2023; published online Sept 21. https://doi.org/10.1016/S2215-0366(23)00257-2.

Supplementary materials

Protocol pp 2-33

Statistical Analysis plan pp 35-66

Statistical Analysis report pp 68-124

Graph of delusion change pp 126

© Copyright: The University of Oxford and Oxford University Hospitals NHS Foundation Trust 2016

Trial Title: The THRIVE study: A randomized controlled trial comparing Virtual Reality Confidence Building with VR Mental Relaxation for people with fears about others.

Ethics Ref: 18/SC/0316

Date and Version No: 28th April 2021, version 4.3

Chief Investigator: Professor Daniel Freeman (University of Oxford)

Investigators: Professor David Clark (co-investigator, University of Oxford), Professor Melvyn Slater (co-investigator, University College London), Professor Graham Dunn (co-investigator, University of Manchester), Professor Ly-Mee Yu (study statistician, University of Oxford), Dr Felicity Waite (co-investigator, University of Oxford) and Dr Laina Rosebrock (trial co-ordinator, University of Oxford)

Sponsor: University of Oxford

Funder: Medical Research Council Developmental Pathway Funding Scheme (MRC DPFS)

Chief Investigator Signature:

Statistician Signature:

Potential conflicts of interest:

The principal investigator (Daniel Freeman) and co-investigator (Mel Slater) are founders and non-executive directors of the Oxford University spin out company, OxfordVR. Additionally, the sponsor (University of Oxford) is a stakeholder in this company. There is the option to licence the VR treatment developed in this project (intellectual property) to a commercial partner such as OxfordVR. This represents a potential conflict of interest.

Confidentiality Statement

This document contains confidential information that must not be disclosed to anyone other than the Sponsor, the Investigator's Team, HRA, host organisation, Medical Health and Research Council, and members of the Research Ethics Committee, unless authorised to do so.

© Copyright: The University of Oxford and Oxford University Hospitals NHS Foundation Trust 2016

TABLE OF CONTENTS

1. KEY TRIAL CONTACTS 5
2. SYNOPSIS 6
3. ABBREVIATIONS 7
4. BACKGROUND AND RATIONALE 7
5. OBJECTIVES AND OUTCOME MEASURES 9
6. TRIAL DESIGN 11
7. PARTICIPANT IDENTIFICATION 12
   7.1. Trial Participants 12
   7.2. Inclusion Criteria 12
   7.3. Exclusion Criteria 12
8. TRIAL PROCEDURES 12
   8.1. Recruitment 13
   8.2. Informed Consent 13
   8.3. Randomisation, blinding and code-breaking 13
   8.4. Baseline Assessments 14
   8.5. Subsequent Visits 14
   8.6. Discontinuation/Withdrawal of Participants from Trial 15
   8.7. Definition of End of Trial 15
   8.8. Considerations Related to COVID-19 15
9. PSYCHOLOGICAL TREATMENTS 16
   9.1. Description 16
   9.2 Device details 17
   9.3 Compliance with Trial Interventions 18
10 SAFETY REPORTING 19
   10.1 Definitions of Adverse Events 19
   10.2 Definitions of Serious Adverse Events 19
   10.3 Adverse Device Effect (ADE) 20
   10.4 Serious Adverse Device Effect (SADE) 20
   10.5 Device Deficiencies 21
   10.6 Anticipated Serious Adverse Device Effect 21
   10.7 Unanticipated Serious Adverse Device Effect 21
   10.8 Causality 21
   10.9 Reporting Procedures for Adverse Events 21
   Reporting to the DMEC 23

© Copyright: The University of Oxford and Oxford University Hospitals NHS Foundation Trust 2016

Reporting to the Research Ethics Committee 23
10.10 Safety Monitoring Committee 23
11 STATISTICS 23
12 DATA MANAGEMENT 24
12.1 Source Data 25
12.2 Access to Data 25
12.3 Data Recording and Record Keeping 25
13 QUALITY ASSURANCE PROCEDURES 25
14 ETHICAL AND REGULATORY CONSIDERATIONS 25
14.1 Declaration of Helsinki 25
14.2 Guidelines for Good Clinical Practice 25
14.3 Approvals 25
14.4 Reporting 26
14.5 Participant Confidentiality 26
14.6 Expenses and Benefits 26
14.7 Ethical Considerations 26
14.8 Other Considerations 27
15 FINANCE AND INSURANCE 27
15.1 Funding 27
15.2 Insurance 27
16 PUBLICATION POLICY 27
17 REFERENCES 27
18 APPENDIX A: SCHEDULE OF PROCEDURES 30
19 APPENDIX B: AMENDMENT HISTORY 31

© Copyright: The University of Oxford and Oxford University Hospitals NHS Foundation Trust 2016

1. KEY TRIAL CONTACTS

**Chief Investigator**	Prof Daniel Freeman, Department of Psychiatry, University of Oxford, Warneford Hospital, Oxford OX3 7JX. Email: Daniel.freeman@psych.ox.ac.uk. Tel. 01865 226490
**Sponsor**	Heather House, Clinical Trials Research Governance, Joint Research Office, University of Oxford, Boundary Brook House, Churchill Drive, Headington, Oxford, OX3 7LQ Email: ctrg@admin.ox.ac.uk. Tel 01865 289886
**Statistician**	Prof Ly-Mee Yu, Deputy Director of Primary Care Clinical Trials Unit (CTU), Nuffield Department of Primary Care Health Sciences, Medical Sciences Division, University of Oxford, Radcliffe Observatory Quarter, Woodstock Road, Oxford. OX2 6GG. Email: ly-mee.yu@phc.ox.ac.uk. Tel 01865 617199.
**Monitor**	Clinical Trials & Research Governance<br>Joint Research Office, University of Oxford<br>Boundary Brook House<br>Churchill Drive, Headington<br>Oxford, OX3 7LQ
**Committees**	Data monitoring and ethics committee (DMEC).

© Copyright: The University of Oxford and Oxford University Hospitals NHS Foundation Trust 2016

2. SYNOPSIS

Trial Title	The THRIVE study: A randomized controlled trial comparing Virtual Reality Confidence Building with VR Mental Relaxation for people with fears about others.	
Funding	Medical Research Council, Developmental Pathway Funding Scheme (MRC DPFS)	
Trial Design	Randomisation to Virtual Reality Confidence Building (VRCB) or Virtual Reality Mental Relaxation (VRMR). Standard NHS care continues as usual in both arms.	
Trial Participants	Patients with persistent persecutory delusions (despite receiving treatment from services) in the context of non-affective psychosis (typically schizophrenia diagnosis).	
Planned Sample Size	90 patients in the trial, inclusive of 30 patients for an interim analysis.	
Treatment duration	4 weeks	
Follow up duration	20 weeks	
Planned Trial Period	24 weeks (per patient)	
	Objectives	Outcome Measures
Primary	Test improvements in persistent persecutory delusions by treatment type.	Conviction in the delusion using 0-100% scale.
Secondary	1. Test clinical improvements by treatment type in real world delusion related distress.<br><br>2. Test clinical improvements by treatment type in activity levels, quality of life, paranoia, and suicidal ideation.<br><br>3. Test clinical improvements in delusional severity, wellbeing, and perceptions of recovery.<br>4. Test maintenance over time of improvements listed above.	1. O-BAT (Freeman et al., 2016), which provides scores of avoidance and distress (rated on a 0-10 scale).<br><br>2. Actigraphy and time budget (Jolley et al., 2006); EQ-5D-5L (http://www.euroqol.org/); Revised-GPTS (Green et al., 2008; Freeman et al., 2019); Columbia-Suicide Severity Rating Scale (Posner et al., 2011).<br>3. PSYRATS; (Haddock et al, 1999); WEMWBS (Tennant et al., 2007); QPR (Neil et al., 2009).<br>4. As above
Tertiary	Test mediation of treatment effects by changes in safety beliefs and defence behaviours.	Safety Behaviours Questionnaire (Freeman et al., 2001; strength of safety beliefs will be assessed using a visual analogue scale (Freeman et al., 2016).

© Copyright: The University of Oxford and Oxford University Hospitals NHS Foundation Trust 2016

3. ABBREVIATIONS

ADE	Adverse Device Event
AE	Adverse event
AR	Adverse reaction
CI	Chief Investigator
CONSORT	Consolidated Standards of Reporting Trials
CRF	Case Report Form
CTRG	Clinical Trials and Research Governance
DMEC	Data Monitoring and Ethics Committee
GCP	Good Clinical Practice
GP	General Practitioner
HRA	Health Research Authority
MHRA	Medicines and Healthcare products Regulatory Agency
NHS	National Health Service
PI	Principal Investigator
REC	Research Ethics Committee
SADE	Serious Adverse Device Effect
SAE	Serious Adverse Event
SAR	Serious Adverse Reaction
SOP	Standard Operating Procedure
SUSAR	Suspected Unexpected Serious Adverse Reactions
VR	Virtual Reality
VRCB	Virtual Reality Confidence Building (the device)
VRMR	Virtual Reality Mental Relaxation – shorthand for the control condition

4. BACKGROUND AND RATIONALE

The trial is funded by the Medical Research Council Developmental Pathway Funding Scheme (MRC DPFS) and has received substantial scientific review as part of the funding application.

The clinical problem

Persecutory delusions are unfounded beliefs that others are trying to harm the person (e.g. 'People know what I'm thinking and will kill me'). Approximately 220,000 people in England and Wales have a diagnosis of schizophrenia and about 70% of patients with schizophrenia have this psychotic experience. Persecutory delusions have a substantial impact for patients; they typically lead to social withdrawal and predict hospital admission (Castle et al., 1994) and even suicide (Hor & Taylor, 2010). The total annual cost to the

© Copyright: The University of Oxford and Oxford University Hospitals NHS Foundation Trust 2016

public sector in England is over £7 billion. Life expectancy is, on average 14.5 years shorter for people with these problems (Hjorthøj et al, 2017).

Approximately half of patients do not respond adequately to the first line treatment, medication, and residual problems are very common. In a review, Kennedy et al (2014) found that 'almost 60% of patients failed to achieve response after 23 weeks on antipsychotic drug therapy.' Meta-analysis for first generation psychological treatment indicates only small effects for delusions (g=0.36; van der Gaag et al., 2014). This suggests that psychological treatment also needs improvement. Part of the problem is that schizophrenia is an umbrella term, comprising multiple independent problems. These individual psychotic experiences, such as persecutory delusions, need separating out in treatment development. This allows key maintenance factors to be targeted. This approach has been highly successful (Freeman et al., 2015), however there is a shortage of qualified therapists to deliver face to face interventions. With 81% of NHS patients never having received Cognitive Behavioural Therapy (CBT), the 2014 National Audit of Schizophrenia calls for NHS Trusts to increase access to evidence-based psychological interventions. Therefore, more efficacious and accessible interventions are urgently required.

Translational studies leading to the trial

The chief investigator and colleagues have developed a rigorously tested theoretical model of persecutory delusions (Freeman et al., 2016). At the core of the delusion is a belief of being unsafe; this is developed in the context of genetic and environmental risk and maintained by a number of factors, including 'defence behaviours'. When patients use these defence behaviours (such as avoidance, looking for escape routes, or taking steps to decrease their visibility when out), absence of harm is attributed to defence behaviours rather than threat belief inaccuracy. Over time, these types of behaviours are associated with increased distress and threat appraisals (e.g. Tully et al., 2016). Therefore, patients need to go into feared situations and drop defence behaviours to re-learn safety. However, patients often find it too difficult to do this.

A solution is virtual reality. Patients find it much easier to enter VR environments because they consciously know that it is not real. Nonetheless, VR elicits responses comparable to those in the real world (e.g. Gorini et al., 2015) and the new learning of safety transfers into everyday life (Morina et al., 2015). VR directly tackles the key underlying mechanism of persecutory delusions: unfounded threat beliefs. VR enables patients to go into the situations that they fear, drop their defences, and truly learn that the fears are unrealistic. It builds experience of safety, self-confidence, and reversal of social withdrawal.

Virtual reality has been successfully shown to treat anxiety disorders. Meta-analyses for VR exposure in the treatment of anxiety disorders, show that the effects are large (d=1.1), maintain over years, generalise to real life, and are as good as using real life exposure (Opris et al, 2012; Morina et al, 2015). It is also the most popular treatment choice when offered, preferred over real life exposure (e.g. Garcia-Palacios et al, 2007). Using VR, patients with anxiety disorders can go into feared situations and drop defences; this helps patients learn, by direct experience, that they are safe and that fears are unrealistic.

VR may be used in a similar way, to help patients with psychosis re-learn safety. In a pilot study (Freeman et al., 2016), conducted by the research team, a single session of our theoretically driven VR cognitive treatment (going into VR feared situations with dropping of defence behaviours) led to a large reduction in the delusions (d=1.3) directly compared to an alternative active treatment (VR exposure treatment). Benefits transferred to the real world; VR cognitive treatment led to a 19.6% greater reduction in distress following a real world behavioural task compared to VR exposure. The pilot study included 30 patients with persecutory delusions, using a lab-based VR treatment.

© Copyright: The University of Oxford and Oxford University Hospitals NHS Foundation Trust 2016

The proposed trial

The proposed study will test a more comprehensive, affordable and portable, 'plug and play' version of the VR cognitive treatment: VR Confidence Building (VRCB). This will be tested in a larger sample of patients.

Aims: The key question to test is: Does VRCB lead to greater reduction in delusional conviction, and improvements in activity levels, quality of life, and suicidal ideation, compared to the control condition (VR mental relaxation; VRMR)?

Method: The study is a randomised controlled trial (RCT) for patients who have persecutory delusions involving feeling threatened when with other people. Initially, effect sizes will be re-established by an interim analysis of 30 patients, randomised to four sessions of VRCB or VRMR. If the interim effect size suggests that the treatment is worth pursuing (d >0.1), then we will continue the trial, testing 90 patients in total, to assess the treatment in a Phase IIa RCT. Medication prescription will continue as usual. Assessments, by a rater blind to allocation, will be conducted at 0, 2, 4 (post treatment), 8, 16, and 24 weeks. The primary outcome is level of delusional conviction. Secondary outcomes include real world distress, activity levels, suicidal ideation, and quality of life. Mediation will also be tested. All main analyses will be intention-to-treat.

The 'doseage' of VRCB has been guided by VR treatment development for anxiety disorders (four sessions is most commonly used), our clinical experience with patients with psychosis, and feedback from patients with persecutory delusions. The number of sessions and session length for VR mental relaxation was chosen to match VRCB, to control for the time spent in VR. Relaxation is frequently used in clinical practice with clients with psychosis and there is some evidence for beneficial effects (e.g. Vancampfort et al., 2012, Kavak et al., 2016). We will use a set of relaxing VR environments, which are commercially available for anyone to download, for participants to view while practicing simple relaxation techniques. VR mental relaxation and VRCB have both been rated as credible, from patient feedback.

Our group has been pioneering VR for paranoia, since 2003. We have used VR with over 50 patients with persecutory delusions and several hundred members of the general population with no side effects. Additionally, VR is very popular with patients. In July 2017 we conducted a survey concerning VR and social difficulties with more than 50 patients in local NHS psychosis services; 88% said they would like to try VR.

Please note: The aims of the trial are around assessing efficacy of the treatment. However, as VRCB comes under the medical devices regulations, a Notice of No Objection will be sought for using this treatment in the trial (see cover letter to MHRA for further details).

5. OBJECTIVES AND OUTCOME MEASURES

**Objectives**<br><br>To test the following hypothesis:	**Outcome Measures**	**Timepoint(s) of evaluation of this**

© Copyright: The University of Oxford and Oxford University Hospitals NHS Foundation Trust 2016

		outcome measure (if applicable)
**Primary hypothesis:**<br>VRCB will lead a reduction in delusional conviction compared to VR mental relaxation.	The primary outcome measure will be conviction in the persecutory delusion (using a 0–100% scale).	0, 2, 4, 8, 16, and 24 weeks (primary end point 4 weeks)
**Secondary Hypotheses:**<br><br>1. At 4 weeks, VRCB, compared to VRMR, will lead to a reduction in distress in real world situations.<br><br>2. At 4 weeks, VRCB, compared to VRMR, will lead to an increase in activity, quality of life, and a reduction in suicide ideation and overall paranoia.<br><br>3. VRCB, compared to VRMR, will lead to improvements in delusional severity, wellbeing, and perceptions of recovery.<br><br>4. Benefits of VRCB will be maintained over time.	1. Real world distress related to the persecutory delusion will be assessed using a behavioural avoidance task (O-BAT; Freeman et al., 2016). Participants create a 5 step hierarchy of real world situations and rate levels of distress (on a scale from 0 (not distressed at all) to 10 (extremely distressed)), resulting in scores of avoidance and distress.<br><br>2. Activity will be assessed by actigraphy and a time-budget measure (Jolley, 2006). Quality of life will be assessed by the EQ-5D-5L (http://www.euroqol.org/); suicide ideation will be assessed by the Columbia-Suicide Severity Rating Scale (Posner et al., 2011); overall paranoia will be assessed by the Revised-GPTS (Green et al., 2008; Freeman et al., 2019)<br><br>3. Delusion severity will be assessed by PSYRATS (Haddock et al, 1999).) Wellbeing will be assessed by the WEMWBS (Tennant et al., 2007), perceptions of recovery will be assessed by the QPR (Neil et al., 2009).<br><br>4. As in 1-3 above	1. 0 and 4 weeks.<br><br>2. 0, 4, and 24 weeks (primary end point 4 weeks)<br><br>3. 0, 4, and 24 weeks (primary end point 4 weeks)<br><br>4. 8, 16, and 24 weeks
**Tertiary Hypothesis:**<br><br>Change in delusion conviction will be mediated by changes in safety beliefs and use of defense behaviors.	Use of defence behaviours will be assessed using the Safety Behaviours Questionnaire (Freeman et al., 2001; strength of safety beliefs will be assessed using a visual analogue scale (Freeman et al., 2016).	0, 2, 4, 8, 16, and 24 weeks<br><br>(primary end point 4 weeks)

© Copyright: The University of Oxford and Oxford University Hospitals NHS Foundation Trust 2016

6. TRIAL DESIGN

The design is a parallel group randomised controlled trial with single blind assessment to test whether the new psychological treatment (VRCB) will reduce persecutory delusions more effectively than VR mental relaxation (VRMR; a control condition, controlling for time in VR). Standard care will be measured (CSRI; Beecham and Knapp, 1992) but remain as usual in both groups. Assessments will be carried out at 0, 2, 4 (post treatment) and 8, 16, and 24 weeks by a researcher blind to treatment allocation. See Trial flow diagram below. The trial will be registered with the ISRCTN and the protocol submitted for publication.

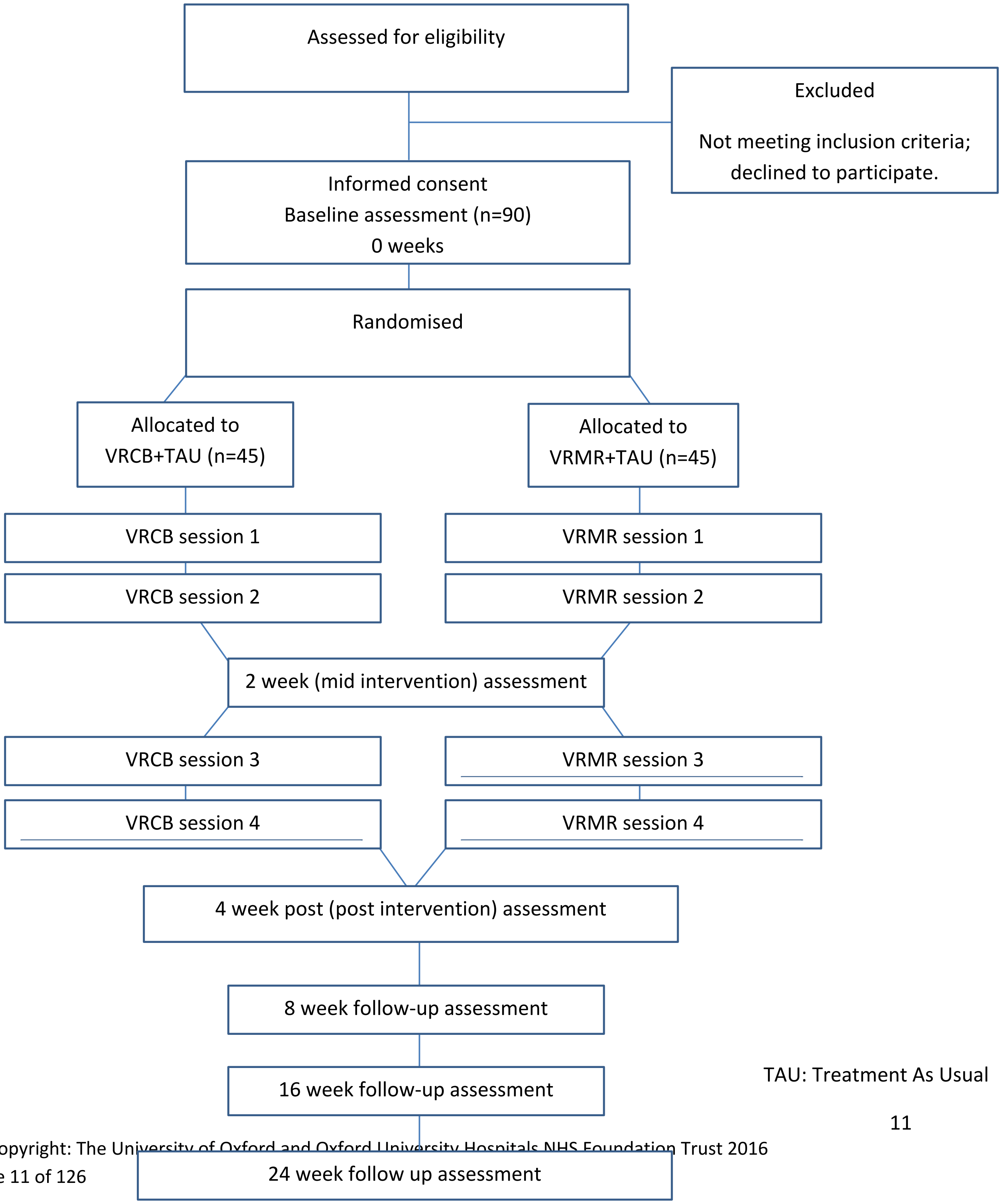

© Copyright: The University of Oxford and Oxford University Hospitals NHS Foundation Trust 2016

7. PARTICIPANT IDENTIFICATION

7.1. Trial Participants

Participants with persistent persecutory delusions in the context of non-affective psychosis.

7.2. Inclusion Criteria

- Participant is willing and able to give informed consent for participation in the trial.
- Male or Female, aged 16 years or above.
- Persistent (at least 3 months) persecutory delusion (as defined by Freeman & Garety, 2000), held with at least 50% conviction; specifically, participants will be reporting feeling threatened when with other people.
- Primary diagnosis of schizophrenia-spectrum psychosis (non-affective psychosis).

7.3. Exclusion Criteria

The participant may not enter the trial if ANY of the following apply:

- Primary diagnosis of alcohol or substance disorder
- Photosensitive epilepsy
- Significant visual, auditory, or balance impairment
- Current receipt of another psychological therapy
- Insufficient comprehension of English
- In forensic settings
- Organic syndrome
- Learning disability
- Current active suicidal plans

A participant may also not enter the trial if there is another factor, which, in the judgement of the investigator, would preclude the participant from providing informed consent or from safely engaging with the trial procedures. Reason for exclusion will be recorded in line with CONSORT guidelines.

8. TRIAL PROCEDURES

The schedule of procedures is summarised in appendix A.

© Copyright: The University of Oxford and Oxford University Hospitals NHS Foundation Trust 2016

For all trial home visits, the lone worker Standard Operating Procedure (SOP) should be followed. For further information on recruitment and assessment, please see the relevant SOP.

8.1. Recruitment

Referrals to the trial will be sought from the relevant clinical teams in the mental health Trusts. If a patient indicates to their clinical care team that they are willing to be approached by the research team, then information about the trial will be provided and screening conducted. All suitable patients will be given at least 24hours to consider taking part in the trial, although in practice it is typically a week. Recruitment will be from local NHS Foundation Trust sites.

Screening and Eligibility Assessment

The key screening with the patient is for the presence of a current persecutory delusion; specifically, that they report feeling threatened when with other people. This is established in a brief discussion with the patient. The clinical diagnosis is provided by the Trust clinical team.

8.2. Informed Consent

Written and verbal versions of the Participant Information and Informed Consent will be presented to the participants detailing no less than: the exact nature of the trial; what it will involve for the participant; the implications and constraints of the protocol; the known side effects and any risks involved in taking part. It will be clearly stated that the participant is free to withdraw from the trial at any time for any reason without prejudice to future care, without affecting their legal rights and with no obligation to give the reason for withdrawal. Both Participant Information and Informed Consent documents have been reviewed by our Patient Advisory Group (organised by the McPin Foundation).

The participant will be allowed as much time as wished to consider the information, and the opportunity to question the Investigator, their mental health team or other independent parties to decide whether they will participate in the trial. Written Informed Consent will then be obtained by means of participant dated signature and dated signature of the person who presented and obtained the Informed Consent. We will also accept written informed consent electronically. Oral consent can also be taken over the phone using the oral consent form, which will be signed and dated by the person who obtained the Informed Consent orally. The person who obtained the consent must be suitably qualified and experienced, and have been authorised to do so by the Chief Investigator. A copy of the signed Informed Consent will be given to the participant. If collecting orally, the signed consent form will be sent to the participant via secure post or password-protected email. The original signed form will be retained at the trial site and uploaded to the participant's clinical notes.

8.3. Randomisation, blinding and code-breaking

Randomisation will occur after completion of the baseline assessment. Allocation to VRCB and VRMR will be 1:1. Randomisation will be carried out by an online system designed by the University of Oxford Primary Care Clinical Trials Unit. Randomisation using a permuted blocks algorithm, with randomly varying block size, will be stratified by severity of delusion (moderate (50-75% conviction)/high (76%+ conviction)).

The trial assessors will be blind to group allocation, but the patients and trial therapists will not be (they cannot be blinded to what psychological treatment is delivered or received). The trial therapists will inform

© Copyright: The University of Oxford and Oxford University Hospitals NHS Foundation Trust 2016

patients of the randomisation outcome, so that the research assessors remain blind to group allocation. Precautionary strategies to prevent breaks of blind include: the therapist and assessor considering room use and booking arrangements; patients being reminded by the assessor not to talk about treatment allocation; and, after the initial assessment, the assessor not looking at the patient's clinical notes. If an allocation is revealed between assessment sessions, this is logged by the trial coordinator/PI and re-blinding will occur using another assessor.

8.4. Baseline Assessments

The measures have been successfully used in the previous pilot study and other studies within the research team. Assessments are in person, typically in clinic rooms or at home (for patients who find it difficult to leave their residence) and at least 24 hours after participant screening, or remotely. Basic demographic and clinical data will be collected (e.g. age, gender, ethnicity, clinical diagnosis). The primary outcome measure will be conviction in the persecutory delusion (using a 0–100% scale). As in the previous pilot, a behavioural test will assess distress in real situations (O-BAT; Freeman et al., 2016). Activity levels will be assessed using actigraphy (over 7 days), complemented with a time-budget assessing meaningful activity (Jolley, 2006). The EQ-5D-5L (http://www.euroqol.org/) will assess quality of life. Self-reported difficulties related to going into specific situations/environments will be assessed using a brief questionnaire (called the Brief Avoidance Scale [BAS]). Suicidal ideation (Columbia Scale; Posner, 2011), overall paranoia (Revised-GPTS; Green, 2008; Freeman et al., 2019), and delusion severity (PSYRATS; Haddock et al, 1999) will be assessed. Additionally, wellbeing will be assessed using the Warwick-Edinburgh Mental Wellbeing Scale (WEMWBS) (Tennant et al., 2007) and the Questionnaire about the Process of Recovery (QPR); a tool developed in collaboration with service users, will assess participant perceptions of recovery (Neil et al., 2009). For mediation, we will assess use of defence behaviours (SBQ; Freeman et al, 2001) and strength of safety beliefs (Freeman et al, 2016). Adverse events will be monitored. Additionally, we will record service use using the Client Service Receipt Inventory (CSRI; Beecham & Knapp, 1992). This will provide health economic data to support assessment of economic viability, should the VRCB treatment be shown to be clinically effective.

8.5. Subsequent Visits

There are five further trial assessments: at 2, 4, 8, 16, and 24 weeks. The main delusion measures (0-100% rating of conviction, PSYRATS, R-GPTS) and mediation measures (SBQ and strength of safety beliefs) will be completed at all assessments, with the full battery, described above in 'Baseline Assessments', completed at 4 and 24 weeks. There will be one additional questionnaire at the 8 week assessment: the Defence Behaviours Questionnaire (DBQ), which is a self-report version of the SBQ and measures defence behaviours. We are only including the DBQ at one time point in order to assess for its reliability by comparing it to the currently validated interview (SBQ). It is hoped that the DBQ will eventually replace the SBQ and therefore reduce participant burden. The behavioural test, assessing distress in real situations, will only be completed again at the 4 week assessment

Where it is not possible for the questionnaires to be administered face-to-face, they can be administered online, on the phone or via post.

Approximately four treatment sessions are offered over approximately four weeks. Any variation to this will be recorded in the CRF. These visits will be conducted at a location convenient to the participant;

© Copyright: The University of Oxford and Oxford University Hospitals NHS Foundation Trust 2016

typically, the clinic or participant's home. The virtual reality setup we use is commercially available and extensively used. Additionally, we have used VR with over 50 patients with persecutory delusions and several hundred members of the general population with no side effects. However, as there have been reports of simulator sickness in VR in the past (typically using older, less sophisticated setups than our own), we will include a simulator sickness measure at the start and end of the first treatment session. This will help us to monitor safety.

Credibility of both treatments will be assessed with the Credibility/Expectancy Questionnaire (Devilly & Borkovec, 2000) in the first treatment session, after an explanation has been given about the treatment the participant is due to receive. This is a standard measure within our team to monitor perceived credibility of interventions.

8.6. Discontinuation/Withdrawal of Participants from Trial

Withdrawal of participants from the trial assessments

Each participant has the right to withdraw from the trial at any time. Withdrawal from the trial will not result in exclusion of the previously collected data for that participant from analysis (unless this is specifically requested). The reason for withdrawal will be recorded in the CRF.

Withdrawal of participants from the trial intervention

Each participant has the right to withdraw from either trial intervention at any time. Withdrawal from the trial intervention will not result in exclusion of the previously collected assessment data for that participant from analysis (unless this is specifically requested). In addition, the Investigator may discontinue a participant from a trial intervention at any time if the Investigator considers it necessary for any reason including:

- An adverse event which requires discontinuation of the trial intervention
- Ineligibility e.g. presence of photosensitive epilepsy, which was not known to the team at screening

If the participant is withdrawn due to an adverse event, the Investigator will arrange for follow-up visits or telephone calls until the adverse event has resolved or stabilised.

The reason for withdrawal will be recorded in the CRF.

8.7. Definition of End of Trial

The end of trial is the date of the last assessment of the last participant.

8.8. Considerations Related to COVID-19

In light of the occurrence of the COVID-19 pandemic during the course of the study, some alterations have been made to study procedures to reduce the risk of potential exposure to COVID-19 by participants. Such changes will be continue to be made, as required, in line with prevailing national and local guidance (which

© Copyright: The University of Oxford and Oxford University Hospitals NHS Foundation Trust 2016

are highly likely to change over time). Changes will not be made that would create additional burden to NHS staff or resources. These changes include the following:

- The real world distress task (a secondary outcome) will not be administered because of social distancing restrictions.
- Upon restarting after the suspension of the trial due to COVID-19, we will continue the recruitment suspension into the trial for participants who have any of the conditions that would make someone high or moderate risk (clinically vulnerable) for a severe course of COVID-19 (https://www.nhs.uk/conditions/coronavirus-covid-19/people-at-higher-risk/whos-at-higher-risk-from-coronavirus/). As of February 2021, we are now allowing those who are moderate or high risk to take part if they have been vaccinated against COVID-19.
- We have added an addendum to the Participation Information Sheet to indicate the steps the research team has taken to reduce the risk of COVID-19 transmission. We will discuss this addendum with participants when we meet with them. The addendum has been reviewed by advisory group.
- We will offer flexibility for participants to conduct any of the follow-up assessments remotely, following an appropriate risk assessment.
- In addition to patient preference, we will conduct a COVID-19 risk assessment when determining where VR treatment sessions will occur. Due to COVID-19, we have enhanced our cleaning and hygiene procedures for using the VR equipment. This can be found in the VR Decontamination SOP.
- We have updated the relevant SOP with information about data confidentiality procedures during COVID-19 and remote working.

9. PSYCHOLOGICAL TREATMENTS

9.1. Description

VR Confidence Building. After the assessment, the patient will be offered 4 sessions of VR Confidence Building (VRCB). Each session, patients will be able to choose one of the four available virtual reality sub-scenarios within a coherent shopping centre environment: a café, a lift, a central area, or a clothes shop. This virtual environment represents real life situations, which patients with persecutory delusions may come across in day to day life and often find challenging. In these scenarios, a virtual coach (which speaks and moves as a real person would) will encourage the participant to enter the feared situation (which include virtual people), provide psychoeducation, and encourage them to drop safety behaviours. Participants will be also encouraged to use their experiences in the virtual environment to evaluate their threat beliefs. This will directly target key hypothesised maintenance factors for persecutory delusions. VRCB is based on the procedure used in the pilot study, however the pilot involved only one session and had no virtual coach. Participants will spend approximately 20-25 minutes in VR in each session.

VR Mental Relaxation. After the assessment, the participant will be offered 4 sessions of VR mental relaxation. Each session, participants will choose from a selection of calm virtual reality environments:

© Copyright: The University of Oxford and Oxford University Hospitals NHS Foundation Trust 2016

these may include, a beach, a countryside scene, a forest scene, and a lake. The VR environments are from a commercially available VR relaxation program which is already accessible to the general public. Participants will be given information on relaxation techniques, such as a simple breathing exercise or mindful attention to their surroundings. With VRMR it is explained that the way to deal with a fear is to be calm in your own mind. ‘Mental relaxation’ switches off our alarm systems, makes us less anxious, and creates a sense of safety. We will help patients become much better at mental relaxation, taking them to calm places in VR to practice these techniques. We’ll teach how to let any fearful thoughts float by and provide techniques to practice mental relaxation. As with VRCB, participants will spend approximately 20-25 minutes in VR in each session. To reduce the likelihood of eyestrain/discomfort while in a VR scenario in which participants are not actively engaging with the environment, participants receiving this treatment will be able to swap to a second scenario after approximately 10 minutes.

9.2 Device details

Description of device

Virtual Reality Confidence Building (VRCB) is a virtual-reality application recommended for adults (16+) with a diagnosis of non-affective psychosis, who feel under threat when they are around other people. This software is intended to reduce concerns about harm from others and therefore to help participants feel safer and more comfortable around people.

Manufacturer details

The University of Oxford is the manufacturer for this device.

Details of software

The VRCB software application is composed of a set of virtual environments, including different scenes created using 3D models, ambient audio, and 3D computer characters, with animations and speech. The environments are driven by source code which handles the logic of the program, the behavior of the computer characters, as well as the user interaction and data storage. The code is implemented on top of Universitat de Barcelona background libraries and third-party libraries. The software is built using Unity (Unity Technologies©). Unity acts as a render engine, displaying the virtual environments to the user through the headset.

Use of device

Detailed guidance on use is included within the application; participants will have a short tutorial showing them how to interact with the virtual environment and advising them of safety precautions. The application will be run and monitored by a qualified clinician, who will remain present throughout the session.

Details of accessories required

The application will run through the Steam® software application on a laptop computer connected to the HTC Vive™ headset and accessories (two handheld controllers and two ‘lighthouse’ sensors, set up in

© Copyright: The University of Oxford and Oxford University Hospitals NHS Foundation Trust 2016

corners of the room). All technical requirements will be as per Vive requirements. Accessory hardware and software are already commercially available and have not been modified for the trial.

Anonymised data collected during the session (e.g. participant number, time in VR, difficulty level reached) will be automatically stored by the application on the password protected University of Oxford laptop computer hard disk.

Handling, maintenance, storage & accountability

Physical accessories will be maintained as per manufacturer's instructions. The Vive headset will use a wipeable insert, and both this and the handheld controllers will be cleaned after each use, using recommended hygienic wipes, by the clinician present during the session. Participants will not come into direct contact with the laptop computer or 'lighthouses'.

The clinician will check that the laptop computer has the most up to date version of the software before each session, and if necessary, download this to the computer prior to the session.

Labelling

The software will contain appropriate labels, such as the version number and manufacturer. The hardware will be labelled for traceability.

Contraindications

Contraindications for use include:

- Photosensitive epilepsy
- Significant auditory or visual impairment
- Insufficient comprehension of English

For full details, see 'Exclusions' section. All participants will be screened with their clinical team prior to entry to the study.

Precautionary measures for non-CE marked device use

A qualified clinician will set up the hardware for the use of the device and while the device is in use, a qualified clinician will be present at all times. Participants will only be entered into the study with the permission of their clinical teams and following screening to ensure that no contraindications to use are present. Adverse events will be monitored and simulator sickness assessed in the first session.

Further details on the device may be found in the device specification.

9.3 Compliance with Trial Interventions

Participants will be supported to engage in both interventions by either research team clinical psychologists (with honorary Trust clinical contracts) or other mental health staff, under supervision of the trial team. For both interventions, the number of sessions and time in VR will be recorded. As the VR interventions use preprogramed virtual environments and written relaxation techniques, possible deviation from intervention protocols should be minimal. Patient beliefs about the potential effectiveness

© Copyright: The University of Oxford and Oxford University Hospitals NHS Foundation Trust 2016

of the intervention that he or she will receive will be assessed after information on the intervention is provided at the start of the first session, with the Credibility/expectancy questionnaire (Devilly & Borkovec, 2000).

10 SAFETY REPORTING

Adverse events are rare in our studies, even though this patient group has a higher rate than the general population for the occurrence of adverse events. For example, suicide attempts occur at a higher rate, as do physical health problems. In our Worry Intervention Trial with 150 patients with persistent persecutory delusions followed for six months, no patients died or were admitted to secure units during the study but there were six suicide attempts (two in the psychological treatment intervention group, and four in the standard care control group), and two serious violent incidents (one in each group). None were deemed by the DMEC to be related to the trial. Serious adverse events related to psychological reactions (i.e. SAR or SUSAR) are extremely rare (and have not occurred in our studies).

In order to monitor for adverse events, we maintain close links with the participant's clinical team throughout, have a qualified clinician present throughout while the device/VR equipment is in use, and record events that we become aware of during a participant's participation. For the full list of adverse events we record, see the SOP. We also check medical notes at the end of a patient's participation for the following events pre-specified as adverse: 1. All deaths. 2. Suicide attempts. 3. Serious violent incidents. 4. Admissions to secure units. 5. Formal complaints about therapy.

If significant concerns about possible future risk to self or others are raised in assessment or intervention sessions, we risk assess the nature, severity, and likelihood of the risk. This is discussed with a team clinical psychologist (if not already present) and the patient's clinical team is informed as a matter of urgency.

10.1 Definitions of Adverse Events

Any untoward medical occurrence, unintended disease or injury, or untoward clinical signs in participants, whether or not related to the investigational medical device (i.e. VRCB). This includes adverse events related to the VRCB intervention group and to the control group (VR mental relaxation) and also to all research procedures involved.

We note that a temporary increase in anxiety symptoms is expected in any psychological treatment involving confronting a feared situation and this would not be considered an adverse event.

10.2 Definitions of Serious Adverse Events

An adverse event is defined by the ISO14155:2011 guidelines for medical device trials as serious if it:

a) Results in death or,
b) Is a life-threatening illness or injury or,
c) Requires [voluntary or involuntary] hospitalisation or prolongation of existing hospitalisation or,
d) Results in persistent or significant disability or incapacity or,
e) Medical or surgical intervention required to prevent any of the above,

© Copyright: The University of Oxford and Oxford University Hospitals NHS Foundation Trust 2016

f) Leads to foetal distress, foetal death or consists of a congenital anomaly or birth defect or,
g) Is otherwise considered medically significant by the investigator.

Life threatening in the definition of an SAE refers to an event in which the subject was at risk of death at the time of the event; it does not refer to an event that hypothetically might have caused death if it were more severe. Clinical judgement should be exercised in deciding whether an SAE is serious in other situations.

Important: AE's that are not immediately life-threatening or do not result in death or hospitalisation but may jeopardise the subject or may require intervention to prevent one or the other outcomes listed, should be considered serious.

A planned hospitalization for a pre-existing condition, without a serious deterioration in health, is not considered to be a serious adverse event. We note that admissions to psychiatric hospital are expected in this client group.

To ensure no confusion or misunderstanding of the difference between the terms "serious" and "severe", which are not synonymous, the following note of clarification is provided: The term "severe" is used to describe the intensity (severity) of a specific event (as in mild, moderate, or severe myocardial infarction); the event itself, however, may be of relatively minor medical significance (such as severe headache). This is not the same as "serious," which is based on patient/event outcome or action criteria usually associated with events that pose a threat to a participant's life or functioning. Seriousness (not severity) serves as a guide for defining regulatory reporting obligations.

10.3 Adverse Device Effect (ADE)

Adverse event related to VRCB Investigational Medical Device (i.e. VRCB application). This includes adverse events resulting from insufficient or inadequate instructions for use, deployment, installation, or operation, or any malfunction of the software. It also includes any event resulting from user error or intentional misuse of VRCB.

User error refers to an act or omission of an act that results in a different device response than intended by the manufacturer or expected by the user.

10.4 Serious Adverse Device Effect (SADE)

Adverse device effect that has resulted in any of the consequences characteristic of a serious adverse event. This includes Device Deficiencies that might have led to a serious adverse event if:

a) Suitable action had not been taken or,
b) Intervention had not been made or,
c) If circumstances had been less fortunate.

© Copyright: The University of Oxford and Oxford University Hospitals NHS Foundation Trust 2016

10.5 Device Deficiencies

Inadequacy of the VRCB medical device (i.e. the VRCB application) with respect to its identity, quality, reliability, safety or performance. Device Deficiencies include malfunctions, end user errors, and inadequate labelling. Some deficiencies may have led to an adverse device effect or a serious adverse device effect, and should be treated as ADEs/SADEs.

10.6 Anticipated Serious Adverse Device Effect

A serious adverse device effect which by its nature, incidence, severity or outcome has been previously identified in the risk analysis report or the Investigator's Brochure. For VRCB we do not anticipate any Serious Adverse Device Effects.

10.7 Unanticipated Serious Adverse Device Effect

Serious adverse device effect which by its nature, incidence, severity or outcome has not been identified in the current version of the risk analysis report or the Investigator's Brochure.

10.8 Causality

The relationship between the investigational medical device or other research procedure and the occurrence of each adverse event will be assessed and categorised. The investigator will use clinical judgement to determine the relationship. Alternative causes, such as natural history of the participant's underlying condition, concomitant therapy, other risk factors etc. will be considered. The Investigator will also consult the current version of the risk analysis report and/or the investigator's brochure.

Classification	Relationship	Definition
**Related**	**Definitely**	• Starts within a time related to the study device/procedure *and*<br>• No obvious alternative medical explanation.
	**Probably**	• Starts within a time related to the study device/procedure *and*<br>• Cannot be reasonably explained by known characteristics of the patient's clinical state.
	**Possibly**	• Starts within a time related to the study device/procedure *and*<br>• A causal relationship between the intervention and the adverse event is at least a reasonable possibility.
**Not related**	**Probably not**	• The time association or the patient's clinical state is such that the study device/procedure is not likely to have had an association with the observed effect.
	**Definitely not**	• The AE is definitely not associated with the study device/procedure.

10.9 Reporting Procedures for Adverse Events

© Copyright: The University of Oxford and Oxford University Hospitals NHS Foundation Trust 2016

Adverse Events are reportable from the time of study enrolment. Study enrolment is defined as the time at which, after recruitment, the participant has signed and dated the informed consent form.

See relevant SOP for full details of reporting procedures.

Reporting to the MHRA

All serious adverse events that come to our attention are reviewed by the study team. These include serious events which are:

a) Related to the device and those which are non-device related;
b) Anticipated and unanticipated serious events;
c) Device Deficiencies that might have led to a serious adverse event if:
    - Suitable action had not been taken or, intervention had not been made or,
    - If circumstances had been less fortunate.

SAEs are recorded using the Serious Adverse Event Report Form. There is a legal requirement for a manufacturer to report all serious events to the Medicines Healthcare Regulatory Authority (MHRA) immediately (without any unjustifiable delay) within the timescales set out below. In this case, the study team will make an initial assessment of whether the SAE is potentially related to the device and report to the MHRA within the appropriate timescales. The decision about relatedness will later be ratified by the DMEC.

Timescales:

a) SAEs that indicate the death of a participant, an imminent risk of death, serious injury, or serious illness that requires prompt remedial action for other participants, users or other persons, or a new finding relating to a previously reported SAE of this seriousness must be reported to the MHRA no more than 2 calendar days following awareness of the event by the study team.
b) Other reportable events not described above or follow up information relating to those events must be reported to the MHRA within 7 days of the study team becoming aware of the event.

Where the Chief Investigator is not available to sign the SADE form, the study team should not delay in sending the SADE report. A copy of the original submitted form signed by the CI must be forwarded as soon as possible.

Events will be followed up until resolution, any appropriate further information will be sent by the research team in a timely manner.

© Copyright: The University of Oxford and Oxford University Hospitals NHS Foundation Trust 2016

Reporting to the DMEC

For all Serious Adverse Events, the study Chief Investigator or study team member will notify the Chair of the Data Monitoring and Ethics Committee for a decision on whether it is potentially related to the device (VRCB application), and/or trial procedures more generally. Any initial decision submitted to the MHRA on relatedness to the device, will be ratified by the DMEC.

Reporting to the Research Ethics Committee

A serious adverse event (SAE) occurring to a participant will be reported to the REC that gave a favourable opinion of the study where the event was 'related' (resulted from administration of any of the research procedures) and 'unexpected' in relation to those procedures. Reports of related and unexpected SAEs will be submitted within 15 working days of the Chief Investigator becoming aware of the event, using the HRA report of serious adverse event form (see HRA website).

Device deficiencies

Device deficiencies, which are not considered to be contributory toward SADEs shall be noted by any member of the research team using the Device Deficiency Report.

If the device deficiency led to an SAE or may have led to an SAE had circumstances not been different then this will be reported to the MHRA as per the reporting requirements described above.

Any new device deficiencies identified, which were not considered in the initial risk analysis, will be added to the risk analysis and to the Investigators Brochure.

10.10 Safety Monitoring Committee

We will form a Data Monitoring and Ethics Committee (DMEC) with an independent clinician chair, independent statistician, and further independent clinician. The data and management of Adverse Events will be overseen by the chair of the Data Monitoring and Ethics Committee (DMEC).

11 STATISTICS

A full statistical analysis plan will be written by the trial statistician (LMY) prior to any analysis being undertaken. We will report data in line with the Consolidated Standards of Reporting Trials (CONSORT) 2010 Statement showing attrition rates and loss to follow-up. All analyses will be carried out using the intention to treat principle with data from all participants included in the analysis including those who do not complete therapy. Every effort will be made to follow up all participants in both arms for research assessments.

Analysis will be conducted in Stata. $P<0.05$ will be used as the level of statistical significance. Descriptive statistics within each randomised group will be presented for baseline values. These will include counts and percentages for binary and categorical variables and means and standard deviations, or medians with

© Copyright: The University of Oxford and Oxford University Hospitals NHS Foundation Trust 2016

lower and upper quartiles, for continuous variables, along with minimum and maximum values and counts of missing values. There will be no tests of statistical significance or confidence intervals for differences between randomised groups on any baseline variable.

Power and the interim analysis

Based on the pilot test against exposure (Freeman et al, 2016), we expect at least a VRCB 20% reduction in delusional conviction (effect size=1.0) compared to VRMR. This is a conservatively lower effect size expectation than the first pilot, despite the first pilot using a briefer VRCB and a control condition that included one part of active treatment. Nonetheless, we recognise that an even lower effect size (d=0.75, reflecting a 15% reduction in conviction) for delusions would still be of interest to pursue and thus we power the full trial (n=90) on this basis. A trial with 45 participants in each arm (allowing up to 15% loss to follow-up) will have approximately 90% power to detect a statistically-significant treatment effect at 4 weeks, using an independent groups t-test and a significance level of 0.05, if the true standardised effect size is 0.75. Following guidelines for good practice (Lancaster et al, 2004), interim analysis will provide simple descriptive statistics and an initial estimate of the 95% confidence interval for the treatment effect. This interim analysis of the week 4 data after 30 participants, will provide an estimate of conditional power (i.e. power given the data obtained so far - Whitehead & Matsushita, 2003; Snapinn et al., 2006).

We will stop the trial if the interim estimate of effect size, d, is 0.1 or lower, implying that the conditional power of the full trial, based on the interim results and the hypothesised effect size of 0.75, would be 60% or lower. If it were assumed that the treatment effect seen in the pilot would continue throughout the rest of the trial, then the conditional power would be as low as 3%.

Hypothesis testing

The primary hypothesis is for change in the primary outcome measure, conviction in the persecutory delusions (using a 0-100% scale) at 4 weeks. Additionally, repeated measures are also assessed at the 4 week point and again at 8, 16, and 24 weeks. Random or mixed effects models (using Stata's xtreg command) will be fitted to the repeated measures to estimate treatment effects. The mixed effect models will include the outcome as the response variable, time point, randomised group, and baseline score as fixed effects and a patient specific random intercept. An interaction between time and randomised group will be fitted as a fixed effect to allow estimation of treatment effect at all time points.

The mediation analysis will investigate putative mediational factors (safety behaviours and safety beliefs) using modern causal inference methods (Dunn et al, 2015). This involves using parametric regression models to test for mediation of VRCB on outcome through the putative mediators. Analyses will adjust for baseline measures of the mediator, outcomes, and possible measured confounders. We will include repeated measurement of mediators and outcomes to account for classical measurement error and baseline confounding.

Missing data

Missing data on individual measures will be pro-rated if more than 90% of the items are completed; otherwise the measure will be considered as missing.

12 DATA MANAGEMENT

© Copyright: The University of Oxford and Oxford University Hospitals NHS Foundation Trust 2016

12.1 Source Data

We keep data from the assessments, collected on paper from the participant interviews. Additionally, belief ratings made within the VR environment will be collected. All documents, including electronic files, will be stored safely in confidential conditions. On all trial-specific documents, other than the signed consent, the participant will be referred to by the trial participant number, not by name.

12.2 Access to Data

Direct access will be granted to authorised representatives from the Sponsor, host institution and the regulatory authorities to permit trial-related monitoring, audits and inspections.

12.3 Data Recording and Record Keeping

All trial data will be entered on to the statistical analysis programme SPSS. The participants will be identified by a unique trial specific number in databases. The name and any other identifying detail will not be included in any trial data electronic file. Source data will be stored in a locked cabinet in a locked room for ten years post publication of the trial results.

13 QUALITY ASSURANCE PROCEDURES

The trial will be conducted in accordance with the current approved protocol, GCP, relevant regulations and standard operating procedures. We employ a regulatory consultant to advise us on compliance with medical device specific regulations. Data will be evaluated for compliance with the protocol and accuracy in relation to source documents. All electronic data entry is double checked against the source documents. Additionally, following transfer of the complete dataset to the statistician, the main outcomes will be checked for accuracy. A DMEC will meet before the start of the trial, with the subsequent meeting frequency to be agreed by committee members. The committee will be chaired by a local clinician with relevant expertise.

14 ETHICAL AND REGULATORY CONSIDERATIONS

14.1 Declaration of Helsinki

The Investigator will ensure that this trial is conducted in accordance with the principles of the Declaration of Helsinki.

14.2 Guidelines for Good Clinical Practice

The Investigator will ensure that this trial is conducted in accordance with relevant regulations and with Good Clinical Practice.

14.3 Approvals

© Copyright: The University of Oxford and Oxford University Hospitals NHS Foundation Trust 2016

The protocol, informed consent form, participant information sheet and any proposed advertising material will be submitted to an appropriate Research Ethics Committee (REC), HRA (where required) and host institution(s) for written approval.

The Investigator will submit and, where necessary, obtain approval from the above parties for all substantial amendments to the original approved documents.

Appropriate regulatory approval will be sought for the use of the device (VRCB) within the trial from the MHRA, to comply with medical device legislation, prior to the trial.

14.4 Reporting

The CI shall submit, on request, an Annual Progress Report to the REC, HRA (where required), host organisation and Sponsor. In addition, an End of Trial notification and final report will be submitted to the the REC, host organisation and Sponsor.

14.5 Participant Confidentiality

The trial staff will ensure that the participants' anonymity is maintained. The participants will be identified only by a participant ID number on all trial documents and any electronic database, with the exception of the CRF, where participant initials may be added. All documents will be stored securely and only accessible by trial staff and authorised personnel. The trial will comply with the Data Protection Act, which requires data to be anonymised as soon as it is practical to do so.

14.6 Expenses and Benefits

For each trial assessment time point (i.e. six times), patients will be reimbursed £10 for their time and effort. Reasonable travel expenses for any visits additional to normal care will be reimbursed on production of receipts, or a mileage allowance provided as appropriate.

14.7 Ethical Considerations

We anticipate few ethical concerns for patients entering this study. Participation in the trial does not change existing treatment receipt. We will, however, be asking half of the patients to enter the types of environment that make them anxious and in which they have paranoid thoughts (e.g. going to a café). However, these are everyday environments that they are encountering routinely. We are simply studying responses to them, and in some encouraging potentially more adaptive responses. Moreover, we will be picking situations with the participants that they would like to feel more confident and safer within. We have successfully carried this out in previous studies (without the therapeutic technique of reducing safety behaviours). The VR environments will feel safer because the patient will know they are computer generated. Moreover, based on the previous pilot data, half of the participants are likely to show clinical benefits from taking part, for example feeling less concerned about entering such environments in the immediate future.

There is clinical equipoise between the two psychological treatments. Both may have benefits for patients. However, it is hypothesised that gains will be greater with VRCB. Clinical equipoise exists because VRCB has not been evaluated in a randomised controlled trial against the proposed control condition (VRMR) and collective professional opinion would be that such an evaluation is needed to determine its efficacy.

© Copyright: The University of Oxford and Oxford University Hospitals NHS Foundation Trust 2016

Should the VRCB treatment be shown to be more effective than the control, a separate trial will further develop the treatment and assess the most appropriate way to make the treatment available to patients going forward.

The other main ethical issue is the burden of the assessments for the participants. These typically take around one hour. However, we have successfully used these assessments before (indeed have used much longer assessments in trials). It is generally a patient group who have limited social contact, who often have few activities during the day, and who appreciate the time spent with our staff. Hence in our clinical trials there is always improvement in the control condition even when that just comprises the additional monitoring. Patients can take breaks and also complete the assessments over several meetings. Nevertheless, if a patient does find the assessments too long then the battery can always be shortened to the primary measure. However, our data completion rates are typically very high, as are our follow-up rates, indicating that patients are fully informed about what the trial will involve.

14.8 Other Considerations

Safety of researchers is very important; therefore, we follow a standard operating procedure for lone working.

For details on safety of the equipment, see section 9.2 above.

As noted above, a potential conflict of interest exists due to the CI and Sponsor's involvement in a Spin out company, to which VRCB could be licensed in future. There is a conflict of interest plan in place in the Department of Psychiatry.

15 FINANCE AND INSURANCE

15.1 Funding

The trial is funded by the Medical Research Council (MRC) via the Developmental Pathway Funding Scheme (DPFS).

15.2 Insurance

The University has a specialist insurance policy in place which would operate in the event of any participant suffering harm as a result of their involvement in the research (Newline Underwriting Management Ltd, at Lloyd's of London). NHS indemnity operates in respect of the clinical treatment that is provided.

16 PUBLICATION POLICY

The results of the trial will be published in a journal. All investigators would be expected to be co-authors.

17 REFERENCES

Castle, DJ., Phelan, M., Wessely, S., and Murray, RM. (1994). Which patients with non-affective functional psychosis are not admitted at first psychiatric contact? British Journal of Psychiatry, 165, 101–06.

© Copyright: The University of Oxford and Oxford University Hospitals NHS Foundation Trust 2016

Cooper et al. (2014). Report of the second round of the National Audit of Schizophrenia (NAS2). Royal College of Psychiatrists.

Devilly, G. & Borkovec, T. (2000). Psychometric properties of the credibility/expectancy questionnaire. Journal of Behavior Therapy and Experimental Psychiatry, 31, 73-86.

Freeman, Dunn et al (2015) Effects of cognitive behaviour therapy for worry on persecutory delusions in patients with psychosis (WIT). Lancet Psychiatry, 2, 305-313.

Freeman, D., Bradley, J., Antley, A., Bourke, E., DeWeever, N., Evans, N., Černis, E., Sheaves, B., Waite, F., Dunn, G., Slater, M., & Clark, D. (2016). Virtual reality in the treatment of persecutory delusions. British Journal of Psychiatry, 209, 62-67.

Freeman, D., Loe, B. S., Kingdon, D., Startup, H., Molodynski, A., Rosebrock, L., Brown, P., Sheaves, B., Waite, F., & Bird, J. C. (2019). The revised Green *et al.* Paranoid Thoughts Scale (R-GPTS): psychometric properties, severity ranges, and clinical cut-offs. *Psychological Medicine*, 1-10.

Freeman, D. (2016). Persecutory delusions: a cognitive perspective on understanding and treatment. Lancet Psychiatry, 3, 685-692.

Garcia-Palacios et al (2007). Comparing acceptance and refusal rates of virtual reality exposure vs. in vivo exposure by patients with specific phobias. Cyberpsychology Behavior, 10, 722-724.

Green et al (2008). Measuring ideas of persecution and reference. Psychological Medicine, 38, 101-111.

Haddock, G., McCarron, J., Tarrier, N. & Faragher, F. B. (1999). Scales to measure dimensions of hallucinations and delusions: the psychotic symptom rating scales (PSYRATS). Psychological Medicine, 29, 879-889.

Hjorthøj, C., Stürup, A E., McGrath, J J., and Nordentoft, M. (2017). Years of potential life lost and life expectancy in schizophrenia: a systematic review and meta-analysis. Lancet Psychiatry, 4, 295-301.

Hor, K. and Taylor, M. (2010). Suicide and schizophrenia: a systematic review of rates and risk factors. Journal of Psychopharmacology, 24 (11), suppl. 4, 81-90.

Jolley, S., Garety, P.A., Ellett, L., Kuipers, E., Freeman, D., Bebbington, P.E., Fowler, D.G., & Dunn, G. (2006). A validation of a new measure of activity in psychosis. Schizophrenia Research, 85, 288-295.

Kennedy, J. L., Altar, C. A., Taylor, D. L., Degtiar, I., and Hornberger, J. C. (2014). The social and economic burden of treatment-resistant schizophrenia: a systematic literature review. International Clinical Psychopharmacology, 29 (2), 63-76.

Morina, N., Iintema, H., Meyerbröker, K., & Emmelkamp, P. (2015). Can virtual reality exposure therapy gains be generalized to real-life? A meta-analysis of studies applying behavioral assessments. Behaviour Research Therapy, 74, 18-24.

Neil, S., Kilbride, M., Pitt, L., Nothard, S., Welford, M., Sellwood, W., Morrison, A. (2009). The questionnaire about the process of recovery (QPR): A measurement tool developed in collaboration with service users. Psychosis, Psychological, Social and Integrative Approaches, 1, 145-155.

Opris et al (2012). Virtual reality exposure therapy in anxiety disorders. Depression Anxiety, 29, 85-93.

© Copyright: The University of Oxford and Oxford University Hospitals NHS Foundation Trust 2016

Posner, K., Brown, G., Stanley, B., Brent, D., Yershova, K., Oquendo, M., Currier, G., Melvin, G., Greenhill, L., Shen, S., & Mann, J. (2011). The Columbia-Suicide Severity Rating Scale. American Journal of Psychiatry, 168, 1266-1277.

Snapinn, S., Chen, M.-G., Jiang, Q. & Koutsoukos, T. (2006). Assessment of futility in clinical trials. Pharmaceutical Statistics, 5, 273-281.

Tennant, R., Hiller, L., Fishwick, R., Platt, S., Joseph, S., Weich, S. et al. (2007). The Warwick-Edinburgh Mental Well-being Scale (WEMWBS): development and UK validation. Health and Quality of Life Outcomes, 5, 63-80.

Tully, S., Wells, A., and Morrison, A. P. (2016). An exploration of the relationship between use of safety-seeking behaviours and psychosis: a systematic review and meta-analysis. Clinical Psychology and Psychotherapy, 1-22.

van der Gaag, M., Valmaggia, L, R., and Smit, F. (2014). The effects of individually tailored formulation-based cognitive behavioural therapy in auditory hallucinations and delusions: A meta-analysis. Schizophrenia Research, 156, 30-37.

Whitehead, J. & Matsushita, T. (2003). Stopping clinical trials because of treatment ineffectiveness: a comparison of a futility design with a method of stochastic curtailment. Statistics in Medicine, 22, 677-687.

Vancampfort, D., Correll, C. U., Scheewe, T. W., Probst, M., De Herdt, A., Knapen, J., and De Hert, M. (2012). Progressive muscle relaxation in persons with schizophrenia: a systematic review of randomised controlled trials. Clinical Rehabilitation, 27 (4), 291-298.

Kavak, F., Unal, S., and Yilmaz, E. (2016). Effects of relaxation exercises and music therapy on psychological symptoms and depression levels of patients with schizophrenia. Archives of Psychiatric Nursing, 30, 508-512.

© Copyright: The University of Oxford and Oxford University Hospitals NHS Foundation Trust 2016

18 APPENDIX A: SCHEDULE OF PROCEDURES

Procedures	Screening	Baseline (0 weeks)	VR session 1	VR session 2	Mid intervention (2 weeks)	VR session 3	VR session 4	Post intervention (4 weeks)	Follow-up (8 weeks)	Follow-up (16 weeks)	Follow-up (24 weeks)
Eligibility assessment	X	X (brief check)									
Informed consent		X									
Demographics		X									
Brief Avoidance Scale (BAS)		X									
Randomisation		X									
Delusion conviction (0-100%)		X			X			X	X	X	X
PSYRATS – delusion		X			X			X	X	X	X
R-GPTS		X			X			X	X	X	X
Real world delusion related distress (O-BAT)		X						X			
Activity (actigraphy, time-budget)		X						X			X
EQ-5D-5L		X						X			X
Columbia scale		X						X			X
Mediators (SBQ, safety beliefs)		X			X			X	X	X	X
Self-reported defence behaviours (DBQ)									X		
WEMWBS		X						X			X
QPR		X						X			X
Service receipt (CSRI)		X									X
VR simulator sickness questionnaire			X X								
Credibility/expectancy measure			X								
VRCB or VRMR intervention			X	X		X	X				

In addition to close contact with clinical teams and reporting of adverse events when they are brought to the attention of the team as required, medical notes will be checked at the end of the trial to assess for adverse events and check service receipt.

19 APPENDIX B: AMENDMENT HISTORY

Amendment No.	Protocol Version No.	Date issued	Author(s) of changes	Details of Changes made
1	2.0	30.1.2019	Professor Daniel Freeman (chief investigator), Laina Rosebrock (trial coordinator), Cassie Hazell (postdoctoral research coordinator)	We are adding one brief questionnaire (to be completed at the baseline assessment only). This questionnaire is a self-report version of current outcome measures and will take less than 2 minutes to complete. We are adding this questionnaire in order to collect additional helpful information regarding the potential benefits of treatment. The questionnaire assesses the level of anxiety participants feel about going into several specific situations/environments and is called the Brief Avoidance Scale (BAS).
2	3.0	15.03.2019	Professor Daniel Freeman (chief investigator), Laina Rosebrock (trial coordinator)	We are removing the CHOICE questionnaire, which measures user-led outcomes, from the baseline, 4 week, and 24 week assessments. We are proposing to remove this questionnaire because similar outcomes are being measured by other questionnaires in the study and we would therefore like to reduce participant burden. We are also requesting to add one questionnaire (to be completed at the 8 week assessment only). The questionnaire is a self-report version of the Safety Behaviours Questionnaire (Freeman et al., 2001) and is being introduced to collect additional helpful information regarding one of the proposed mediators (defence behaviours). It is hoped this will eventually replace the SBQ. It will take approximately 5 minutes to complete. It is called the Defence Behaviours Questionnaire (DBQ).<br><br>We have also included the specific time period for reportable Adverse Events (AE) to make it clearer within the protocol, as currently it directs readers to the SOP.

				Finally, we have updated the personnel information in the protocol, as we have a new trial coordinator and a new trial statistician.
Non Substantial Amendment 6	3.1	17.03.2020	Professor Daniel Freeman (chief investigator), Laina Rosebrock (trial coordinator)	We are including additional information about the administration of questionnaires for follow-up assessments (2 week, 4 week, 8 week, 16 week, and 24 week) where it is not possible or appropriate to meet patients face-to-face (e.g., online or via phone or post).
Non Substantial Amendment 7	4.0	22.07.2020	Professor Daniel Freeman (chief investigator), Laina Rosebrock (trial coordinator)	Changes for restarting the trial after a suspension due to COVID-19:<br>We will be continuing the recruitment suspension into the trial for those who fall into the high/moderate risk (clinically vulnerable) group for a severe course of COVID-19.<br>We have added an addendum to our Participant Information Sheet to include information about steps we have taken to reduce the risk of COVID-19 transmission.<br>We will no longer be administering the real-world behavioural task due to COVID-19 social distancing restrictions.<br>Reference to our enhanced hygiene procedures for the VR equipment and data confidentiality procedures whilst working remotely.<br>We will continue to offer remote assessments.
Non Substantial Amendment 9	4.1	11.09.2020	Professor Daniel Freeman (chief investigator), Laina Rosebrock (trial coordinator)	In line with changes we have made due to COVID-19 to conduct assessments and screening procedures remotely, we have also changed our procedures to allow for collection of informed consent orally. This will involve the use of an oral consent form.
Non Substantial Amendment 12	4.2	17.09.2020	Professor Daniel Freeman (chief investigator), Laina Rosebrock (trial coordinator)	We have updated the informed consent procedures to indicate that we will accept written informed consent electronically, in line with changes made due to COVID-19 (see above non substantial amendments 6-9).

Non Substantial Amendment 17	4.3	28.04.2021	Professor Daniel Freeman (chief investigator), Laina Rosebrock (trial coordinator)	We have updated two of the outcome measures (R-GPTS instead of GPTS for paranoia, and indicating the real-world distress task is called the O-BAT). We have also included an additional line that as of February 2021, we have been able to recruit participants who are high/moderate risk for a severe course of COVID-19 if they have been vaccinated.

Protocol amendments must be submitted to the Sponsor for approval prior to submission to the REC committee, Health Research Authority and/or MHRA.

Primary Care
Clinical Trials Unit

STATISTICAL

The THRIVE study: A randomized controlled trial comparing Virtual Reality Confidence Building with VR Mental Relaxation for people with fears about others.

Version 3.1 22nd September 2021

	NAME	TITLE	Signature	Date
Written by:	Ly-Mee Yu (ver 1.0)	Lead Trial Statistician		
Written by:	Victoria Harris (ver 1.1 onwards)	Trial Statistician	[signature]	14/09/2021
Reviewed by:	Nicola Williams	Senior Trial Statistician	[signature]	14/09/2021
Approved by:	Daniel Freeman	Chief Investigator	[signature]	15/09/2021

<u>Version History</u>

Version:	Version Date:	Changes:
0.1	3 December 2018	Original
0.2	26 July 2019	Updated
1.0	3 August 2019	First version
1.1	17 February 2020	Updated mediation analysis. Added detail to analysis of secondary outcomes.
1.2	6 October 2020	Updated mediation and added sensitivity analysis. Clarified calculation of scores for different outcome variables.
1.3	20 October 2020	Updated following comments from the trial team.
1.4	09 February 2021	Updated following comments from NW
1.5	22 February 2021	Updated following comments from trial team
1.6	23 March 2021	Updated following comments from NW

1.7	26 March 2021	Updated following comments from trial team
1.8	30 April 2021	Updated section on medication.
2.0	04 May 2021	Final version.
2.1	13 Sept 2021	Updated O-BAT scoring
3.0	15 Sept 2021	Final version.
3.1	22 Sept 2021	Updated O-BAT scoring.

Table of Contents

Table of Contents 37
1 Introduction 39
1.1 Preface 39
1.2 Purpose and scope of the plan 39
1.3 Trial overview 39
1.4 Objectives 41
2 Trial design 43
2.1 Outcomes measures 44
2.1.1 Primary outcome 44
2.1.2 Secondary outcomes 44
2.1.3 Tertiary outcomes 46
2.2 Target population 46
2.2.1 Inclusion Criteria 46
2.2.2 Exclusion Criteria 47
2.3 Sample size 47
2.4 Randomisation and blinding in the analysis stage 48
3 Analysis – General considerations 49
3.1 Descriptive statistics 49
3.2 Characteristics of participants 49
3.3 Definition of population for analysis 49
3.4 Pooling of investigational sites 49
3.5 Data Monitoring Committee And Interim Analyses 49
4 PRIMARY ANALYSIS 50
4.1 Primary outcome 50
4.2 Handling missing data 50
4.3 Handling outliers 50
4.4 Handling multi-centre/clustered data 50
4.5 Multiple comparisons and multiplicity 51
4.6 Model assumptions 51
5 SECONDARY ANALYSIS 51
5.1 Secondary outcomes 51
5.1.1 Real world distress 52
5.1.2 Actigraphy and time budget measure 57
5.1.3 Quality of life; suicide ideation and, overall paranoia 57

5.1.4 Delusional severity, wellbeing, and perceptions of recovery 57
5.1.5 Maintenance of benefits over time 57
6 TERTIARY ANALYSIS 59
6.1 Mediation analysis 59
7 SENSITIVITY ANALYSIS 60
7.1 Outliers and missingness assumptions 60
7.2 Impact of the COVID-19 Pandemic 60
7.3 Medication effects 61
7.4 Credibility Effects 61
8 SAFETY ANALYSIS 61
8.1 Adverse events 61
9 VALIDATION 61
10 CHANGES TO THE PROTOCOL OR PREVIOUS VERSIONS OF SAP 61
11 References 62
13 Appendices 64
13.1 Appendix I. Schedule of procedures 64
13.2 Appendix II. Flow diagram of trial participants 66

1 Introduction

1.1 Preface

Chief Investigator: Professor Daniel Freeman

Trial Statisticians: Ly-Mee Yu

This SAP supports protocol version 4.2 'THRIVE Trial protocol v4.2 17.09.2020_CLEAN_Signed.docx.

1.2 Purpose and scope of the plan

This document details the proposed analyses of primary and secondary objectives for the THRIVE study, funded by the Medical Research Council Developmental Pathway Funding Scheme (MRC DPFS). Subsequent analyses of a more exploratory nature will not be bound by this strategy, though they are expected to follow the broad principles laid down here. The principles are not intended to curtail exploratory analysis nor to prohibit accepted practices, but they are intended to establish the rules that will be followed, as closely as possible, when analysing and reporting the trial. All example tables included in the plan are intended to aid the presentation of data at final analysis. However, the statistician should not be bound by these tables and is free to present the results in a suitable way.

The statistical analysis plan will be available on request when the principal papers are submitted for publication in a journal. Suggestions for subsequent analyses by the journal editors or referees will be considered carefully, and carried out as far as possible in line with the principles of the analysis strategy; if reported, the source of the suggestion will be acknowledged.

Any deviations from the statistical analysis plan will be described and justified in the final report of the trial.

1.3 Trial overview

Persecutory delusions are unfounded beliefs that others are trying to harm the person (e.g. 'People know what I'm thinking and will kill me'). Approximately 220,000 people in England and Wales have a diagnosis of schizophrenia and about 70% of patients with schizophrenia have this psychotic experience. Persecutory delusions have a substantial impact for patients; they typically lead to social withdrawal and predict hospital admission (Castle et al., 1994) and even suicide (Hor & Taylor, 2010). The total annual cost to the public sector in England is over £7 billion. Life expectancy is, on average 14.5 years shorter for people with these problems (Hjorthøj et al, 2017).

Approximately half of patients do not respond adequately to the first line treatment, medication, and residual problems are very common. In a review, Kennedy et al (2014) found that 'almost 60% of patients failed to achieve response after 23 weeks on antipsychotic drug therapy.' Meta-analysis for first generation psychological treatment indicates only small effects for delusions (g=0.36; van der Gaag et al., 2014). This suggests that psychological treatment also needs improvement. Part of the problem is that schizophrenia is an umbrella term, comprising multiple independent problems. These individual psychotic experiences,

such as persecutory delusions, need separating out in treatment development. This allows key maintenance factors to be targeted. This approach has been highly successful (Freeman et al., 2015), however there is a shortage of qualified therapists to deliver face to face interventions. With 81% of NHS patients never having received Cognitive Behavioural Therapy (CBT), the 2014 National Audit of Schizophrenia calls for NHS Trusts to increase access to evidence-based psychological interventions. Therefore, more efficacious and accessible interventions are urgently required.

The chief investigator and colleagues have developed a rigorously tested theoretical model of persecutory delusions (Freeman et al., 2016). At the core of the delusion is a belief of being unsafe; this is developed in the context of genetic and environmental risk and maintained by a number of factors, including 'defence behaviours'. When patients use these defence behaviours (such as avoidance, looking for escape routes, or taking steps to decrease their visibility when out), absence of harm is attributed to defence behaviours rather than threat belief inaccuracy. Over time, these types of behaviours are associated with increased distress and threat appraisals (e.g. Tully et al., 2016). Therefore, patients need to go into feared situations and drop defence behaviours to re-learn safety. However, patients often find it too difficult to do this.

A solution is virtual reality (VR). Patients find it much easier to enter VR environments because they consciously know that it is not real. Nonetheless, VR elicits responses comparable to those in the real world (e.g. Gorini et al., 2015) and the new learning of safety transfers into everyday life (Morina et al., 2015). VR directly tackles the key underlying mechanism of persecutory delusions: unfounded threat beliefs. VR enables patients to go into the situations that they fear, drop their defences, and truly learn that the fears are unrealistic. It builds experience of safety, self-confidence, and reversal of social withdrawal.

Virtual reality has been successfully shown to treat anxiety disorders. Meta-analyses for VR exposure in the treatment of anxiety disorders, show that the effects are large (d=1.1), maintain over years, generalise to real life, and are as good as using real life exposure (Opris et al, 2012; Morina et al, 2015). It is also the most popular treatment choice when offered, preferred over real life exposure (e.g. Garcia-Palacios et al, 2007). Using VR, patients with anxiety disorders can go into feared situations and drop defences; this helps patients learn, by direct experience, that they are safe and that fears are unrealistic.

VR may be used in a similar way, to help patients with psychosis re-learn safety. In a pilot study (Freeman et al., 2016), conducted by the research team, a single session of our theoretically driven VR cognitive treatment (going into VR feared situations with dropping of defence behaviours) led to a large reduction in the delusions (d=1.3) directly compared to an alternative active treatment (VR exposure treatment). Benefits transferred to the real world; VR cognitive treatment led to a 19.6% greater reduction in distress following a real world behavioural task compared to VR exposure. The pilot study included 30 patients with persecutory delusions, using a lab-based VR treatment.

1.4 Objectives

**Objectives**	**Outcome Measures**	**Timepoint(s) of evaluation of this outcome measure**
**Primary Objective**:<br>To determine if VRCB will lead to a reduction in delusion conviction compared with VR mental relaxation.	The primary outcome measure will be conviction in the persecutory delusion (using a 0-100% scale).	0, 2, 4, 8, 16, and 24 weeks (primary endpoint is 4 weeks)
**Secondary Objectives:**<br>1. To determine if at 4 weeks VRCB leads to a reduction in distress in real world situations compared with VRMR.<br><br>2. To determine if at 4 weeks VRCB leads to an increase in activity, quality of life, and reduction in suicide ideation and overall paranoia compared with VRMR.<br><br>3. To determine if at 4 weeks VRCB, compared with VRMR, leads to improvements in delusional severity, wellbeing, and perceptions of recovery.<br><br>4. To determine if benefits of VRCB are maintained over time.	1. Real world distress related to the persecutory delusion will be assessed using a behavioural avoidance task (O-BAT). Participants create a 5 step hierarchy of real world situations and rate levels of distress (on a scale from 0-not distressed at all to 10-extremely distressed) for each step completed, resulting in scores of avoidance and distress.<br><br>2. Activity will be assessed by actigraphy and a time-budget measure (Jolley, 2006). Quality of life will be assessed by the EQ-5D-5L (http://www.euroqol.org/); suicidal ideation will be assessed by the Columbia-Suicide Severity Rating Scale (Posner et al., 2011); overall paranoia will be assess by the Revised-GPTS (Green et al., 2008; Freeman et al., 2019).<br><br>3. Delusion severity will be assessed by PSYRATS (Haddock et al., 1999). Wellbeing will be assessed by the WEMWBS (Tennant et al., 2007); perceptions of recovery will be assessed by the QPR (Neil et al., 2009).<br><br>4. As in 2-3 above	1. 0 and 4 weeks<br><br>2. 0, 4, and 24 weeks (primary endpoint is 4 weeks)<br><br>3. 0, 4, and 24 weeks (primary endpoint is 4 weeks).<br><br>4. 8, 16, and 24 weeks.

**Tertiary Objective:**<br>To determine if changes in use of defence behaviours and safety beliefs mediate change in delusion conviction.	Use of defence behaviours will be assessed using the Safety Behaviours Questionnaire (Freeman et al., 2001); strength of safety beliefs will be assessed using a visual analogue scale (Freeman et al., 2016).	0, 2, 4, 8, 16, and 24 weeks (primary endpoint is 4 weeks).

2 Trial design

The design is a parallel group randomised controlled trial with single blind assessment to test whether the new psychological treatment (VRCB) will reduce persecutory delusions more effectively than VR mental relaxation (VRMR; a control condition, controlling for time in VR). Standard care will be measured (CSRI; Beecham and Knapp, 1992) but remain as usual in both groups. Assessments will be carried out at 0, 2, 4 (post treatment) and 8, 16, and 24 weeks by a researcher blind to treatment allocation. See Trial flow diagram below. The trial will be registered with the ISRCTN and the protocol submitted for publication.

Figure 1. Trial flow diagram.

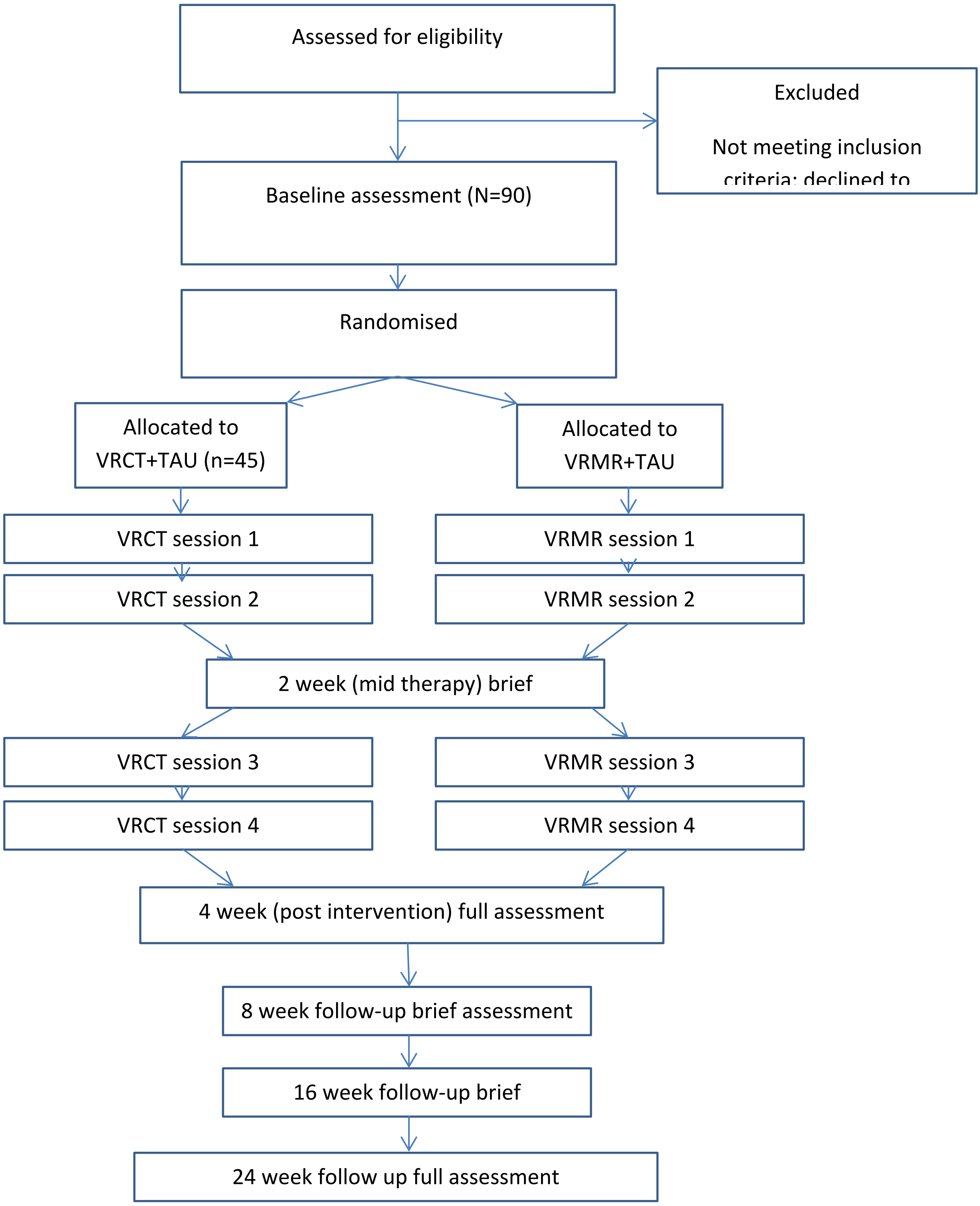

2.1 Outcomes measures

2.1.1 Primary outcome

The primary outcome measure will be conviction in the persecutory delusion (using a 0-100% scale). This is assessed at 0, 2, 4, 8, 16, and 24 weeks (primary endpoint is 4 weeks).

2.1.2 Secondary outcomes

Secondary objective 1:

1. Real world distress related to the persecutory delusion will be assessed using a behavioural avoidance task (Oxford Behavioural Avoidance Task; O-BAT). Participants are asked to create an individualized, 5-step hierarchy of real-world situations they find difficult and rate levels of distress when completing each step of the hierarchy.
    a. The maximum number of steps (from 1 – 5) at each time point will be determined based on the number of steps completed at that time point *[OBAT5YN-OBAT1YN]*. These will be recoded so that Yes=0 and No=1, implying that higher scores indicate higher avoidance.
    b. The level of distress felt when doing the task is scored on a scale from zero to 10, where higher scores indicate greater distress. The scale increases in increments of 0.5 and will be treated as a continuous measure. For the analysis, it will be necessary to compare the mean distress score of the steps completed at both baseline AND the same steps at follow-up e.g., if a participant reached step 3 at baseline and step 4 at follow-up, the mean distress score of steps 1 – 3 will be compared.

Secondary objective 2 and 4:

1. Activity will be assessed by actigraphy and a time-budget measure (Jolley et al., 2006). This is an interviewer rated measure of activity specifically designed for patients with psychosis. It measures activity over a week long period using four time points across the day. It is completed retrospectively. Each time period is rated (from 0, low to 4, high) according to how complex the activity is and the effort required over and above doing nothing. The total score ranges from 0-112. This is assessed at 0, 4, and 24 weeks (primary endpoint is 4 weeks).

    From Jolley et al. (2006):
    "The time-budget measure takes the form of a diary over a week, completed during a structured interview, with 4 time blocks for each day rated from 0–4 as below. Where more than one activity is present, the highest scoring activity is rated. There are 28 time blocks for the week, and the total possible score ranges from 0–112. Interviewers start with the preceding day and probe for activities and social contact over each time period, recording this on a diary sheet for later rating. They also check that the week is a typical or average week, and if not, complete the time budget in relation to a week chosen to be more representative. The rating system is as follows:

    0 – nothing: lying thinking, sleeping, sitting etc.

    1 – predominantly passive activity: e.g. watching TV, listening to the radio.

2 – an independent activity requiring some planning and motivation, but relatively simple or brief: e.g. a walk to the local shops to get cigarettes, tidying room, washing-up, preparing a simple meal for oneself.

3 – several 2-rated activities completely filling a time period, sounding 'busy' or a more complex and demanding but unvaried or shorter activity: e.g. a visit involving public transport, or prolonged social contact with others.

4 – time period filled with a variety of demanding independent activities requiring significant motivation and planning and with some variation in tasks: e.g. work, a course of study, a trip out requiring organisation."

2. Quality of life will be assessed by the EQ-5D-5L (http://www.euroqol.org/). This is assessed at 0, 4, and 24 weeks (primary endpoint is 4 weeks). The EQ-5D-5L index will be calculated using the cross walk method. There are five items on the EQ-5D-5L, each scored from 1 to 5 (no problem to severe problems). The cross walk method uses country specific weightings of these items to generate an overall score, which runs from negative values to 1. A score of 1 indicates no problem, whilst a negative score indicates severe problems. This index and the VAS score will be summarised and compared between groups. The VAS score is a separate question examining overall health using a scale from 0 – 100 (with 0 being "worst" health and 100 being "best").

3. Suicidal ideation will be assessed by the Columbia-Suicide Severity Rating Scale (CSSRS) (Posner et al., 2011). This is assessed at 0, 4, and 24 weeks (primary endpoint is 4 weeks). The CSSRS consists of four categories of questions. The C-SSRS is a semi-structured interview that measures suicide ideation and behaviour. The Suicidal Ideation scale, the severity scale, is a 6-point ordinal scale, with scores for those with ideation ranging from 1 (wish to be dead) to 5 (suicidal intent with plan). Those who denied ideation receive a zero. The Intensity of Ideation subscale is comprised of five items (i.e., frequency, duration, controllability, deterrents, reasons for ideation), each rated on a scale from 1 to 5 (total scores ranging from 2 to 25). We will only utilize the severity score in the main analysis, Administration time is dependent on the participant's suicidal history ranging from 1 to 2 or 5 to 10 minutes; as indicated above, certain scales may not be required for completion.

4. Overall paranoia will be assessed by the Revised GPTS (Green et al., 2008; Freeman et al., 2019). This is assessed at all timepoints (primary endpoint is 4 weeks). The Revised GPTS assessment measures two dimensions of paranoid thinking: ideas about social reference and ideas about social persecution. The social reference subscale consists of 8 statements and the persecution subscale consists of 10 statements. These statements are rated according to how true the subject believes the statement to be on a Likert scale from 0 (don't believe at all) to 4 (totally believe). The total score for each dimension is obtained by summing all responses, ranging from 0-32 for the social reference subscale and 0-40 for the persecution subscale, with higher scores reflecting higher levels of paranoia. There is also a total score which ranges from 0-72 (Freeman et al., 2019).

Secondary objective 3 and 4:

1. Delusion severity will be assessed using the delusions subscale of the PSYRATS (Haddock et al., 1999). The delusions subscale consists of 6 items: Preoccupation (Amount/Duration), Conviction, Distress (Amount/Intensity), and Disruption. Scores are summed and range between 0 and 24. This is assessed at all timepoints (primary endpoint is 4 weeks).
2. Wellbeing will be assessed by the WEMWBS (Tennant et al., 2007). This is a 14 item self-report assessment of emotional wellbeing. Each item is rated from 1 (none of the time) to 5 (all of the time). Each item contributes equally to the total score, which ranges from 14 to 70. Higher scores indicate a greater degree of psychological wellbeing. This is assessed at 0, 4, and 24 weeks (primary endpoint is 4 weeks).
3. Perceptions of recovery will be assessed by the Questionnaire about the Processes of Recovery (QPR) (Neil et al., 2009). The QPR is a 22-item self-report outcome measure of personal recovery, completed only by service users. They are asked to rate their own progress towards personal recovery. Example questions include ‘I feel that my life has a purpose’ and ‘I can recognise the positive things I have done’. Each item comprises a pro-recovery statement rated from 0 (low recovery) to 4 (high recovery). Scores are summed (range: 0-88). This is assessed at 0, 4, and 24 weeks (primary endpoint is 4 weeks.

2.1.3 Tertiary outcomes

Tertiary objective 1:

1. Use of defence behaviours will be assessed using the Safety Behaviours Questionnaire (Freeman et al., 2001). Defence behaviours are assessed by a semistructured interview. The questionnaire includes 7 different types of defence behaviours (avoidance, in-situ, escape, compliance, getting help, aggression, and other). An action is considered to be a defence behaviour if the interviewee reported that it had been carried out with the aim of reducing persecutory threat. After a defence behaviour is elicited the participant is asked to rate its frequency over the last week on a four-point scale (1=Definitely occurred on at least one occasion, 2=Occurred more than once but less than 5 times, 3=Occurred at least 5 times, 4=Occurred at least every day). If the interviewee does not report a specific type of defence behaviour it will be given a score of 0. Scores are then summed for each of the subscales (number and frequency for each type of defence behaviour), and for the questionnaire as a whole. There is an overall total score of the total number of defence behaviours used and the total frequency of all defence behaviours used.. For the analysis, we will only utilize the score for total frequency of all defence behaviours used. This is assessed at 0, 2, 4, 8, 16, and 24 weeks (primary endpoint is 4 weeks).
2. Strength of safety beliefs will be assessed using a visual analogue scale (Freeman et al., 2016). Each line is 100 mm long, and where the patient marked the line is recorded as a number between 0 and 100. This is assessed at 0, 2, 4, 8, 16, and 24 weeks (primary endpoint is 4 weeks).

2.2 Target population

Participants with persistent persecutory delusions in the context of non-affective psychosis.

2.2.1 Inclusion Criteria

- Participant is willing and able to give informed consent for participation in the trial.
- Male or Female, aged 16 years or above.

- Persistent (at least 3 months) persecutory delusion (as defined by Freeman & Garety, 2001), held with at least 50% conviction; specifically, participants will be reporting feeling threatened when with other people.
- Primary diagnosis of schizophrenia-spectrum psychosis (non-affective psychosis).

2.2.2 Exclusion Criteria

The participant may not enter the trial if ANY of the following apply:

- Primary diagnosis of alcohol or substance disorder
- Photosensitive epilepsy
- Significant visual, auditory, or balance impairment
- Current receipt of another psychological therapy
- Insufficient comprehension of English
- In forensic settings
- Organic syndrome
- Learning disability
- Current active suicidal plans

A participant may also not enter the trial if there is another factor, which, in the judgement of the investigator, would preclude the participant from providing informed consent or from safely engaging with the trial procedures. Reason for exclusion will be recorded in line with CONSORT guidelines.

In addition the following stipulation was added due to the COVID-19 pandemic:

- Upon restarting in September 2020 after the suspension of the trial due to COVID-19, recruitment suspension was continued for trial participants who have any of the conditions that would make someone high or moderate risk (clinically vulnerable) for a severe course of COVID-19 (https://www.nhs.uk/conditions/coronavirus-covid-19/people-at-higher-risk/whos-at-higher-risk-from-coronavirus/).

2.3 Sample size

Based on the pilot test against exposure (Freeman et al, 2016), we expect at least a 20% reduction in delusional conviction (effect size=1.0) for VRCB compared to VRMR. This is a conservatively lower effect size expectation than the first pilot, despite the first pilot using a briefer VRCB and a control condition that included one part of active treatment. Nonetheless, we recognise that an even lower effect size (d=0.75, reflecting a 15% reduction in conviction) for delusions would still be of interest to pursue and thus we power the full trial (n=90) on this basis. Following guidelines for good practice (Lancaster et al, 2004), interim analysis will provide simple descriptive statistics and an initial estimate of the 95% confidence interval for the treatment effect. This interim analysis of the week 4 data after 30 participants, will provide an estimate of conditional power (i.e. power given the data obtained so far - Whitehead & Matsushita, 2003; Snapinn et al., 2006).

We will stop the trial if the interim estimate of effect size, d, is 0.1 or lower, implying that the conditional power of the full trial, based on the interim results and the hypothesised effect size of 0.75, would be 60%

or lower. If it were assumed that the treatment effect seen in the pilot would continue throughout the rest of the trial, then the conditional power would be as low as 3%.

2.4 Randomisation and blinding in the analysis stage

Randomisation will occur after completion of the baseline assessment. Allocation to VRCB and VRMR will be 1:1. Randomisation will be carried out by an online system designed by the University of Oxford Primary Care Clinical Trials Unit. Randomisation using a permuted blocks algorithm, with randomly varying block size, will be stratified by severity of delusion (moderate (50-75% conviction)/high (76%+ conviction)).

The trial assessors will be blind to group allocation, but the patients and trial therapists will not be (they cannot be blinded to what psychological treatment is delivered or received). The trial therapists will inform patients of the randomisation outcome so that the research assessors remain blind to group allocation. Precautionary strategies to prevent breaks of blind include: the therapist and assessor considering room use and booking arrangements; patients being reminded by the assessor not to talk about treatment allocation; and, after the initial assessment, the assessor not looking at the patient's clinical notes. If an allocation is revealed between assessment sessions, this is logged by the trial coordinator/PI and re-blinding will occur using another assessor.

3 Analysis – General considerations

3.1 Descriptive statistics

Summary descriptions for continuous measurements will be means and standard deviations. Medians and interquartile ranges will be also presented if more appropriate. Counts and percentages will be presented for categorical variables. Summary statistics will be provided by randomised group and overall.

3.2 Characteristics of participants

Baseline characteristics of the patients (demographics and baseline of all outcome variables where available) will be reported by randomised group as well as overall.

There will be no tests of statistical significance nor confidence intervals for differences between randomised groups on any baseline variables.

3.3 Definition of population for analysis

The primary statistical analysis of efficacy outcomes will be carried out on the full analysis dataset. That is, after randomisation, participants will be analysed according to their allocated intervention group irrespective of what intervention they actually receive. Every effort will be made for full follow-up data on every participant to allow for as complete as possible analysis. If follow up data is missing for some participants, then any available data for those participants will be included in the primary analysis, under the missing at random (MAR) assumption.

3.4 Pooling of investigational sites

Participants are recruited from multiple NHS Trusts, but assessment and therapy will be administered by researchers/clinicians from one site. Clustering by NHS Trust will not be accounted for in the analysis.

3.5 Data Monitoring Committee And Interim Analyses

Following guidelines for good practice (Lancaster et al, 2004), interim analysis will provide simple descriptive statistics and an initial estimate of the 95% confidence interval for the treatment effect. This interim analysis of the week 4 data after 30 participants, will provide an estimate of conditional power (i.e. power given the data obtained so far - Whitehead & Matsushita, 2003; Snapinn et al., 2006).

We will stop the trial if the interim estimate of effect size, d, is 0.1 or lower, implying that the conditional power of the full trial, based on the interim results and the hypothesised effect size of 0.75, would be 60% or lower. If it were assumed that the treatment effect seen in the pilot would continue throughout the rest of the trial, then the conditional power would be as low as 3%.

4 PRIMARY ANALYSIS

4.1 Primary outcome

The primary hypothesis is for change in the primary outcome measure, conviction in the persecutory delusion (using a 0-100% scale) at 4 weeks. Additionally, repeated measures are also assessed at the 2 week point and again at 8, 16, and 24 weeks. A mixed effects model will be fitted to the repeated measures to estimate treatment effects. The mixed effect models will include the outcome as the response variable, time point, randomised group, severity of delusion (rated as moderate (50-75% conviction)/high (76%+ conviction)),, and baseline score as fixed effects and a patient specific random intercept. An interaction between time and randomised group will be fitted as a fixed effect to allow estimation of treatment effect at all time points. A random effect will account for repeated measurements from the same participant. The main treatment effect will be presented as the adjusted mean difference between groups at 4 weeks, alongside a p-value and 95% confidence interval. We will also calculate an effect size (Cohen's d), which is the between group treatment difference divided by the shared standard deviation at baseline.

4.2 Handling missing data

Missing data on individual measures will be pro-rated if more than 90% of the items are completed; otherwise the measure will be considered as missing.

The availability of the outcome data for the primary outcome will be summarised by randomised group.

The mixed effects model implicitly accounts for data missing at random, however the data missingness mechanism will be explored. Logistic regression models will explore any association between baseline characteristics and availability of the primary outcome. Covariates found to be predictive of missingness will be included in the primary analysis as a sensitivity test.

4.3 Handling outliers

Any outliers will be checked and verified to ensure that they are true values. Outliers will be identified as those observations more than four standard deviations from the mean. Once they have been confirmed, a sensitivity analysis will be carried out to assess the impact of these values on the results by excluding these participants.

4.4 Handling multi-centre/clustered data

This is a single centre trial. Although we are recruiting from multiple NHS Trusts, assessment and therapy will be provided by staff from one site. Therefore there will be no clustering of the outcome by site.

4.5 Multiple comparisons and multiplicity

The primary outcome is clearly stated in the protocol and no adjustments for multiple comparisons will be made.

4.6 Model assumptions

The distribution of the primary outcome will be assessed and the assumptions of the models will be checked. If any of the assumptions are violated, then p-values and confidence intervals for the model coefficients will be obtained by means of bootstrapping.

5 SECONDARY ANALYSIS

5.1 Secondary outcomes

Summaries at each time point will be presented for all secondary outcomes both overall and by treatment arm. Summary statistics will be presented as means and standard deviations for all continuous outcome measures (or median and interquartile range if non-normally distributed). For categorical outcomes (i.e. avoidance scores) number and percentages will be presented.

The outcomes listed below will be analysed in the same way as the primary outcome. A linear mixed effect model will include the outcome as the response variable, time point, randomised group, severity of delusion, and baseline score as fixed effects and a patient specific random intercept. An interaction between time and randomised group will be fitted as a fixed effect to allow estimation of treatment effect at all time points. A random effect will account for repeated measurements from the same participant. For the EQ-5D-5L measure, then p-values and confidence intervals for the model coefficients will be obtained by means of bootstrapping.

1.) Real-world avoidance and distress O-BAT
2.) Actigraphy and time budget measure
3.) EQ-5D-5L cross-walk index and VAS scores
4.) Suicide ideation CSSRS
5.) Paranoia R-GPTS
6.) Delusion severity PSYRATS
7.) Wellbeing WEMWBS
8.) Perceptions of recovery QPR

TABLE 1 SAMPLE TABLE FOR PRESENTATION OF SECONDARY OUTCOMES

	Intervention	Control	Overall
**Baseline, mean (sd)**			
**2 weeks, mean (sd)**			
**4 weeks, mean (sd)**			
**8 weeks, mean (sd)**			
**16 weeks, mean (sd)**			
**24 weeks, mean (sd)**			
**Estimated treatment difference at 4 weeks*, [95% C.I.], p-value**			
**Follow-up:**			
**Estimated treatment difference at 8 weeks, [95% C.I.], p-value**			
**Estimated treatment difference at 16 weeks, [95% C.I.], p-value**			
**Estimated treatment difference at 24 weeks, [95% C.I.], p-value**			

*Primary endpoint

5.1.1 Real world avoidance and distress (O-BAT)

Real world avoidance and distress as assessed by the O-BAT will be analysed where available, i.e. in those participants randomised before September 2020. The task will be summarised both overall and by group in terms of numbers and percentages completing each step of the task (avoidance score) and mean, standard deviation, median, interquartile range and range of the level of distress for each step for those that completed that step (distress score). Additionally the highest step completed will be summarised by number and percentage of participants by group and distress level.

At 4 weeks we will test both whether the number of steps completed has changed (Avoidance) and whether distress has reduced (Distress).

SAMPLE TABLE FOR REAL WORLD DISTRESS OUTCOME – STEPS COMPLETED

	Intervention			Control			Overall		
	**Baseline**	**6 weeks**	**26 weeks**	**Baseline**	**6 weeks**	**26 weeks**	**Baseline**	**6 weeks**	**26 weeks**
**Completed step 1, n (%)**									
**Distress level**									
Mean (SD)									
Median (IQR)									
Range									
**Completed step 2, n (%)**									
**Distress level**									
Mean (SD)									
Median (IQR)									
Range									
**Completed step 3, n (%)**									
**Distress level**									
Mean (SD)									
Median (IQR)									
Range									
**Completed step 4, n (%)**									
**Distress level**									
Mean (SD)									
Median (IQR)									
Range									
**Completed step 5, n (%)**									
**Distress level**									
Mean (SD)									
Median (IQR)									
Range									
**Total steps completed, n (%)**									

**Distress level**									
Mean (SD)									
Median (IQR)									
Range									

SAMPLE TABLE FOR REAL WORLD DISTRESS OUTCOME – HIGHEST STEP COMPLETED

Highest step completed	Intervention			Control			Overall		
	Baseline	6 weeks	26 weeks	Baseline	6 weeks	26 weeks	Baseline	6 weeks	26 weeks
**1, n (%)**<br>**Distress level**<br>Mean (SD)<br>Median (IQR)<br>Range									
**2, n (%)**<br>**Distress level**<br>Mean (SD)<br>Median (IQR)<br>Range									
**3, n (%)**<br>**Distress level**<br>Mean (SD)<br>Median (IQR)<br>Range									
**4, n (%)**<br>**Distress level**<br>Mean (SD)<br>Median (IQR)<br>Range									
**5, n (%)**<br>**Distress level**<br>Mean (SD)<br>Median (IQR)<br>Range									

**Overall distress, n (%)**									
**Distress level**									
Mean (SD)									
Median (IQR)									
Range									

5.1.2 Actigraphy and time budget measure

Actigraphy measures the number of steps taken using activity watches over approximately one week. It is measured at the baseline, 4 week, and 24 week time points. An average number of steps per day will be calculated using the number of steps taken over the 7-day period. There will be a minimum requirement of 3 days' worth of step data to calculate the average. Participants who wear the activity watch for less than 3 days will be considered missing data. Summaries of number of steps taken will be presented as means and standard deviations (or median and interquartile range if non-normally distributed) at each time point both within each group and overall. Number of steps taken will be analysed using a linear mixed effect model with a random effect for participant. Fixed effects in the model will include: time point, randomised group, baseline number of steps and severity of delusion at baseline. An interaction between time and randomised group will be fitted as a fixed effect to allow estimation of treatment effect at all time points. This outcome is measured at baseline, 4 weeks and 24 weeks.

Time budget measure will be analysed with a linear mixed effects model in the same way as the primary outcome. Time budget is measured over 7-day period using four time points across the day and is completed retrospectively. At each time point, the activity is rated from 0 (low) to 4 (high) according to how complex the activity is and the effort required over and above doing nothing. A total score will be calculated from the sum of the individual scores over all days and times within each day to give a value ranging from 0-112.The main treatment effect for both number of steps taken and time budget will be presented as the adjusted mean difference between groups at 4 weeks, alongside a p-value and 95% confidence interval.

5.1.3 Quality of life; suicide ideation and, overall paranoia

EQ-5D-5L cross-walk index and VAS scores, Suicidal ideation severity (CSSRS) and Paranoia (R-GPTS) will be analysed with a linear mixed effects model in the same way as the primary outcome. The EQ-5D-5L cross-walk will be calculated using country-specific weighting of each item for the United Kingdom (see https://www.unmc.edu/centric/_documents/EQ-5D-5L.pdf), whilst the VAS scores are continuous measures on a scale of 0 to 100. GPTS scores will be calculated from the total of all items within the scale. All items on the CSSRS will be summarised but only the severity scale will be analysed. The main treatment effect will be presented as the adjusted mean difference between groups at 4 weeks, alongside a p-value and 95% confidence interval.

5.1.4 Delusional severity, wellbeing, and perceptions of recovery

Delusion severity (PSYRATS), Wellbeing (WEMWBS), and Perceptions of recovery (QPR) will be analysed with a linear mixed effects model in the same way as the primary outcome. Each of these measures will be calculated as a total score from all items within each questionnaire (PSYRATS range 0 to 24, WEMWBS range 14 to 70 and QPR range 0 to 88). The main treatment effect will be presented as the adjusted mean difference between groups at 4 weeks, alongside a p-value and 95% confidence interval.

5.1.5 Maintenance of benefits over time

Maintenance of treatment benefits over time will be estimated from the linear mixed effects models for each outcome. Treatment effects at follow-up time point 24 weeks (and 8 and 16 weeks where these measures are available) will be estimated alongside p-values and associated 95% confidence intervals. Contrasts between the treatment effect at 4 week primary end point and follow-up time points will be

estimated from the model in order to determine whether any treatment effect has been maintained at follow-up.

6 TERTIARY ANALYSIS

6.1 Mediation analysis

The mediation analysis will investigate putative mediational factors (safety behaviours and safety beliefs) using modern causal inference methods (Dunn et al, 2015). This involves using parametric regression models to test for mediation of VRCB on outcome through the putative mediators. Analyses will adjust for baseline measures of the mediator, outcomes, and possible measured confounders. We will include repeated measurement of mediators and outcomes to account for classical measurement error and baseline confounding.

Measurement error models, as described by Dunn et al, 2015, involve estimating a latent variable for the mediator at each time point representing the "true" value of the mediator. The model is identified by assuming the variance of the measurement error remains constant over time. Figure 1 shows an example path diagram for the measurement error mediation model. The correlation between baseline mediator and treatment allocation is constrained to be zero, under the assumption that there will be no baseline imbalance under randomisation.

The mediation effect will be estimated at two different time points. Firstly, the mid-treatment value of the mediator is used in the analysis model, along with the end of treatment value of the outcome. This is based on a model of causality, i.e. that change in the mediator will occur before change in outcome. The model is adjusted for baseline values of mediator and outcome. This model will be estimated using full information maximum likelihood, a method which produces estimates that are valid under the missing at random assumption. Although the main time points of interest are mid-treatment (2 weeks) and end of treatment (4 weeks), measures of the mediator at other time points will be included in the model to aid with estimation in the presence of missing data.

Secondly, since change in the outcome is expected to occur relatively soon after change in the mediator, contemporaneous measures of the mediator-outcome relationship will also be investigated. That is measures of the mediator and outcome at 4 weeks. As temporal precedence cannot be easily established when mediator and outcome are measured at the same time, this model will also be investigated for possible reverse causality. In addition to modelling safety behaviours and safety beliefs at 4 weeks as mediators of the effect of treatment on conviction in the persecutory delusions at 4 weeks, a model will be fitted to estimate whether conviction in the persecutory delusions at 4 weeks mediates change in safety behaviours and safety beliefs at 4 weeks. The indirect effects from the two causal models will be compared.

The mediation models assume no unobserved confounding of the mediator-outcome relationship and by adjusting for baseline values of mediator and outcome a major potential source of confounding has been taken into account. The full information maximum likelihood method assumes multivariate normality. Confidence intervals for the indirect effects will be estimated using bootstrapping. Model fit will be assessed using the root means squared error of approximation (RMSEA) and a likelihood ratio test comparing against a null model. Models will be fitted using the structural equation model builder in STATA 16.

In the case that the model cannot be identified, it will be conducted using the approach of Baron and Kenny (1986), but will follow the adaptation in Freeman et al. (2017) which makes use of linear mixed effects models. This is valid under a MAR assumption but does not account for measurement error in the mediator.

FIGURE 1 EXAMPLE PATH DIAGRAM FOR THE MEASUREMENT ERROR BASED MEDIATION MODEL

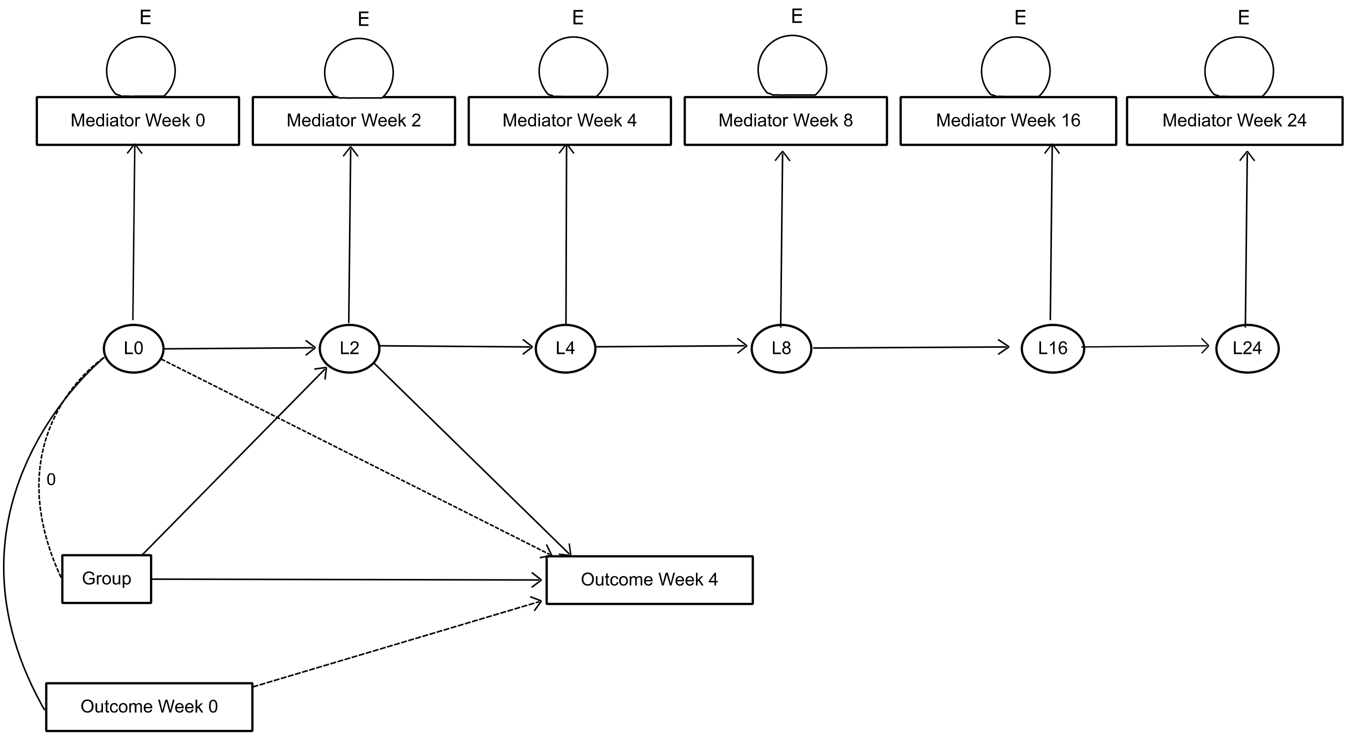

7 SENSITIVITY ANALYSIS

7.1 Outliers and missingness assumptions

If outliers are identified (defined as more than 4 standard deviations from the mean), a sensitivity analysis excluding these outliers will be carried out to determine the impact of these observations on the treatment effect of the primary outcome.

As a sensitivity analysis of the primary outcome, baseline covariates found to be predictive of missingness will be included as main effects in the linear mixed effects model.

7.2 Impact of the COVID-19 Pandemic

It is hypothesised that the effect of the COVID-19 pandemic will be detrimental in both arms but that participants in the intervention arm will still have better outcomes. However this might lead to some treatment effect dilution. Conversely the intervention might offer some protective effect and lead to a larger treatment effect during the lockdown period. Sensitivity analyses will explore whether there is a difference in the treatment effect at 4 weeks and at 24 weeks when measures taken after the beginning of lockdown. Two analyses will be conducted. The first will include all participants who completed their 4 week measures before the beginning of lockdown on the 16th March 2020 but setting any follow-up measures that were completed after lockdown as missing. This model will be used to explore whether

there is a difference in the treatment effect at the primary endpoint (4 weeks). In order to explore whether there is a difference in the treatment effect at follow-up timepoints, a further sensitivity analysis of the primary outcome will be carried out including only those who completed their 24 weeks measures before the beginning of lockdown.

7.3 Medication effects

A sensitivity analysis will explore whether treatment effects might be attributable to increased prescribing of medication. Prescription of antipsychotic medication will be gathered at baseline, 4 weeks and 24 weeks. This will be converted into a chlorpromazine equivalent (CPZ score). In the first instance a linear mixed model, similar to the primary analysis, will be fitted to test whether there is a difference in prescribing of medication by treatment arm. Only if this analysis is statistically significant, a mediation model similar to the one described in section 6.1, will be fitted to test whether any of the treatment effect is mediated by changes in prescribing of medication.

7.4 Credibility Effects

A sensitivity analysis will explore whether treatment effects differ by credibility and expectancy ratings. We will use the Credibility and Expectancy Questionnaire (Devilly & Borkovec, 2000). This questionnaire examines treatment expectancy and rationale credibility and has two subscales: cognitively based credibility and affectively based expectancy. If there is an indication of a discrepancy between the groups on either of these subscales, we will do a sensitivity analysis on the main outcome controlling for the relevant subscale(s).

8 SUBGROUP ANALYSES

No subgroup analyses were specified in the protocol.

9 SAFETY ANALYSIS

9.1 Adverse events

This is considered to be a low risk trial. Any serious adverse events will be recorded. Serious adverse events are defined as: deaths; suicide attempts; serious violent incidents; and admissions to secure units. Summary counts and percentages of adverse events will be reported at the end of the trial.

10 VALIDATION

A second Trial Statistician will validate the primary outcome and safety data analyses by independent programming.

11 CHANGES TO THE PROTOCOL OR PREVIOUS VERSIONS OF SAP

The CHOICE was removed on 12th April, 2019 from the participant's assessment list in order to reduce the assessment length.

12 References

Castle, DJ., Phelan, M., Wessely, S., and Murray, RM. (1994). Which patients with non-affective functional psychosis are not admitted at first psychiatric contact? British Journal of Psychiatry, 165, 101–06.

Devilly, G. J. & Borkovec, T. D. (2000). Psychometric properties of the credibility/expectancy questionnaire. J Behav Ther Exp Psychiatr, 31(2), 73-86.

Dunn G., Emsley R., Liu H., et al. (2015). Evaluation and validation of social and psychological markers in randomised trials of complex interventions in mental health: a methodological research programme. Southampton (UK): NIHR Journals Library; 2015 Nov. (Health Technology Assessment, No. 19.93.) Chapter 2, Treatment effect mediation. Available from: https://www.ncbi.nlm.nih.gov/books/NBK326949/

Freeman, D., Garety, P.A., and Kuipers, E. (2001). Persecutory delusions: developing the understanding of belief maintenance and emotional distress. Psychol Med., 31(7), 1293-306.

Freeman, Dunn et al (2015) Effects of cognitive behaviour therapy for worry on persecutory delusions in patients with psychosis (WIT). Lancet Psychiatry, 2, 305-313.

Freeman, D., Bradley, J., Antley, A., Bourke, E., DeWeever, N., Evans, N., Černis, E., Sheaves, B., Waite, F., Dunn, G., Slater, M., & Clark, D. (2016). Virtual reality in the treatment of persecutory delusions. British Journal of Psychiatry, 209, 62-67.

Freeman, D. (2016). Persecutory delusions: a cognitive perspective on understanding and treatment. Lancet Psychiatry, 3, 685-692.

Freeman, D., Loe, B. S., Kingdon, D., Startup, H., Molodynski, A., Rosebrock, L., Brown, P., Sheaves, B., Waite, F., & Bird, J. C. (2019). The revised Green *et al.* Paranoid Thoughts Scale (R-GPTS): psychometric properties, severity ranges, and clinical cut-offs. *Psychological Medicine*, 1-10.

Freeman, D., Sheaves, B., Goodwin, G. M., Yu, L. M., Nickless, A., Harrison, P. J., ... & Hinds, C. (2017). The effects of improving sleep on mental health (OASIS): a randomised controlled trial with mediation analysis. *The Lancet Psychiatry*, *4*(10), 749-758.

Garcia-Palacios et al (2007). Comparing acceptance and refusal rates of virtual reality exposure vs. in vivo exposure by patients with specific phobias. Cyberpsychology Behavior, 10, 722-724.

Green et al (2008). Measuring ideas of persecution and reference. Psychological Medicine, 38, 101-111.

Haddock, G., McCarron, J., Tarrier, N. & Faragher, F. B. (1999). Scales to measure dimensions of hallucinations and delusions: the psychotic symptom rating scales (PSYRATS). Psychological Medicine, 29, 879-889.

Hjorthøj, C., Stürup, A E., McGrath, J J., and Nordentoft, M. (2017). Years of potential life lost and life expectancy in schizophrenia: a systematic review and meta-analysis. Lancet Psychiatry, 4, 295-301.

Hor, K. and Taylor, M. (2010). Suicide and schizophrenia: a systematic review of rates and risk factors. Journal of Psychopharmacology, 24 (11), suppl. 4, 81-90.

Jolley, S., Garety, P.A., Ellett, L., Kuipers, E., Freeman, D., Bebbington, P.E., Fowler, D.G., & Dunn, G. (2006). A validation of a new measure of activity in psychosis. Schizophrenia Research, 85, 288-295.

Kennedy, J. L., Altar, C. A., Taylor, D. L., Degtiar, I., and Hornberger, J. C. (2014). The social and economic burden of treatment-resistant schizophrenia: a systematic literature review. International Clinical Psychopharmacology, 29 (2), 63-76.

Morina, N., Iintema, H., Meyerbröker, K., & Emmelkamp, P. (2015). Can virtual reality exposure therapy gains be generalized to real-life? A meta-analysis of studies applying behavioral assessments. Behaviour Research Therapy, 74, 18-24.

Neil, S., Kilbride, M., Pitt, L., Nothard, S., Welford, M., Sellwood, W., Morrison, A. (2009). The questionnaire about the process of recovery (QPR): A measurement tool developed in collaboration with service users. Psychosis, Psychological, Social and Integrative Approaches, 1, 145-155.

Opris et al (2012). Virtual reality exposure therapy in anxiety disorders. Depression Anxiety, 29, 85-93.

Posner, K., Brown, G., Stanley, B., Brent, D., Yershova, K., Oquendo, M., Currier, G., Melvin, G., Greenhill, L., Shen, S., & Mann, J. (2011). The Columbia-Suicide Severity Rating Scale. American Journal of Psychiatry, 168, 1266-1277.

Snapinn, S., Chen, M.-G., Jiang, Q. & Koutsoukos, T. (2006). Assessment of futility in clinical trials. Pharmaceutical Statistics, 5, 273-281.

Tennant, R., Hiller, L., Fishwick, R., Platt, S., Joseph, S., Weich, S. et al. (2007). The Warwick-Edinburgh Mental Well-being Scale (WEMWBS): development and UK validation. Health and Quality of Life Outcomes, 5, 63-80.

Tully, S., Wells, A., and Morrison, A. P. (2016). An exploration of the relationship between use of safety-seeking behaviours and psychosis: a systematic review and meta-analysis. Clinical Psychology and Psychotherapy, 1-22.

van der Gaag, M., Valmaggia, L, R., and Smit, F. (2014). The effects of individually tailored formulation-based cognitive behavioural therapy in auditory hallucinations and delusions: A meta-analysis. Schizophrenia Research, 156, 30-37.

Whitehead, J. & Matsushita, T. (2003). Stopping clinical trials because of treatment ineffectiveness: a comparison of a futility design with a method of stochastic curtailment. Statistics in Medicine, 22, 677-687.

13 Appendices

13.1 Appendix I. Schedule of procedures

Procedures	Screening	Baseline (0 weeks)	VR session 1	VR session 2	Mid intervention (2 weeks)	VR session 3	VR session 4	Post intervention (4 weeks)	Follow-up (8 weeks)	Follow-up (16 weeks)	Follow-up (24 weeks)
Eligibility assessment	X	X (brief check)									
Informed consent		X									
Demographics		X									
Randomisation		X									
Delusion conviction (0-100%)		X			X			X	X	X	X
PSYRATS – delusion		X			X			X	X	X	X
R-GPTS		X			X			X	X	X	X
Real world avoidance and distress		X						X			
Activity (actigraphy, time-budget)		X						X			X
EQ-5D-5L		X						X			X
Columbia suicide severity scale		X						X			X
Mediators (SBQ, safety beliefs)		X			X			X	X	X	X
WEMWBS		X						X			X
QPR		X						X			X
Service receipt (CSRI)		X									X
VR simulator sickness questionnaire			XX								
Credibility/expectancy measure			X								
VRCB or VRMR intervention			X	X		X	X				

13.2 Appendix II. Flow diagram of trial participants

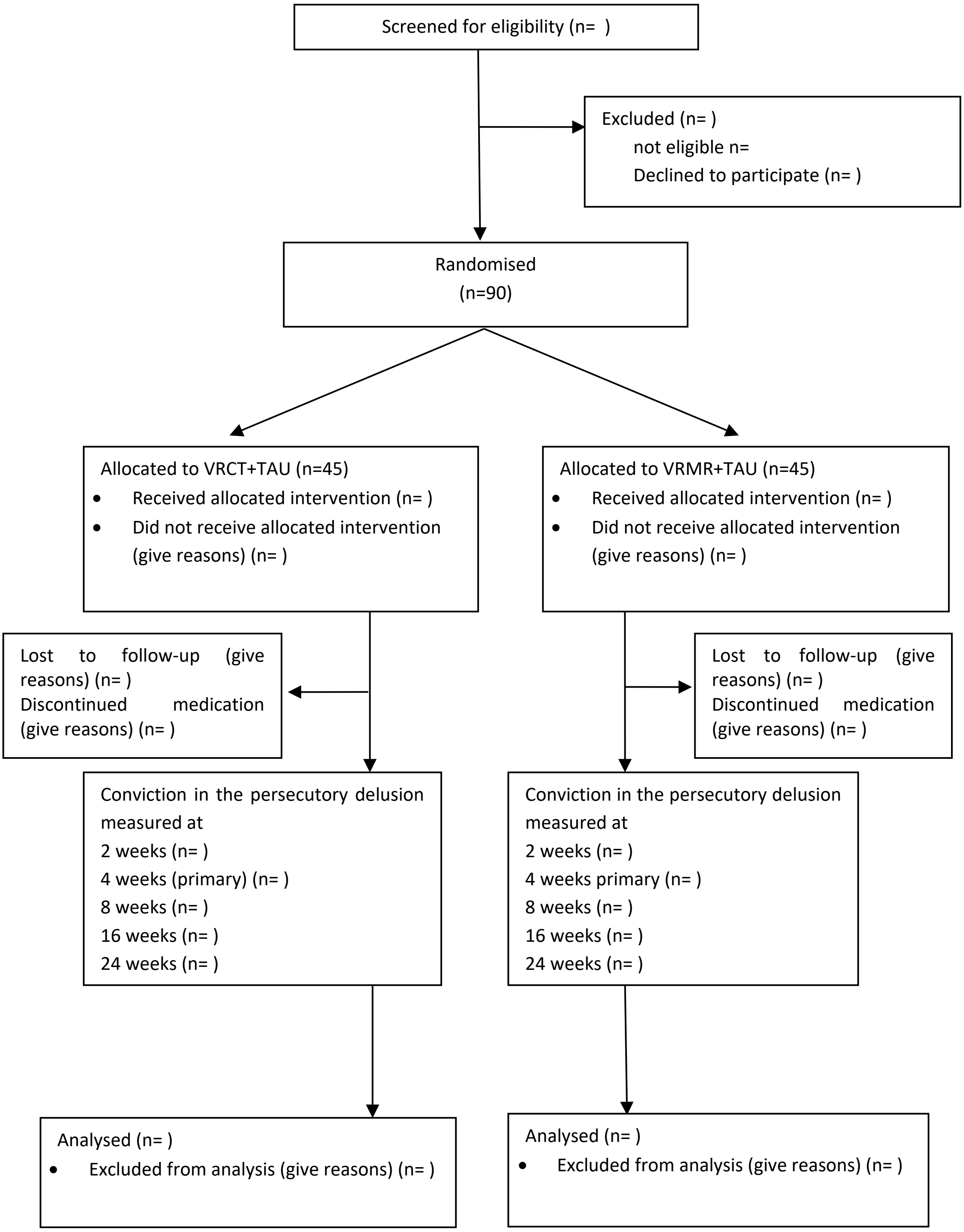

Primary Care Clinical Trials Unit

ST104-A:

STATISTICAL ANALYSIS REPORT

the thrive trial

The THRIVE study: A randomized controlled trial comparing Virtual Reality Confidence Building with VR Mental Relaxation for people with fears about others.

CONFIDENTIAL

Version 3.0

12 July 2023

Based on Protocol version 4.3, 28th April 2021

Based on SAP Version 4.0, 20th December 2021

Author: Ushma Galal

Version History

Version:	Version Date:	Changes:
0.1	7 June 2022	original
0.2	22 June 2022	Updates post internal review
0.3	4 July 2022	Updates post internal review
0.4	7 July 2022	Response to comment regarding EQ-5D bootstrapping

Version:	Version Date:	Changes:
0.5	27 July 2022	Include post-hoc analysis on credibility and expectancy scores (section 4)
0.6	10 January 2023	Correcting categories for "Total number of mental health admissions in the last 24 weeks (BL), n (%)" in the baseline table; add results for further mediation analysis to section 4.2
0.7	31 January 2023	Remove "(not shown but checked)" on page 31 as results added to the table; remove irrelevant text in table on page 42
0.8	20 February 2023	Add regression of persecutory delusion on mediators to section 4.2
0.9	8 March 2023	Correction to interpretation in section 4.2
1.0	5 April 2023	Finalised version (masked)
1.1	5 April 2023	Updated with unblinded allocations & sensitivity analysis on Impact of COVID-19 corrected (Table 7)
2.0	5 April 2023	Finalised version with unmasked allocations & SAE table redacted to remove identifiable information (Table 9)
2.1	11 July 2023	Addition of reviewer requested subgroup analysis (Appendix III)
3.0	12 July 2023	Finalised version with additional subgroup analysis

Table of contents

Table of contents 71

List of tables 72

List of figures 72

1 Introduction 74

1.1 Validation 74

1.2 Software employed 75

2 Methods 75

2.1 Background Information 75

2.2 Trial/Study design 75

2.3 Objectives 77

2.4 Target population 77

2.4.1 Inclusion Criteria 77

2.4.2 Exclusion Criteria 77

2.5 Interventions 78

2.6 Outcomes measures 78

2.7 Sample size 80

2.8 Randomisation and blinding in the analysis stage 80

2.9 Data cleaning 81

2.10 Analysis for Data Monitoring and ethics Committee meetings 81

2.11 Definition of population for analysis 81

2.12 Deviation from SAP 81

3 Results 82

3.1 Representativeness of Study Sample and Patient Throughput 82

3.2 Recruitment 83

3.3 Baseline characteristics of participants 84

3.4 Number analysed 87

3.5 Primary Analyses 89

3.5.1 Predictors of missing primary outcome data 89

3.5.2 Primary & Secondary Outcomes Analyses 93

3.6 Tertiary (Mediation) Analyses 95

3.7 Sensitivity analyses 98

3.8 Subgroup analyses 98

3.9 Safety analyses 100

4 Additional exploratory analysis not specified in the SAP 102

4.1 Post-hoc investigation of Credibility and Expectancy rating 102

4.2 Post-hoc analysis on mediators 106

5 References 107

6 Appendices108

6.1 Appendix I. Diagnostic plots 108

6.2 Appendix II. O-BAT Summary Tables 122

List of tables

Table 1: Baseline characteristics by randomised group 84

Table 2 Completion of follow-up assessments, withdrawals, and lost to follow-up over the study period 87

Table 3: Association between randomised group and availability of primary outcome at 4 weeks 89

Table 4: Baseline characteristics of participants by completeness of Persecutory belief conviction at 4 weeks 89

Table 5: Summary statistics for the primary and secondary outcomes and the treatment difference between the randomised groups 93

Table 6: Mediation analysis on persecutory belief conviction using mixed-effects models 97

Table 7: Summary statistics and the treatment difference between the randomised groups for the sensitivity analyses 98

Table 8: Summary of Safety Events by randomised group 100

Table 9: Summary of SAEs per participant 101

Table 10: SPEARMAN CORRELATION TEST FOR CREDIBILITY AND EXPECTANCY vs. Persecutory delusion (all time points) 105

Table 11: Summary of steps completed for the real world distress outcome (O-BAT), by randomised group .122

Table 12: Summary of highest step completed for the real world distress outcome (O-BAT), by randomised group 123

List of figures

Figure 1: Participant flow diagram 83

Figure 2: Histograms FOR CONVICTION IN THE PERSECUTORY DELUSION (all time points) and credibility & expectancy scores 102

Figure 3: Scatterplots of Persecutory delusion (all time points) vs. Credibility score 103

Figure 4: SCATTERPLOTS OF PERSECUTORY DELUSION (ALL TIME POINTS) VS. Expectancy SCORE 103

Figure 5: Histograms and model residual plots for conviction in the persecutory delusion (primary outcome)108

Figure 6: Histograms and model residual plots for O-BAT - maximum number of steps avoided 109

Figure 7: Histograms and model residual plots for O-BAT - Mean distress score 110

Figure 8: Histograms and model residual plots for (Actigraphy) Mean number of steps (daily) 111

Figure 9: Histograms and model residual plots for the Time budget score 112

Figure 10: Histograms and model residual plots for the EQ-5D-5L INDEX 113

Figure 11: Histograms and model residual plots for the EQ5D VAS score 114

Figure 12: Histograms and model residual plots for the Columbia Suicide Severity Rating Scale (C-SSRS) total score 115

Figure 13: Histograms and model residual plots for the R-GPTS-A (social reference) score.............................116

Figure 14: Histograms and model residual plots for the R-GPTS-B (persecution) score 117

Figure 15: Histograms and model residual plots for the R-GPTS (overall) score...118

Figure 16: Histograms and model residual plots for the Delusion severity (PSYRATS) score............................119

Figure 17: Histograms and model residual plots for the Wellbeing (WEMWBS) score.....................................120

Figure 18: Histograms and model residual plots for the Process of recovery (QPR) total score.......................121

1 Introduction

This document details the analysis for the main paper(s) reporting results from the Medical Research Council Developmental Pathway Funding Scheme (MRC DPFS) funded THRIVE Randomised Controlled Trial a virtual reality (VR) therapy in the treatment/management of persecutory delusions. The results reported in these papers follow the strategy set out in the statistical analysis plan. Subsequent analyses of a more exploratory nature will not be bound by this strategy, though they are expected to follow the broad principles laid down here.

The analysis strategy will be available on request when the principal papers are submitted for publication in a journal. Suggestions for subsequent analyses by journal editors or referees, will be considered carefully, and carried out as far as possible in line with the principles of this analysis strategy; if reported, the source of the suggestion will be acknowledged.

This report is based on the statistical analysis plan *ST101-A_Statistical_Analysis_Plan_SAP_THRIVE_v4.0 20Dec2021.docx*. Any deviations from the statistical analysis plan will be described and justified in this report of the trial.

Trial/Study statistician(s):

Ushma Galal*:* ushma.galal@phc.o.ac.uk

Validation statistician(s):

Nicola Williams: Nicola.williams@phc.ox.ac.uk

Chief Investigator:

Professor Daniel Freeman*:* daniel.freeman@psych.ox.ac.uk

Trial/Study Manager:

Laina Rosebrook: laina.rosebrock@psych.ox.ac.uk

Sinéad Lambe: sinead.lambe@psych.ox.ac.uk

Data Manager:

Sinéad Lambe: sinead.lambe@psych.ox.ac.uk

1.1 Validation

Validation of results presented in this report was conducted by Nicola Williams. The primary and safety endpoints were validated by independent programming using STATA 16. Results from Stata output were checked for transcription errors. Further details of validation including validation programs are saved on the PC-CTU restricted drive in the project folder in the subfolder “STATS\4. Analysis\6.Validation -Name of Validater”.

1.2 Software employed

Stata (SE) version 16.1 SE was used for all analyses.

2 Methods

2.1 Background Information

Persecutory delusions are unfounded beliefs that others are trying to harm the person (e.g. ‘People know what I'm thinking and will kill me’). Approximately 220,000 people in England and Wales have a diagnosis of schizophrenia and about 70% of patients with schizophrenia have this psychotic experience. Persecutory delusions have a substantial impact for patients; they typically lead to social withdrawal and predict hospital admission (Castle et al., 1994) and even suicide (Hor & Taylor, 2010). The total annual cost to the public sector in England is over £7 billion. Life expectancy is, on average 14.5 years shorter for people with these problems (Hjorthøj et al, 2017).

Virtual reality has been successfully shown to treat anxiety disorders. Meta-analyses for VR exposure in the treatment of anxiety disorders, show that the effects are large (d=1.1), maintain over years, generalise to real life, and are as good as using real life exposure (Opris et al, 2012; Morina et al, 2015). It is also the most popular treatment choice when offered, preferred over real life exposure (e.g. Garcia-Palacios et al, 2007). Using VR, patients with anxiety disorders can go into feared situations and drop defences; this helps patients learn, by direct experience, that they are safe and that fears are unrealistic.

VR may be used in a similar way, to help patients with psychosis re-learn safety. In a pilot study (Freeman et al., 2016), conducted by the research team, a single session of our theoretically driven VR cognitive treatment (going into VR feared situations with dropping of defence behaviours) led to a large reduction in the delusions (d=1.3) directly compared to an alternative active treatment (VR exposure treatment). Benefits transferred to the real world; VR cognitive treatment led to a 19.6% greater reduction in distress following a real world behavioural task compared to VR exposure. The pilot study included 30 patients with persecutory delusions, using a lab-based VR treatment. The proposed study will test a more comprehensive, affordable and portable, ‘plug and play’ version of the VR cognitive treatment: VR Confidence Building (VRCB). This will be tested in a larger sample of patients.

2.2 Trial/Study design

The design is a parallel group randomised controlled trial with single blind assessment to test whether the new psychological treatment (VRCB) will reduce persecutory delusions more effectively than VR mental relaxation (VRMR; a control condition, controlling for time in VR). Standard care will be measured (CSRI; Beecham and Knapp, 1992) but remain as usual in both groups. Assessments will be carried out at 0, 2, 4 (post treatment) and 8, 16, and 24 weeks by a researcher blind to treatment allocation.

2.3 Objectives

The key question to test is: Does VRCB lead to greater reduction in delusional conviction, and improvements in activity levels, quality of life, and suicidal ideation, compared to the control condition (VR mental relaxation; VRMR)?

The primary hypothesis is that VRCB will lead to a reduction in delusional conviction compared to VR mental relaxation.

The secondary hypotheses are:

1. At 4 weeks, VRCB, compared to VRMR, will lead to a reduction in distress in real world situations.

2. At 4 weeks, VRCB, compared to VRMR, will lead to an increase in activity, quality of life, and a reduction in suicide ideation and overall paranoia.

3. VRCB, compared to VRMR, will lead to improvements in delusional severity, wellbeing, and perceptions of recovery.

4. Benefits of VRCB will be maintained over time.

The tertiary hypothesis is that change in delusion conviction will be mediated by changes in safety beliefs and use of defence behaviours.

2.4 Target population

Participants with persistent persecutory delusions in the context of non-affective psychosis.

2.4.1 Inclusion Criteria

- Participant is willing and able to give informed consent for participation in the trial.
- Male or Female, aged 16 years or above.
- Persistent (at least 3 months) persecutory delusion (as defined by Freeman & Garety, 2001), held with at least 50% conviction; specifically, participants will be reporting feeling threatened when with other people.
- Primary diagnosis of schizophrenia-spectrum psychosis (non-affective psychosis).

2.4.2 Exclusion Criteria

The participant may not enter the trial if ANY of the following apply:

- Primary diagnosis of alcohol or substance disorder
- Photosensitive epilepsy
- Significant visual, auditory, or balance impairment

- Current receipt of another psychological therapy
- Insufficient comprehension of English
- In forensic settings
- Organic syndrome
- Learning disability
- Current active suicidal plans

A participant may also not enter the trial if there is another factor, which, in the judgement of the investigator, would preclude the participant from providing informed consent or from safely engaging with the trial procedures. Reason for exclusion will be recorded in line with CONSORT guidelines.

In addition the following stipulation was added due to the COVID-19 pandemic:

- Upon restarting in September 2020 after the suspension of the trial due to COVID-19, recruitment suspension was continued for trial participants who have any of the conditions that would make someone high or moderate risk (clinically vulnerable) for a severe course of COVID-19 (https://www.nhs.uk/conditions/coronavirus-covid-19/people-at-higher-risk/whos-at-higher-risk-from-coronavirus/).

2.5 Interventions

The intervention is more comprehensive, affordable and portable, 'plug and play' version of the VR cognitive treatment: VR Confidence Building (VRCB). In each session, patients were able to choose one of the four available virtual reality sub-scenarios within a coherent shopping centre environment: a café, a lift, a central area, or a clothes shop. This virtual environment represents real life situations, which patients with persecutory delusions may come across in day to day life and often find challenging. In these scenarios, a virtual coach (which speaks and moves as a real person would) encouraged the participant to enter the feared situation (which included virtual people), provide psychoeducation, and encourage them to drop safety behaviours. The VRCB was compared to an alternative active treatment (VR mental relaxation; VRMR).

2.6 Outcomes measures

The table below outlines the objectives, outcome measures and time points of assessment for each outcome:

**Objectives**<br><br>To test the following hypothesis:	**Outcome Measures**	**Timepoint(s) of evaluation of this**

		outcome measure (if applicable)
**Primary hypothesis:** VRCB will lead a reduction in delusional conviction compared to VR mental relaxation.	The primary outcome measure will be conviction in the persecutory delusion (using a 0–100% scale).	0, 2, 4, 8, 16, and 24 weeks (primary end point 4 weeks)
**Secondary Hypotheses:**<br><br>1. At 4 weeks, VRCB, compared to VRMR, will lead to a reduction in distress in real world situations.<br><br>2. At 4 weeks, VRCB, compared to VRMR, will lead to an increase in activity, quality of life, and a reduction in suicide ideation and overall paranoia.<br><br>3. VRCB, compared to VRMR, will lead to improvements in delusional severity, wellbeing, and perceptions of recovery.<br><br>4. Benefits of VRCB will be maintained over time.	1. Real world distress related to the persecutory delusion will be assessed using a behavioural avoidance task (O-BAT; Freeman et al., 2016). Participants create a 5 step hierarchy of real world situations and rate levels of distress (on a scale from 0 (not distressed at all) to 10 (extremely distressed)), resulting in scores of avoidance and distress.<br><br>2. Activity will be assessed by actigraphy and a time-budget measure (Jolley, 2006). Quality of life will be assessed by the EQ-5D-5L (http://www.euroqol.org/); suicide ideation will be assessed by the Columbia-Suicide Severity Rating Scale (Posner et al., 2011); overall paranoia will be assessed by the Revised-GPTS (Green et al., 2008; Freeman et al., 2019)<br><br>3. Delusion severity will be assessed by PSYRATS (Haddock et al, 1999).) Wellbeing will be assessed by the WEMWBS (Tennant et al., 2007), perceptions of recovery will be assessed by the QPR (Neil et al., 2009).<br><br>4. As in 1-3 above	1. 0 and 4 weeks.<br><br>2. 0, 4, and 24 weeks (primary end point 4 weeks)<br><br>3. 0, 4, and 24 weeks (primary end point 4 weeks)<br><br>4. 8, 16, and 24 weeks
**Tertiary Hypothesis:**<br><br>Change in delusion conviction will be mediated by changes in safety	Use of defence behaviours will be assessed using the Safety Behaviours Questionnaire (Freeman et al., 2001;	0, 2, 4, 8, 16, and 24 weeks

beliefs and use of defence behaviours.	strength of safety beliefs will be assessed using a visual analogue scale (Freeman et al., 2016).	(primary end point 4 weeks)

2.7 Sample size

Based on the pilot test against exposure (Freeman et al, 2016), we expect at least a 20% reduction in delusional conviction (effect size=1.0) for VRCB compared to VRMR. This is a conservatively lower effect size expectation than the first pilot, despite the first pilot using a briefer VRCB and a control condition that included one part of active treatment. Nonetheless, we recognise that an even lower effect size (d=0.75, reflecting a 15% reduction in conviction) for delusions would still be of interest to pursue and thus we power the full trial (n=90) on this basis. Following guidelines for good practice (Lancaster et al, 2004), interim analysis provided simple descriptive statistics and an initial estimate of the 95% confidence interval for the treatment effect. This interim analysis of the week 4 data after 30 participants, provided an estimate of conditional power (i.e. power given the data obtained so far - Whitehead & Matsushita, 2003; Snapinn et al., 2006).

We will stop the trial if the interim estimate of effect size, d, is 0.1 or lower, implying that the conditional power of the full trial, based on the interim results and the hypothesised effect size of 0.75, would be 60% or lower. If it assumed that the treatment effect seen in the pilot would continue throughout the rest of the trial, then the conditional power would be as low as 3%.

2.8 Randomisation and blinding in the analysis stage

Randomisation occurred after completion of the baseline assessment. Allocation to VRCB and VRMR was based on a 1:1 allocation ratio. Randomisation was carried out by an online system designed by the University of Oxford Primary Care Clinical Trials Unit. Randomisation using a permuted blocks algorithm, with randomly varying block size, was stratified by severity of delusion (moderate (50-75% conviction)/high (76%+ conviction)).

The trial assessors were blind to group allocation, but the patients and trial therapists were not (they could not be blinded to what psychological treatment was delivered or received). The trial therapists informed patients of the randomisation outcome so that the research assessors remained blind to group allocation. Precautionary strategies to prevent breaks of blind included: the therapist and assessor considering room use and booking arrangements; patients being reminded by the assessor not to talk about treatment allocation and, after the initial assessment, the assessor not looking at the patient’s clinical notes. If an allocation was revealed between assessment sessions, this was logged by the trial coordinator/PI and re-blinding occurred using another assessor.

2.9 Data cleaning

Day to day data management was conducted by Psychiatry team. Additional data checking was also carried out by the statistics team and these files were saved on a restricted folder, "K:\Stats\3. OCHNCTU\THRIVE\10. Data Cleaning".

Two participants reported a higher conviction during screening and were deemed eligible and then gave a lower conviction rating during their baseline assessment. Due to this, there is a discrepancy between the two measures so that the continuous conviction rating has a lower bound of 25, while the binary variable severity of delusion is categorised as moderate (50-75% conviction)/high (76%+ conviction).

2.10 Analysis for Data Monitoring and ethics Committee meetings

A Data Monitoring and Ethics Committee (DMEC) was formed with an independent clinician chair, independent statistician, and further independent clinician, to monitor the safety and progress of the trial. All details of analysis for DMC meetings are stored on a restricted folder, "K:\Stats\3. OCHNCTU\THRIVE\4. TSC and DMC\DMC".

2.11 Definition of population for analysis

The primary statistical analysis of efficacy outcomes will be carried out on the full analysis dataset. That is, after randomisation, participants will be analysed according to their allocated intervention group irrespective of what intervention they actually receive. Every effort will be made for full follow-up data on every participant to allow for as complete as possible analysis. If follow up data is missing for some participants, then any available data for those participants will be included in the primary analysis, under the missing at random (MAR) assumption.

2.12 Deviation from SAP

- The SAP specifies that the primary outcome analysis adjusts for severity of delusion as well as baseline conviction score. However, as the former is derived from the latter, it was decided that just the (continuous) baseline conviction score would be included in the analysis to avoid over-adjustment.
- For the analysis of the secondary outcomes O-BAT Avoidance and the C-SSRS Suicidal Ideation, the models were fitted with categorical baseline measures rather than continuous ones as the outcomes have discrete categories.

- The SAP stated that for the EQ-5D-5L measure, the p-values and confidence intervals for the model coefficients would be obtained by means of bootstrapping – this was not done as the models diagnostics showed that the model assumptions were not violated

3 Results

3.1 Representativeness of Study Sample and Patient Throughput

The diagram below shows the flow of participants through the trial. For each time point, 'Reached' is based on time from randomisation while 'Completed' implies that the primary outcome assessment was carried out and a persecutory belief conviction score is available for analysis.

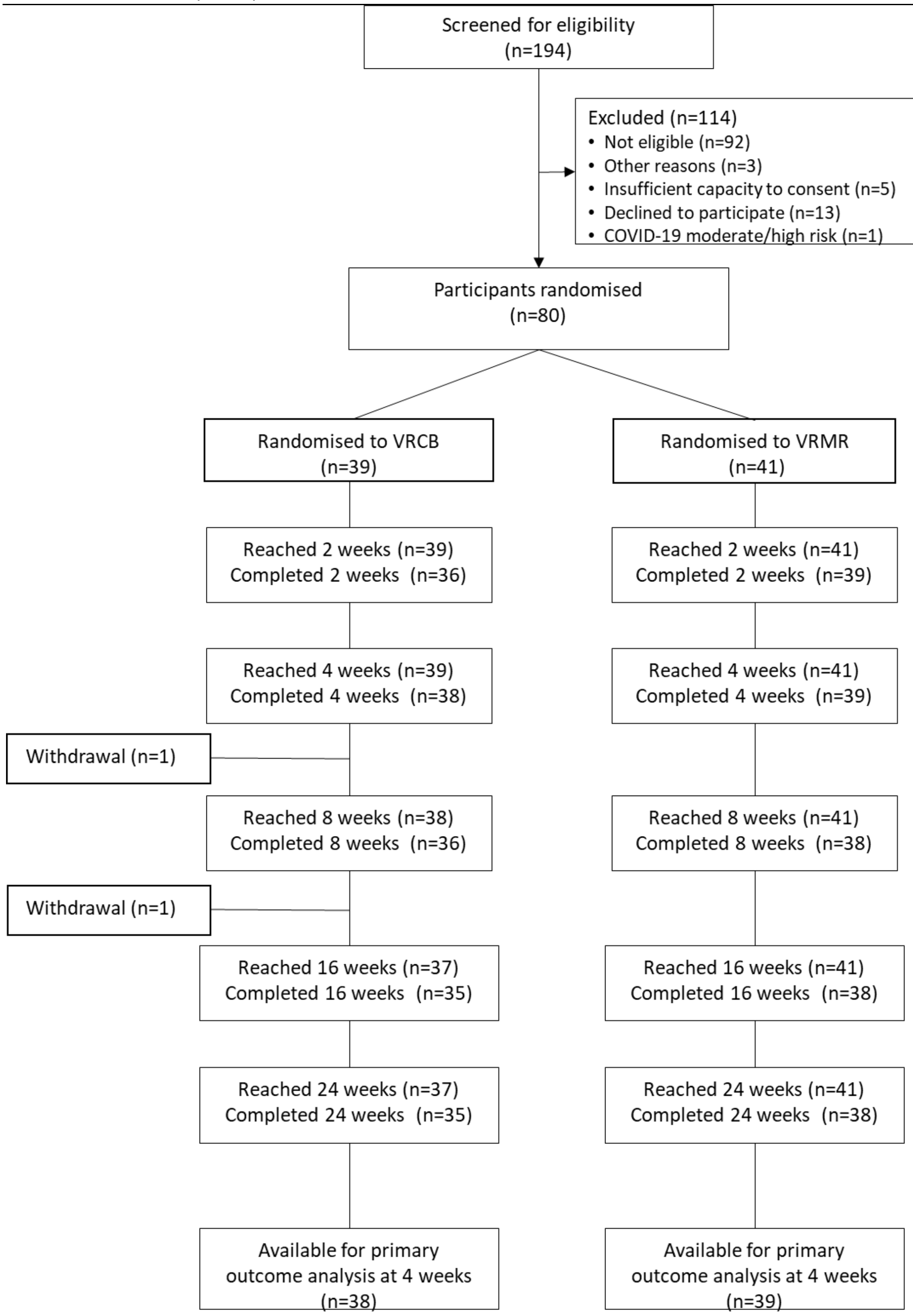

FIGURE 2: PARTICIPANT FLOW DIAGRAM

3.2 Recruitment

The first participant was randomised on 21 September 2018. The trial closed to recruitment on 13/05/2021 with 80 participants randomized.

The trial was not stopped early.

3.3 Baseline characteristics of participants

The table below summarises the baseline characteristic for the participants overall as well as separately for the two treatment groups. In general, the groups appear to be balanced.

TABLE 2: BASELINE CHARACTERISTICS BY RANDOMISED GROUP

	VRCB (N=39)	VRMR (N=41)	Total (N=80)
**Age (years)**			
Mean (SD)	41.1 (13.4)	39.4 (13.0)	40.3 (13.1)
Median (IQR)	41.4 (30.3 to 50.1)	37.6 (30.0 to 47.2)	39.1 (30.1 to 49.0)
Min to Max	18.9 to 70.8	18.9 to 72.7	18.9 to 72.7
**Sex, n (%)**			
Male	25 (64.1)	24 (58.5)	49 (61.3)
Female	14 (35.9)	17 (41.5)	31 (38.8)
**Current Marital status, n (%)**			
Single	26 (66.7)	31 (75.6)	57 (71.3)
Cohabiting	3 (7.7)	3 (7.3)	6 (7.5)
Married or Civil Partnership	7 (17.9)	5 (12.2)	12 (15.0)
Divorced	3 (7.7)	2 (4.9)	5 (6.3)
Widowed	-	-	-
**Ethnic group, n (%)**			
White	28 (71.8)	36 (87.8)	64 (80.0)
Black Caribbean	3 (7.7)	0	3 (3.8)
Black African	2 (5.1)	0	2 (2.5)
Black other	1 (2.6)	0	1 (1.3)
Indian	1 (2.6)	0	1 (1.3)
Pakistani	2 (5.1)	1 (2.4)	3 (3.8)
Chinese	1 (2.6)	0	1 (1.3)
Other	1 (2.6)	4 (9.8)	5 (6.3)
**Employment, n (%)**			
Unemployed	32 (82.1)	36 (87.8)	68 (85.0)
Employed FT	1 (2.6)	-	1 (1.3)
Employed PT	3 (7.7)	1 (2.4)	4 (5.0)
Self Employed	1 (2.6)	1 (2.4)	2 (2.5)
Retired	2 (5.1)	2 (4.9)	4 (5.0)
Student	-	-	-

	VRCB (N=39)	VRMR (N=41)	Total (N=80)
Housewife / Husband	-	1 (2.4)	1 (1.3)
**Usual/Normal living arrangement, n (%)**			
Living alone +- children	15 (38.5)	17 (41.5)	32 (40.0)
Living with husband/wife +- children	8 (20.5)	5 (12.2)	13 (16.3)
Living together as a couple	2 (5.1)	2 (4.9)	4 (5.0)
Living with parents	7 (17.9)	12 (29.3)	19 (23.8)
Living with other relatives	3 (7.7)	1 (2.4)	4 (5.0)
Living with others	4 (10.3)	4 (9.8)	8 (10.0)
**Total number of mental health admissions in the last 24 weeks (BL), n (%)**			
0	37 (94.9)	36 (87.8)	73 (91.3)
1	2 (5.1)	4 (9.8)	6 (7.5)
2	-	1 (2.4)	1 (1.3)
**Type of medications in use[2]**			
Antipsychotic, n (%)			
No	*1 (2.6)*	*4 (9.8)*	*5 (6.3)*
Yes	*38 (97.4)*	*37 (90.2)*	*75 (93.8)*
Antidepressant, n (%)			
No	*11 (28.2)*	*15 (36.6)*	*26 (32.5)*
Yes	*28 (71.8)*	*26 (63.4)*	*54 (67.5)*
Anxiolytic, n (%)			
No	*34 (87.2)*	*35 (85.4)*	*69 (86.3)*
Yes	*5 (12.8)*	*6 (14.6)*	*11 (13.8)*
Mood stabiliser, n (%)			
No	*31 (79.5)*	*33 (80.5)*	*64 (80.0)*
Yes	*8 (20.5)*	*8 (19.5)*	*16 (20.0)*
Hypnotic, n (%)			
No	*37 (94.9)*	*40 (97.6)*	*77 (96.3)*
Yes	*2 (5.1)*	*1 (2.4)*	*3 (3.8)*
Stimulant, n (%)			
No	*39 (100.0)*	*41 (100.0)*	*80 (100.0)*
**Severity of delusion[1], n (%)**			
Moderate	18 (46.2)	18 (43.9)	36 (45.0)
High	21 (53.8)	23 (56.1)	44 (55.0)
**Persecutory belief conviction at baseline (primary outcome)**			
Mean (SD)	78.1 (18.1)	79.0 (15.5)	78.6 (16.7)
Median (IQR)	80.0 (70.0 to 90.0)	80.0 (70.0 to 90.0)	80.0 (70.0 to 90.0)
Min to Max	25.0 to 100.0	35.0 to 100.0	25.0 to 100.0
Missing	-	-	-
**O-BAT - maximum number of steps avoided**			
Mean (SD)	2.5 (1.4)	2.3 (1.6)	2.4 (1.5)
Median (IQR)	3.0 (2.0 to 3.0)	2.0 (1.0 to 4.0)	3.0 (1.5 to 3.0)
Min to Max	0.0 to 5.0	0.0 to 5.0	0.0 to 5.0
Missing	9	15	24

	VRCB (N=39)	VRMR (N=41)	Total (N=80)
**O-BAT Mean distress score**			
Mean (SD)	5.2 (2.2)	6.0 (1.8)	5.6 (2.1)
Median (IQR)	5.5 (3.8 to 7.0)	6.0 (5.0 to 7.3)	5.7 (4.4 to 7.0)
Min to Max	0.3 to 8.9	2.0 to 9.5	0.3 to 9.5
Missing	11	17	28
**(Actigraphy) Mean number of steps (daily)**			
Mean (SD)	5060.9 (3509.4)	5038.7 (4137.4)	5052.7 (3708.0)
Median (IQR)	3945.3 (2692.7 to 6355.1)	4178.7 (2767.1 to 6144.4)	4062.0 (2692.7 to 6355.1)
Min to Max	523.3 to 14322.3	567.7 to 18086.4	523.3 to 18086.4
Missing	10	24	34
**Time budget score**			
Mean (SD)	49.3 (15.4)	47.7 (13.3)	48.5 (14.3)
Median (IQR)	46.0 (39.0 to 57.0)	47.0 (38.5 to 59.0)	47.0 (39.0 to 59.0)
Min to Max	25.0 to 83.0	22.0 to 72.0	22.0 to 83.0
Missing	4	5	9
**EQ-5D-5L INDEX**			
Mean (SD)	0.5 (0.3)	0.5 (0.2)	0.5 (0.3)
Median (IQR)	0.6 (0.4 to 0.7)	0.6 (0.3 to 0.7)	0.6 (0.4 to 0.7)
Min to Max	-0.4 to 1.0	0.0 to 1.0	-0.4 to 1.0
Missing	3	3	6
**EQ5D VAS score**			
Mean (SD)	51.9 (19.7)	42.7 (23.3)	47.1 (22.0)
Median (IQR)	50.0 (37.5 to 62.0)	50.0 (27.0 to 60.0)	50.0 (30.0 to 60.0)
Min to Max	10.0 to 90.0	0.0 to 90.0	0.0 to 90.0
Missing	3	3	6
**Columbia Suicide Severity Rating Scale (C-SSRS) total score**			
Mean (SD)	0.7 (1.0)	0.9 (1.2)	0.8 (1.1)
Median (IQR)	0.0 (0.0 to 1.0)	0.0 (0.0 to 1.0)	0.0 (0.0 to 1.0)
Min to Max	0.0 to 3.0	0.0 to 4.0	0.0 to 4.0
Missing	2	4	6
**R-GPTS-A (social reference) score**			
Mean (SD)	15.1 (8.0)	15.5 (7.4)	15.3 (7.7)
Median (IQR)	14.0 (10.0 to 20.0)	16.0 (11.0 to 20.0)	14.5 (10.0 to 20.0)
Min to Max	2.0 to 32.0	0.0 to 32.0	0.0 to 32.0
Missing	-	-	-
**R-GPTS-B (persecution) score**			
Mean (SD)	23.7 (8.1)	21.3 (8.8)	22.5 (8.5)
Median (IQR)	23.0 (17.0 to 31.0)	20.0 (16.0 to 27.0)	23.0 (16.0 to 28.5)
Min to Max	7.0 to 40.0	1.0 to 38.0	1.0 to 40.0
Missing	-	-	-
**R-GPTS (overall) score**			
Mean (SD)	38.8 (15.1)	36.8 (14.6)	37.8 (14.8)
Median (IQR)	37.0 (27.0 to 52.0)	37.0 (28.0 to 46.0)	37.0 (27.5 to 47.5)

	VRCB (N=39)	VRMR (N=41)	Total (N=80)
Min to Max	10.0 to 72.0	1.0 to 68.0	1.0 to 72.0
Missing	-	-	-
**Delusion severity (PSYRATS)**			
Mean (SD)	16.9 (3.2)	16.9 (3.5)	16.9 (3.3)
Median (IQR)	17.0 (15.0 to 19.0)	16.0 (15.0 to 19.0)	17.0 (15.0 to 19.0)
Min to Max	9.0 to 23.0	9.0 to 24.0	9.0 to 24.0
Missing	-	-	-
**Wellbeing (WEMWBS)**			
Mean (SD)	33.7 (8.9)	34.6 (8.6)	34.1 (8.7)
Median (IQR)	35.0 (28.0 to 40.0)	36.0 (29.0 to 41.0)	35.0 (28.0 to 41.0)
Min to Max	14.0 to 52.0	15.0 to 52.0	14.0 to 52.0
Missing	1	4	5
**Process of recovery (QPR) total score**			
Mean (SD)	25.1 (10.4)	25.4 (10.0)	25.3 (10.1)
Median (IQR)	27.9 (18.0 to 33.0)	26.0 (19.0 to 32.0)	27.0 (18.0 to 32.0)
Min to Max	3.0 to 41.0	2.0 to 46.0	2.0 to 46.0
Missing	2	-	2
**Total frequency of safety behaviours at baseline**			
Mean (SD)	16.8 (10.8)	21.4 (11.0)	19.1 (11.1)
Median (IQR)	16.5 (9.0 to 25.0)	19.0 (15.0 to 27.0)	18.0 (10.0 to 25.0)
Min to Max	1.0 to 48.0	1.0 to 52.0	1.0 to 52.0
Missing	1	4	5
**Safety belief conviction at baseline**			
Mean (SD)	36.9 (26.2)	40.5 (23.3)	38.7 (24.7)
Median (IQR)	30.0 (20.0 to 50.0)	40.0 (25.0 to 50.0)	40.0 (25.0 to 50.0)
Min to Max	0.0 to 100.0	0.0 to 100.0	0.0 to 100.0
Missing	-	1	1

[1] Stratification variable

[2] Not mutually exclusive

3.4 Number analysed

The table below summarises the number of participants analysed at the 4 and 24 week time points. Numbers for the other time points can be obtained from the CONSORT flow diagram.

TABLE 3 COMPLETION OF FOLLOW-UP ASSESSMENTS, WITHDRAWALS, AND LOST TO FOLLOW-UP OVER THE STUDY PERIOD

	VRCB	VRMR	Overall
**Randomised, n**	39	41	80
**4-week primary outcome score available, n (%)**	38 (97.4)	39 (95.1)	77 (96.3)
**24-week primary outcome score available, n (%)**	35 (89.7)	38 (92.7)	73 (91.3)

	VRCB	VRMR	Overall
**Withdrawn after randomisation, before 4 week follow-up, n (%)**	0	0	0
**Lost to follow-up before 4 week follow-up, n (%)**	0	0	0
**Withdrawn after 4 weeks but before 24 week follow-up, n (%)**	2 (5.1)	0	2 (2.5)
**Lost to follow-up after 4 weeks but before 24 week follow-up, n (%)**	0	0	0

3.5 Primary Analyses

3.5.1 Predictors of missing primary outcome data

Table 3 shows the association between randomised group and availability of the primary outcome. Here, 1 (2.6%) participant in VRCB and 2 (4.9%) in VRMR did not have data to determine the primary outcome. The results show that there is no association between randomised group and availability of either of the primary outcomes (p value = 0.592).

TABLE 4: ASSOCIATION BETWEEN RANDOMISED GROUP AND AVAILABILITY OF PRIMARY OUTCOME AT 4 WEEKS

	VRCB (N=39)	VRMR (N=41)	Odds ratio (95% CI)[1]	P value
**Persecutory belief conviction**				
**Missing, n (%)**	1 (2.6%)	2 (4.9%)	0.51 [0.04 to 5.90]	0.592
**Available, n (%)**	38 (97.4%)	39 (95.1%)		

[1] Logistic regression of the availability of the primary outcome for VRCB versus VRMR. Level of significance = 0.05

In Table 4 below, baseline characteristics are summarised for those participants with a missing primary outcome in order to establish whether they differ from the main cohort. Frequencies and percentages for the binary/categorical, and summary statistics for the continuous baseline characteristics are presented; split by randomised group and if the participants had the primary outcome or not. Associated P-values for predicting missingness of the primary outcome (randomised groups combined) were obtained from individual logistic regression models for each baseline characteristics.

For each of Ethnic group, number of mental health admissions, Anxiolytic use & EQ-5D-5L Index there appears to be association between missingness of the primary outcome and randomised treatment group. These baseline measures are included in the sensitivity analysis investigating the effect of missingness on the primary outcome analysis (section 3.7).

TABLE 5: BASELINE CHARACTERISTICS OF PARTICIPANTS BY COMPLETENESS OF PERSECUTORY BELIEF CONVICTION AT 4 WEEKS

Baseline Characteristic	Predictive of missingness (P Value)[1]	VRCB (N=39)		VRMR (N=41)	
		Missing (n=1)	Not missing (n=38)	Missing (n=2)	Not missing (n=39)
**Age (years)**	0.161				
Mean (SD)		32.6	41.4 (13.5)	27.4 (5.7)	40.0 (13.0)
Median (IQR)		32.6 (32.6 to 32.6)	41.5 (30.3 to 50.1)	27.4 (23.3 to 31.4)	38.5 (30.0 to 47.6)
Min to Max		32.6 to 32.6	18.9 to 70.8	23.3 to 31.4	18.9 to 72.7
**Sex, n (%)**	0.845				
Male		1/1 (100.0)	24/38 (63.2)	1/2 (50.0)	23/39 (59.0)
Female		-	14/38 (36.8)	1/2 (50.0)	16/39 (41.0)
**Current Marital status, n (%)**	0.679				
Single		-	26/38 (68.4)	2/2 (100.0)	29/39 (74.4)

Baseline Characteristic	Predictive of missingness (P Value)[1]	VRCB (N=39)		VRMR (N=41)	
		Missing (n=1)	Not missing (n=38)	Missing (n=2)	Not missing (n=39)
Cohabiting		1/1 (100.0)	2/38 (5.3)	-	3/39 (7.7)
Married or Civil Partnership		-	7/38 (18.4)	-	5/39 (12.8)
Divorced		-	3/38 (7.9)	-	2/39 (5.1)
**Ethnic group, n (%)**	0.030				
White		1/1 (100.0)	27/38 (71.1)	-	36/39 (92.3)
Black Caribbean		-	3/38 (7.9)	-	-
Black African		-	2/38 (5.3)	-	-
Black other		-	1/38 (2.6)	-	-
Indian		-	1/38 (2.6)	-	-
Pakistani		-	2/38 (5.3)	1/2 (50.0)	-
Chinese		-	1/38 (2.6)	-	-
Other		-	1/38 (2.6)	1/2 (50.0)	3/39 (7.7)
**Employment, n (%)**	0.453				
Unemployed, n (%)		1/1 (100.0)	31/38 (81.6)	1/2 (50.0)	35/39 (89.7)
Employed FT, n (%)		-	1/38 (2.6)	-	-
Employed PT		-	3/38 (7.9)	-	1/39 (2.6)
Self Employed		-	1/38 (2.6)	1/2 (50.0)	-
Retired		-	2/38 (5.3)	-	2/39 (5.1)
Student		-	-	-	-
Housewife / Husband		-	-	-	1/39 (2.6)
**Usual/Normal living arrangement, n (%)**	0.509				
Living alone +- children		-	15/38 (39.5)	-	17/39 (43.6)
Living with husband/wife +-children		1/1 (100.0)	7/38 (18.4)	-	5/39 (12.8)
Living together as a couple		-	2/38 (5.3)	-	2/39 (5.1)
Living with parents		-	7/38 (18.4)	2/2 (100.0)	10/39 (25.6)
Living with other relatives		-	3/38 (7.9)	-	1/39 (2.6)
Living with others		-	4/38 (10.5)	-	4/39 (10.3)
**Mental health admissions, n (%)**	0.031				
1		1/1 (100.0)	36/38 (94.7)	-	36/39 (92.3)
2		-	2/38 (5.3)	2/2 (100.0)	2/39 (5.1)
3		-	-	-	1/39 (2.6)
**Type of medications in use[2]**					
**Antipsychotic**, n (%)	-				
No		-	1/38 (2.6)	-	4/39 (10.3)
Yes		1/1 (100.0)	37/38 (97.4)	2/2 (100.0)	35/39 (89.7)
**Antidepressant**, n (%)	0.234				
No		1/1 (100.0)	10/38 (26.3)	1/2 (50.0)	14/39 (35.9)
Yes		-	28/38 (73.7)	-	25/39 (64.1)

Baseline Characteristic	Predictive of missingness (P Value)[1]	VRCB (N=39)		VRMR (N=41)	
		Missing (n=1)	Not missing (n=38)	Missing (n=2)	Not missing (n=39)
**Anxiolytic**, n (%)	0.033				
No		-	34/38 (89.5)	-	34/39 (87.2)
Yes		1/1 (100.0)	4/38 (10.5)	1/2 (50.0)	5/39 (12.8)
**Mood stabiliser**, n (%)	0.564				
No		1/1 (100.0)	30/38 (78.9)	1/2 (50.0)	32/39 (82.1)
Yes		-	8/38 (21.1)	-	7/39 (17.9)
**Hypnotic**, n (%)	-				
No		1/1 (100.0)	36/38 (94.7)	2/2 (100.0)	38/39 (97.4)
Yes		-	2/38 (5.3)	-	1/39 (2.6)
**Stimulant**, n (%)	-				
No		1/1 (100.0)	38/38 (100.0)	2/2 (100.0)	39/39 (100.0)
Yes		-	-	-	-
**Primary & Secondary outcomes at baseline, mean (sd) [n]**					
**Persecutory belief conviction at baseline (primary outcome)**	0.978	100.0 [1]	77.6 (18.0) [38]	67.5 (46.0) [2]	79.6 (13.8) [39]
**O-BAT - maximum number of steps avoided**	0.930	3.0 [1]	2.5 (1.4) [29]	2.0 [1]	2.3 (1.6) [25]
**O-BAT Mean distress score**	0.991	4.5 [1]	5.2 (2.2) [27]	6.7 [1]	6.0 (1.8) [23]
**(Actigraphy) Mean number of steps (daily)**	0.060	3791.2 [1]	5106.2 (3565.1) [28]	18086.4 [1]	4223.2 (2490.0) [16]
**Time budget score**	0.431	46.0 [1]	49.4 (15.6) [34]	35.0 [1]	48.1 (13.3) [35]
**EQ-5D-5L INDEX**	0.043	0.1 [1]	0.5 (0.3) [35]	0.0 [1]	0.6 (0.2) [37]
**EQ5D VAS score**	0.447	-	51.9 (19.7) [36]	30.0 [1]	43.0 (23.5) [37]
**Columbia Suicide Severity Rating Scale (C-SSRS) total score**	0.167	3.0 [1]	0.7 (0.9) [36]	1.0 [1]	0.9 (1.3) [36]
**R-GPTS-A (social reference) score**	0.821	32.0 [1]	14.7 (7.6) [38]	5.5 (0.7) [2]	16.0 (7.2) [39]
**R-GPTS-B (persecution) score**	0.970	31.0 [1]	23.5 (8.1) [38]	18.5 (12.0) [2]	21.5 (8.8) [39]
**R-GPTS (overall) score**	0.924	63.0 [1]	38.2 (14.8) [38]	24.0 (11.3) [2]	37.5 (14.5) [39]
**Delusion severity (PSYRATS)**	0.353	23.0 [1]	16.8 (3.0) [38]	16.5 (9.2) [2]	16.9 (3.3) [39]
**Wellbeing (WEMWBS)**	0.406	31.0 [1]	33.8 (9.0) [37]	27.0 [1]	34.8 (8.6) [36]
**Process of recovery (QPR) total score**	0.777	9.0 [1]	25.6 (10.2) [36]	31.0 (21.2) [2]	25.1 (9.5) [39]

Baseline Characteristic	Predictive of missingness (P Value)[1]	VRCB (N=39)		VRMR (N=41)	
		Missing (n=1)	Not missing (n=38)	Missing (n=2)	Not missing (n=39)
**Total frequency of safety behaviours at baseline**	0.073	35.0 [1]	16.3 (10.6) [37]	34.0 [1]	21.0 (10.9) [36]
**Safety belief conviction at baseline**	0.569	45.0 [1]	36.7 (26.5) [38]	47.5 (31.8) [2]	40.1 (23.3) [38]

[1] P value obtained from a logistic regression of missingness of primary outcome against each of the baseline characteristics (randomised groups combined). Level of significance = 0.05

[2] Not mutually exclusive

3.5.2 Primary & Secondary Outcomes Analyses

The primary objective was to compare the effect of treatment on conviction in the persecutory delusion (using a 0-100% scale) at 4 weeks for the primary outcome and at later time points for determining the long-term effect.

Table 4 presents the results from the primary and secondary outcomes analyses. Regression diagnostic plots for these models are in ***Appendix I.*** *Diagnostic plots.*

For the primary outcome, the results show that there is no evidence of a difference in conviction in the persecutory delusion in VRMR compared to VRCB at any of the time points.

There is no evidence of any treatment differences for any of the secondary outcomes at any time point.

TABLE 6: SUMMARY STATISTICS FOR THE PRIMARY AND SECONDARY OUTCOMES AND THE TREATMENT DIFFERENCE BETWEEN THE RANDOMISED GROUPS

	VRCB (N=39)	VRMR (N=41)	Adjusted mean difference [95% CI][1]	P value
**Primary outcome**				
**Persecutory belief conviction (primary outcome), mean (SD) [n]**				
2 weeks	62.4 (23.9) [36]	65.2 (19.3) [39]	-0.94 [-11.63 to 9.75]; Std. effect: -0.06 [-0.70 to 0.58]	0.863
4 weeks	52.1 (23.8) [38]	56.6 (25.0) [39]	-2.16 [-12.77 to 8.44]; Std. effect: -0.13 [-0.76 to 0.50]	0.689
8 weeks	53.7 (25.0) [36]	55.7 (26.6) [38]	0.93 [-9.80 to 11.66]; Std. effect: 0.06 [-0.59 to 0.70]	0.865
16 weeks	53.3 (23.5) [35]	49.7 (28.9) [38]	5.78 [-5.00 to 16.56]; Std. effect: 0.35 [-0.30 to 0.99]	0.294
24 weeks	51.9 (27.4) [35]	53.0 (29.0) [38]	-1.37 [-12.12 to 9.39]; Std. effect: -0.08 [-0.72 to 0.56]	0.803
**Secondary outcomes**				
**O-BAT - maximum number of steps avoided[2], mean (SD) [n]**				
2 weeks	-	-		
4 weeks	2.4 (1.9) [22]	1.6 (1.8) [21]	0.83 [-0.19 to 1.85]	0.107
8 weeks	-	-		
16 weeks	-	-		
24 weeks	-	-		
**O-BAT Mean distress score, mean (SD) [n]**				
2 weeks	-	-		
4 weeks	4.5 (2.5) [17]	4.6 (1.9) [18]	0.06 [-1.33 to 1.45]	0.932
8 weeks	-	-		
16 weeks	-	-		
24 weeks	-	-		
**(Actigraphy) Mean number of steps (daily)[3], mean (SD) [n]**				
2 weeks	-	-		
4 weeks	4511.9 (3448.8) [19]	4239.4 (4187.8) [17]	418.3 [-1088.6 to 1925.2]	0.586

	VRCB (N=39)	VRMR (N=41)	Adjusted mean difference [95% CI][1]	P value
8 weeks	-	-		
16 weeks	-	-		
24 weeks	5827.3 (4765.5) [10]	3138.1 (3287.1) [12]	-121.7 [-1800.5 to 1557.2]	0.887
**Time budget score[3], mean (SD) [n]**				
2 weeks	-	-		
4 weeks	48.9 (16.7) [29]	46.9 (14.2) [34]	1.83 [-4.33 to 7.98]	0.561
8 weeks	-	-		
16 weeks	-	-		
24 weeks	49.0 (17.5) [25]	49.6 (14.7) [29]	-1.94 [-8.52 to 4.64]	0.563
**EQ-5D-5L INDEX, mean (SD) [n]**				
2 weeks	-	-		
4 weeks	0.6 (0.3) [36]	0.6 (0.3) [37]	0.01 [-0.09 to 0.11]	0.887
8 weeks	-	-		
16 weeks	-	-		
24 weeks	0.6 (0.3) [32]	0.5 (0.3) [34]	0.04 [-0.06 to 0.14]	0.427
**EQ5D VAS score[3], mean (SD) [n]**				
2 weeks	-	-		
4 weeks	58.1 (20.5) [36]	54.0 (23.9) [37]	-0.75 [-9.24 to 7.75]	0.863
8 weeks	-	-		
16 weeks	-	-		
24 weeks	58.8 (23.8) [32]	52.0 (20.3) [33]	0.85 [-8.17 to 9.88]	0.853
**Columbia Suicide Severity Rating Scale (C-SSRS) total score[2], mean (SD) [n]**				
2 weeks	-	-		
4 weeks	0.5 (1.0) [35]	0.5 (0.9) [35]	0.12 [-0.29 to 0.52]	0.566
8 weeks	-	-		
16 weeks	-	-		
24 weeks	0.6 (1.2) [31]	0.8 (1.2) [32]	-0.22 [-0.85 to 0.41]	0.495
**R-GPTS-A (social reference) score, mean (SD) [n]**				
2 weeks	14.2 (7.7) [36]	12.7 (6.8) [39]	2.11 [-0.75 to 4.96]	0.149
4 weeks	11.3 (7.5) [37]	11.7 (7.6) [39]	0.55 [-2.31 to 3.40]	0.707
8 weeks	11.7 (7.5) [36]	11.4 (7.7) [38]	1.08 [-1.79 to 3.96]	0.460
16 weeks	10.0 (7.8) [34]	10.7 (7.8) [38]	0.38 [-2.52 to 3.27]	0.799
24 weeks	10.6 (8.8) [34]	11.4 (8.1) [37]	-0.66 [-3.56 to 2.24]	0.657
**R-GPTS-B (persecution) score, mean (SD) [n]**				
2 weeks	20.9 (9.7) [36]	18.0 (9.4) [39]	2.16 [-1.82 to 6.14]	0.287
4 weeks	16.5 (9.8) [37]	16.0 (10.1) [39]	-0.51 [-4.48 to 3.46]	0.802
8 weeks	17.1 (9.7) [36]	15.0 (10.2) [38]	1.17 [-2.83 to 5.17]	0.567
16 weeks	14.1 (10.5) [34]	14.3 (11.7) [38]	-0.98 [-5.01 to 3.05]	0.634
24 weeks	15.1 (13.1) [34]	14.9 (10.8) [37]	-1.60 [-5.64 to 2.45]	0.439
**R-GPTS (overall) score, mean (SD) [n]**				
2 weeks	35.1 (16.7) [36]	30.7 (14.7) [39]	4.38 [-2.01 to 10.76]	0.179
4 weeks	27.9 (16.1) [37]	27.8 (16.5) [39]	0.17 [-6.20 to 6.55]	0.958
8 weeks	28.8 (16.0) [36]	26.4 (16.2) [38]	2.39 [-4.02 to 8.81]	0.465
16 weeks	24.2 (17.6) [34]	25.0 (18.4) [38]	-0.48 [-6.94 to 5.98]	0.885
24 weeks	25.8 (21.3) [34]	26.3 (18.2) [37]	-2.15 [-8.63 to 4.32]	0.515
**Delusion severity (PSYRATS), mean (SD) [n]**				

	VRCB (N=39)	VRMR (N=41)	Adjusted mean difference [95% CI][1]	P value
2 weeks	14.4 (4.2) [36]	15.6 (3.8) [39]	-0.91 [-2.74 to 0.92]	0.332
4 weeks	12.9 (4.4) [37]	13.8 (4.6) [38]	-0.73 [-2.57 to 1.10]	0.432
8 weeks	13.0 (4.1) [36]	13.9 (4.4) [38]	-0.68 [-2.52 to 1.16]	0.467
16 weeks	12.6 (4.5) [35]	13.0 (5.4) [38]	-0.26 [-2.11 to 1.58]	0.779
24 weeks	12.4 (5.9) [34]	13.0 (5.5) [36]	-0.99 [-2.86 to 0.88]	0.300
**Wellbeing (WEMWBS)[3], mean (SD) [n]**				
2 weeks	-	-		
4 weeks	38.6 (10.4) [36]	38.7 (10.1) [38]	0.27 [-2.97 to 3.50]	0.871
8 weeks	-	-		
16 weeks	-	-		
24 weeks	38.9 (10.7) [32]	38.1 (9.9) [33]	0.96 [-2.42 to 4.34]	0.579
**Process of recovery (QPR) total score[3], mean (SD) [n]**				
2 weeks	-	-		
4 weeks	28.7 (11.9) [36]	30.1 (11.1) [39]	-1.27 [-5.18 to 2.64]	0.523
8 weeks	-	-		
16 weeks	-	-		
24 weeks	31.5 (11.8) [33]	29.7 (12.0) [36]	2.29 [-1.74 to 6.31]	0.265

[1] Primary outcome: VRCB versus VRMR: Mean difference estimated from a linear mixed-effects model adjusting for baseline conviction as a fixed effect and participant as the random effect. Standardised effect size = estimated mean difference divided by baseline standard deviation. Secondary outcomes: VRCB versus VRMR: Mean difference estimated from a linear mixed-effects model adjusting for severity of delusion (binary) and baseline values of the outcome as fixed effects and participant as the random effect for all outcomes aside from the O-BAT outcomes which were fitted using linear regression. Estimates and CI for C-SSRS were obtained using bootstrapping as the distribution of the outcome and residuals were skew (see Figure 9).
[2] Baseline values of the outcomes are modelled as a categorical rather than continuous measures.
[3] Positively framed secondary outcomes: positive mean differences indicate that, on average, VRCB is doing better than VRMR.
Level of significance = 0.05

3.6 Tertiary (Mediation) Analyses

The mediation analyses were initially carried out using structural equation modelling (SEM); however, none of the models converged and thus did not produce reliable estimates. As a result, the analyses were run using the approach of Baron and Kenny (1986), but follow the adaptation in Freeman et al. (2017) which makes use of linear mixed effects models, as specified in the SAP.

A representation of the mediation analysis following the Baron and Kenny (1986) approach of the tested pathways is shown below.

The amount of mediation is called the indirect effect.

Total effect (c) = Direct effect (c') + Indirect effect (ab)

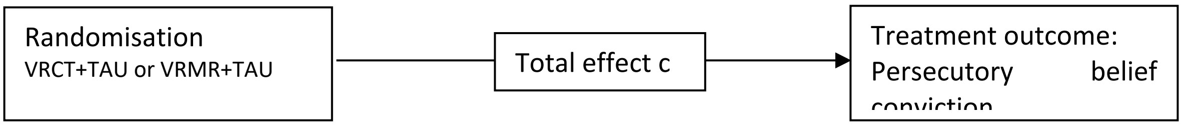
Randomisation
VRCT+TAU or VRMR+TAU
Total effect c
Treatment outcome: Persecutory belief conviction

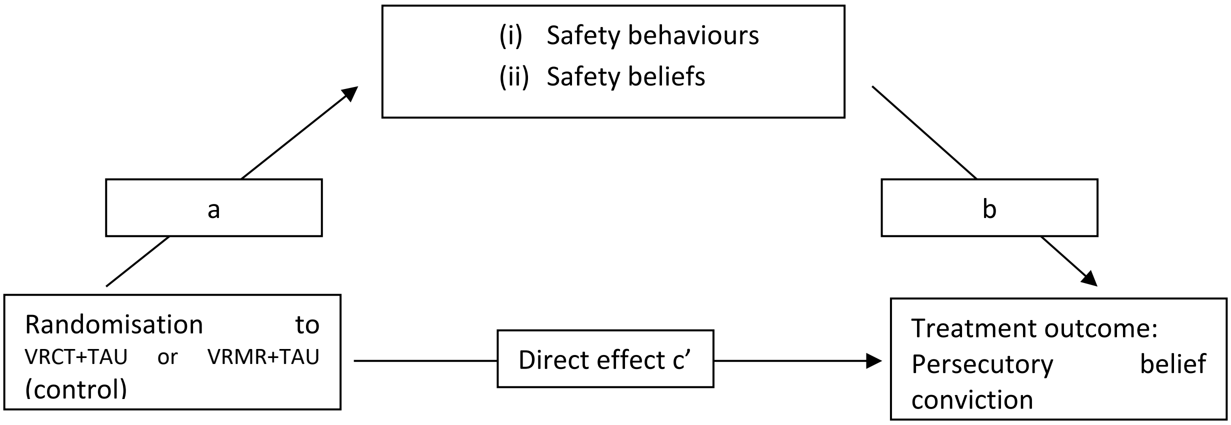
(i) Safety behaviours
(ii) Safety beliefs
a
b
Randomisation to VRCT+TAU or VRMR+TAU (control)
Direct effect c'
Treatment outcome: Persecutory belief conviction

Table 6 shows the mediation results for the primary outcome Persecutory belief conviction. For each of the mediators (Safety behaviours & Safety beliefs), the table shows the total (c), direct (c') and indirect (a+b) effects of treatment group on the outcome.
The indirect effects represent the estimated mediated effects on persecutory belief conviction.

The results show that for both safety behaviours (higher scores indicate worse outcomes) and safely beliefs (higher scores indicate better outcomes), there is no evidence of a direct effect or indirect/mediated effect at the either time point. In other words, there is no evidence of a statistically significant difference between the treatment groups on the primary outcome (direct effect), nor is there evidence of a mediated effect of treatment group on the primary outcome (indirect effect). There is also no difference at any time point between the treatment groups on the mediators (path a in the table below).

TABLE 7: MEDIATION ANALYSIS ON PERSECUTORY BELIEF CONVICTION USING MIXED-EFFECTS MODELS

Mediator	Effect estimate	2-weeks (mid-treatment): Estimate (95% CI); P value	4-weeks (end of treatment): Estimate (95% CI); P value
Safety Behaviours	Total effect	-1.61 (-12.66 to 9.45); P = 0.776	-0.07 (-11.03 to 10.89); P = 0.990
	Direct effect	-0.66 (-11.04 to 9.72); P = 0.901	2.27 (-8.10 to 12.65); P = 0.667
	Indirect effect	0.51 (-2.06 to 3.08); P = 0.696	-0.52 (-3.09 to 2.05); P = 0.692
	a path	0.70 (-2.80 to 4.20); P = 0.695	-0.71 (-4.21 to 2.79); P = 0.691
	b path	0.73 (0.42 to 1.04); P = <0.001	0.73 (0.42 to 1.04); P = <0.001
	Proportion mediated[1]	0.32	7.51
Safety Beliefs	Total effect	2.07 (-8.60 to 12.74); P = 0.703	3.59 (-7.00 to 14.17); P = 0.507
	Direct effect	2.68 (-7.52 to 12.89); P = 0.606	3.06 (-7.12 to 13.24); P = 0.556
	Indirect effect	-0.46 (-2.62 to 1.71); P = 0.680	1.16 (-1.06 to 3.37); P = 0.306
	a path	2.32 (-8.66 to 13.30); P = 0.679	-5.89 (-16.83 to 5.05); P = 0.291
	b path	-0.20 (-0.29 to -0.11); P = <0.001	-0.20 (-0.29 to -0.11); P = <0.001
	Proportion mediated[1]	0.22	0.32

[1] Indirect/total effect

3.7 Sensitivity analyses

As there were no outliers, there was no analysis carried out omitting outliers. Table 7 shows that there is still no significant treatment difference after adjusting for baseline measurements predictive of missingness (Ethnic group, number of mental health admissions, Anxiolytic use & EQ-5D-5L Index); there is no evidence of a treatment difference when considering only participants who completed their 4-week measures before the beginning of lockdown; nor when considering only those who completed their 24-week follow-up before lockdown. There is also no evidence of any medication prescription effect on the treatment groups; nor any difference between the groups for the credibility and expectancy scores. Regression diagnostic plots are not presented here but are available.

TABLE 8: SUMMARY STATISTICS AND THE TREATMENT DIFFERENCE BETWEEN THE RANDOMISED GROUPS FOR THE SENSITIVITY ANALYSES

Persecutory belief conviction	VRCB (N=39) mean (SD) [n]	VRMR (N=41) mean (SD) [n]	Adjusted mean difference [95% CI][1]	P value
**Adjusting for baseline characteristics associated with missingness [2]**				
4 weeks	52.1 (23.8) [38]	56.6 (25.0) [39]	-5.34 [-16.22 to 5.53]	0.336
24 weeks	51.9 (27.4) [35]	53.0 (29.0) [38]	-5.91 [-17.04 to 5.22]	0.298
**Impact of the COVID-19 pandemic - 4-week measures completed before the beginning of lockdown[3]**				
4 weeks	54.7 (22.8) [29]	57.0 (25.6) [26]	1.30 [-11.60 to 14.21]	0.843
24 weeks	45.6 (30.3) [17]	50.5 (30.7) [19]	-1.27 [-16.03 to 13.48]	0.866
**Impact of the COVID-19 pandemic - 24-week measures completed before the beginning of lockdown**				
4 weeks	51.1 (23.0) [18]	57.5 (26.2) [18]	-2.13 [-18.37 to 14.12]	0.797
24 weeks	45.6 (30.3) [17]	50.5 (30.7) [19]	-3.42 [-19.67 to 12.82]	0.680
**Medication effects - Prescription of antipsychotic medication expressed as a chlorpromazine equivalent score†**				
4 weeks	52.1 (23.8) [38]	56.6 (25.0) [39]	6.94 [-12.40 to 26.28]	0.482
24 weeks	51.9 (27.4) [35]	53.0 (29.0) [38]	-1.90 [-22.50 to 18.70]	0.857
**Credibility effects**				
Credibility score	18.8 (4.6) [39]	17.3 (4.7) [37]	1.55 [-0.58 to 3.68]	0.152
Expectancy score	18.8 (4.6) [39]	17.3 (4.7) [37]	0.91 [-1.61 to 3.42]	0.474

[1] VRCB versus VRMR: estimated from a linear mixed-effects model adjusting for baseline values of the outcome as fixed effects and participant as the random effect. Credibility effects analyses estimates obtained from a linear regression model with no adjustment for any other factors.
[2] Models further adjust for ethnicity, total pre-trial mental health admissions, baseline Anxiolytic use and baseline EQ5D Index Value
[3] Analysis on all participants who completed their 4-week measures before the beginning of lockdown on 16 March 2020 but setting any follow-up measures that were completed after lockdown as missing.
† Linear mixed-effects model includes a three-way interaction between randomised group, CPZ score and time
Level of significance = 0.05

3.8 Subgroup analyses

None carried out

3.9 Safety analyses

Frequencies of adverse and serious adverse events for each treatment VRCBre reported below.

All randomised participants were included in the safety analysis.

5 participants had more than one AE/SAE.

TABLE 9: SUMMARY OF SAFETY EVENTS BY RANDOMISED GROUP

	VRCB (N=39)	VRMR (N=41)
**Total number of safety events experienced**		
**AEs**		
0	37 (94.9)	39 (95.1)
1	1 (2.6)	1 (2.4)
2	-	1 (2.4)
3	1 (2.6)	-
**SAEs**		
0	32 (82.1)	36 (87.8)
1	5 (12.8)	4 (9.8)
2	1 (2.6)	-
3	1 (2.6)	1 (2.4)
**Number of participants experiencing at least one event**		
**AE**	2 (5.1)	2 (4.9)
**SAE**	7 (17.9)	5 (12.2)

Table 9 below gives a summary of the SAEs per participant.

TABLE 10: SUMMARY OF SAES PER PARTICIPANT

Subject ID	Treatment Group	Date of last session	SAE Start Date	Related to the therapy?
THRIVE07	VRMR	31jan2019	28may2019	No
THRIVE08	VRCB	18feb2019	01jul2019	No
THRIVE12	VRMR	11apr2019	01jul2019	No
THRIVE14	VRCB	04mar2019	09mar2019	No
THRIVE17	VRCB	26mar2019	09aug2019	No
THRIVE19	VRMR	26mar2019	20jun2019	No
THRIVE19	VRMR	26mar2019	01aug2019	No
THRIVE19	VRMR	26mar2019	01may2019	No
THRIVE29	VRCB	19jul2019	01oct2019	No
THRIVE29	VRCB	19jul2019	01nov2019	No
THRIVE29	VRCB	19jul2019	01dec2019	No
THRIVE33	VRCB	13aug2019	01nov2019	No
THRIVE33	VRCB	13aug2019	04sep2019	No
THRIVE38	VRMR	07oct2019	06dec2019	No
THRIVE58	VRCB	19mar2020	03apr2020	No
THRIVE67	VRMR	30mar2021	25may2021	Probably not
THRIVE76	VRCB	18jun2021	26jul2021	No

4 Additional exploratory analysis not specified in the SAP

4.1 Post-hoc investigation of Credibility and Expectancy rating

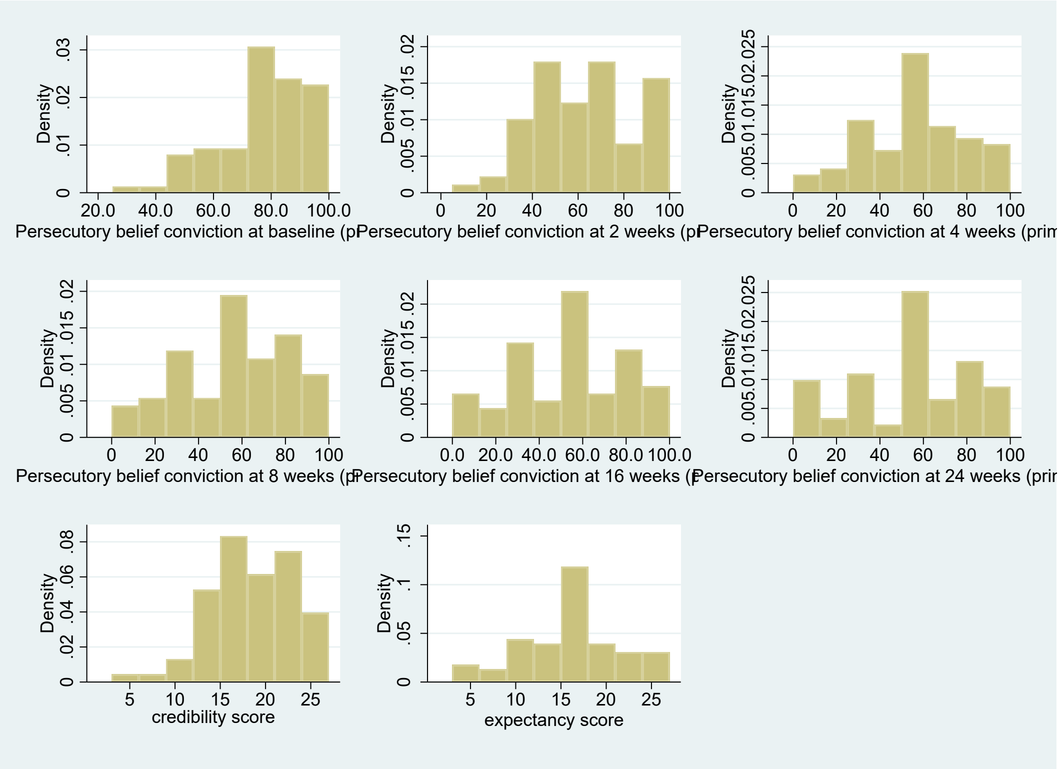

FIGURE 3: HISTOGRAMS FOR CONVICTION IN THE PERSECUTORY DELUSION (ALL TIME POINTS) AND CREDIBILITY & EXPECTANCY SCORES

The histograms above show that several of the outcomes are not normally distributed; thus Spearman correlation tests are performed to test for associations between persecutory delusions (each time point) and credibility & expectancy scores, respectively. Results are show in Table 10. Figure 2 and Figure 3 present the scatterplots of this data and show that there is no obvious trend in any of the plots. There are also some outliers visible in several of the plots. Spearman correlation is not as sensitive to these as the Pearson's correlation test.

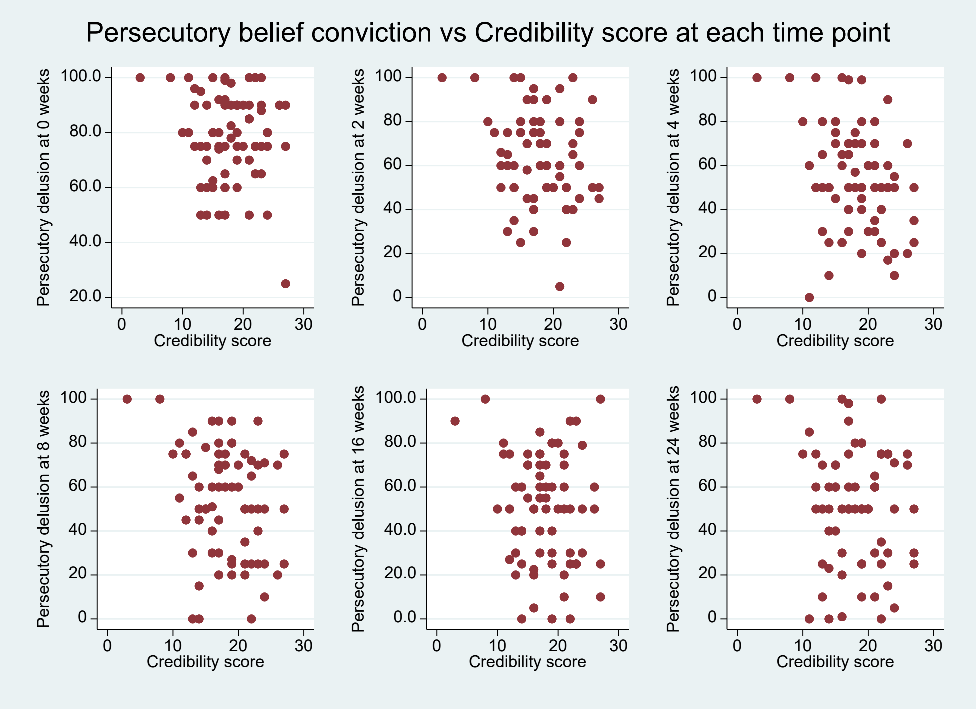

FIGURE 4: SCATTERPLOTS OF PERSECUTORY DELUSION (ALL TIME POINTS) VS. CREDIBILITY SCORE

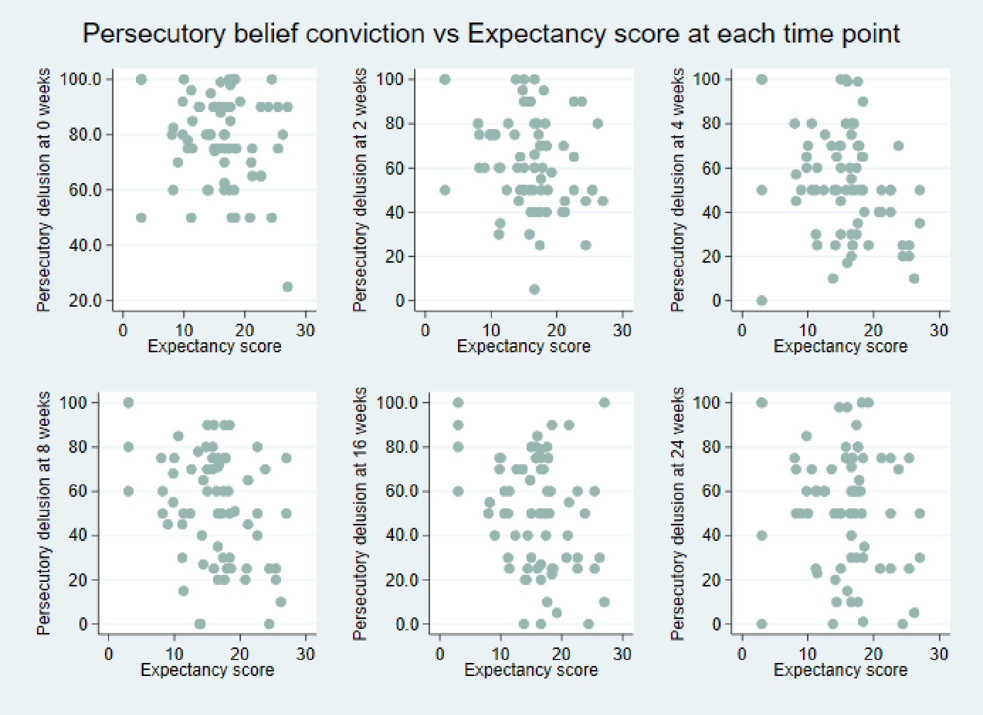

FIGURE 5: SCATTERPLOTS OF PERSECUTORY DELUSION (ALL TIME POINTS) VS. EXPECTANCY SCORE

TABLE 11: SPEARMAN CORRELATION TEST FOR CREDIBILITY AND EXPECTANCY VS. PERSECUTORY DELUSION (ALL TIME POINTS)

Persecutory delusion at week:	Credibility score rho, P value [n]	Expectancy score rho, P value [n]
0	-0.02; 0.840 [76]	-0.14; 0.228 [76]
2	-0.21; 0.078 [73]	-0.25; 0.034 [73]
4	-0.28; 0.015 [74]	-0.28; 0.015 [74]
8	-0.22; 0.061 [71]	-0.29; 0.014 [71]
16	-0.12; 0.326 [71]	-0.23; 0.049 [71]
24	-0.09; 0.435 [70]	-0.09; 0.441 [70]

Credibility score: there is evidence of an association with persecutory delusion at 4 weeks (rho=-0.28; P value=0.015). The negative correlation coefficient indicates an inverse association, i.e. as persecutory delusion increases, credibility decreases. However, the correlation is closer to zero than one and therefore indicates a weak association.

Expectancy score: There is evidence of an association with persecutory delusion at 2, 4, 8 and possibly 16 weeks. At all these time points the coefficient is closer to zero and negative, indicating weak negative correlations.

4.2 Post-hoc analysis on mediators

Exploring the effect of change in safety behaviours and safety beliefs on persecutory belief conviction:

	Safety Behaviours	Safety beliefs
	**Estimate (95% CI); P value [n]**	**Estimate (95% CI); P value [n]**
**Change in Mediators[1]**		
**Change from Baseline to 2-weeks (mid-treatment)**	-4.09 (-6.02 to -2.16); 0.0001 [70]	9.70 (3.15 to -16.24); 0.0042 [74]
**Change from Baseline to 4-weeks (end of treatment)**	-6.17 (-8.34 to -4.01); <0.0001 [70]	17.96 (10.94 to 24.98); <0.0001 [75]
**Effect of change in mediators on the Primary outcome (delusional conviction)[2]**		
**Change from Baseline to 2-weeks (mid-treatment)**	0.07 (-0.48 to 0.62); 0.809 [78]	0.05 (-0.17 to 0.29); 0.637 [80]
**Change from Baseline to 4-weeks (end of treatment)**	-0.05 (-0.57 to 0.48); 0.865 [78]	-0.06 (-0.29 to 0.17); 0.603 [80]
**Change from Baseline to 8-weeks**	0.54 (-0.01 to 1.09); 0.056 [78]	-0.10 (-0.32 to 0.12); 0.363 [80]
**Change from Baseline to 16-weeks**	**0.89 (0.25 to 1.52); 0.006 [78]**	**-0.33 (-0.56 to -0.12); 0.004 [80]**
**Change from Baseline to 24-weeks**	**0.61 (0.03 to 1.18); 0.038 [78]**	-0.15 (-0.36 to 0.06); 0.166 [80]

[1] Based on a paired t-test

[2] Mean difference estimated from a linear mixed-effects model of Persecutory belief conviction (primary outcome) adjusting for the mediator, time and an interaction between the mediator and time as fixed effects and participant as the random effect

The post-hoc analyses on Safety behaviours and Safety beliefs show that each of the mediators change significantly from baseline to 2- and 4-weeks for the whole group combined. However, these changes in the mediators are not associated with changes in the primary outcome from baseline to 2- and 4-weeks respectively (as shown by the regression results in the latter half of the table). For Safety behaviours, changes from baseline to 16 weeks and from baseline to 24 weeks are associated with positive (though small) changes in the primary outcome. For Safety beliefs, the only statistically significant association with primary outcome is at 16 weeks. For this time point, we can say that for every unit increase in Safety beliefs, delusion conviction decreases by 0.33 units on average.

Exploring the effect of change in persecutory belief conviction on safety behaviours and safety beliefs:

	Safety Behaviours	Safety beliefs
	**Estimate (95% CI); P value [n]**	**Estimate (95% CI); P value [n]**
**Change in Persecutory belief conviction**		
**Change from Baseline to 2-weeks (mid-treatment)**	-0.01 (-0.12 to 0.11); 0.903 [78]	**0.40 (0.05 to 0.74); 0.023 [80]**
**Change from Baseline to 4-weeks (end of treatment)**	-0.01 (-0.12 to 0.10); 0.889 [78]	**0.33 (0.002 to 0.66); 0.049 [80]**
**Change from Baseline to 8-weeks**	0.04 (-0.07 to 0.14); 0.511 [78]	0.23 (-0.09 to 0.55); 0.161 [80]
**Change from Baseline to 16-weeks**	0.03 (-0.08 to 0.14); 0.571 [78]	-0.05 (-0.38 to 0.28); 0.765 [80]
**Change from Baseline to 24-weeks**	0.06 (-0.04 to 0.17); 0.244 [78]	0.15 (-0.17 to 0.46); 0.356 [80]

[2] Mean difference in safety behaviours and safety beliefs estimated from a linear mixed-effects model on these outcomes, adjusting for persecutory belief conviction, time and an interaction between persecutory belief conviction and time, as fixed effects and participant as a random effect

The first column of the table above shows that there is no statistically significant change in safety behaviours with changes in persecutory belief conviction over time. The second column shows that a one point increase in persecutory belief conviction from baseline to 2 weeks leads to an average increase in safety beliefs of 0.4 points and a one point increase in persecutory belief conviction from baseline to 4 weeks leads to an average increase in safety beliefs of 0.33 points. There is no statistically significant effect of change in persecutory belief conviction on safety beliefs at any other time point.

5 References

None

6 Appendices

6.1 Appendix I. Diagnostic plots

Post estimate plots of the model residuals from the linear mixed effects models for the primary and secondary analyses are shown below together with histograms depicting the distribution of the (raw) outcome data. With the exception of the model for the C-SSRS, all the other model diagnostics indicate the model assumptions are satisfied.

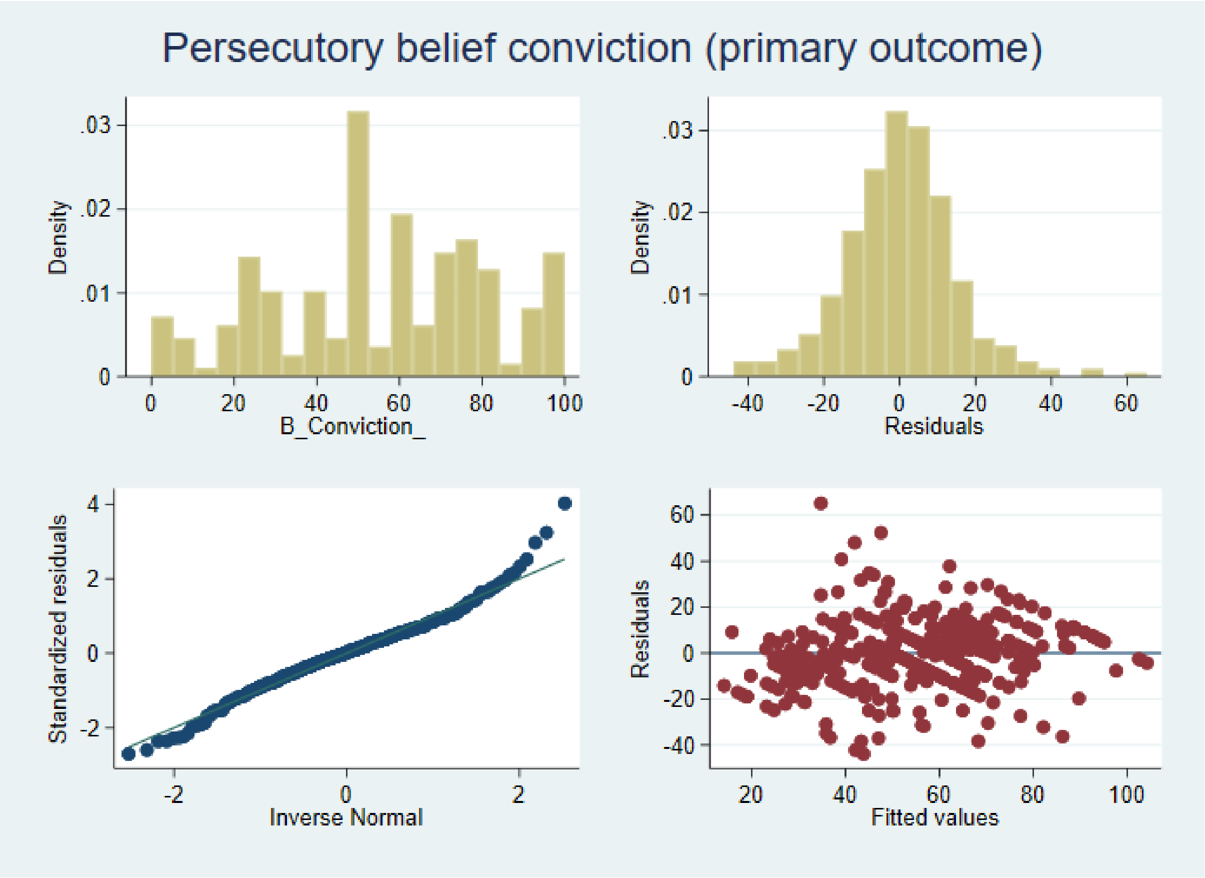

FIGURE 6: HISTOGRAMS AND MODEL RESIDUAL PLOTS FOR CONVICTION IN THE PERSECUTORY DELUSION (PRIMARY OUTCOME)

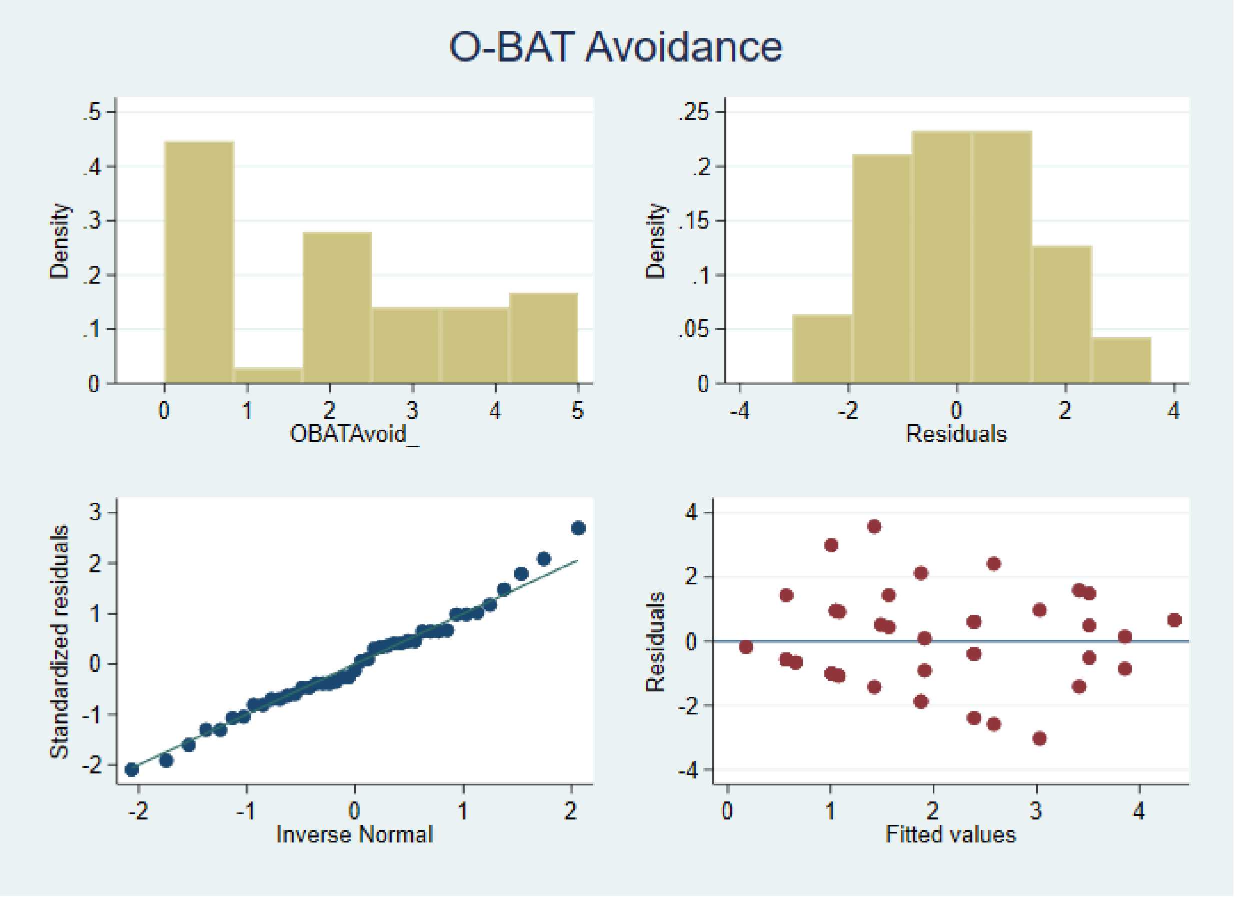

FIGURE 7: HISTOGRAMS AND MODEL RESIDUAL PLOTS FOR O-BAT - MAXIMUM NUMBER OF STEPS AVOIDED

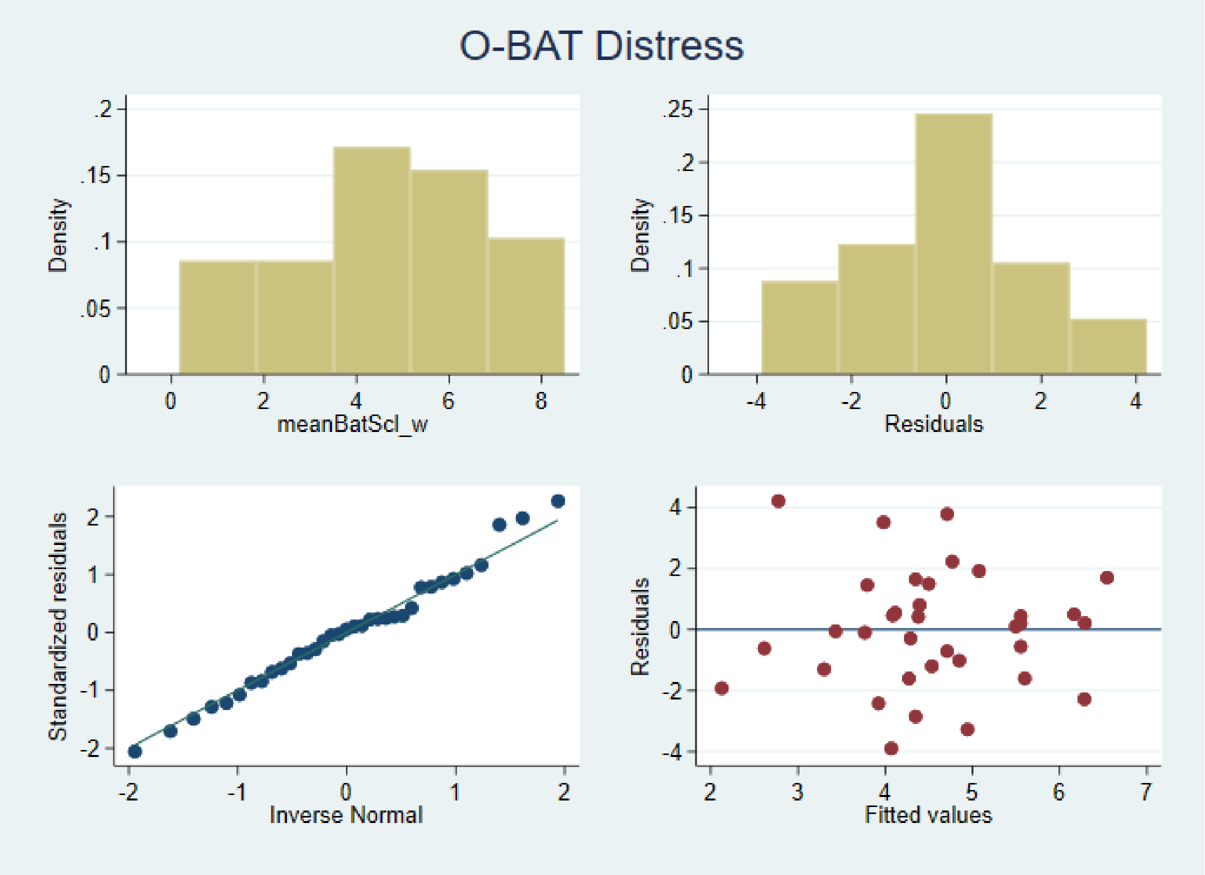

FIGURE 8: HISTOGRAMS AND MODEL RESIDUAL PLOTS FOR O-BAT - MEAN DISTRESS SCORE

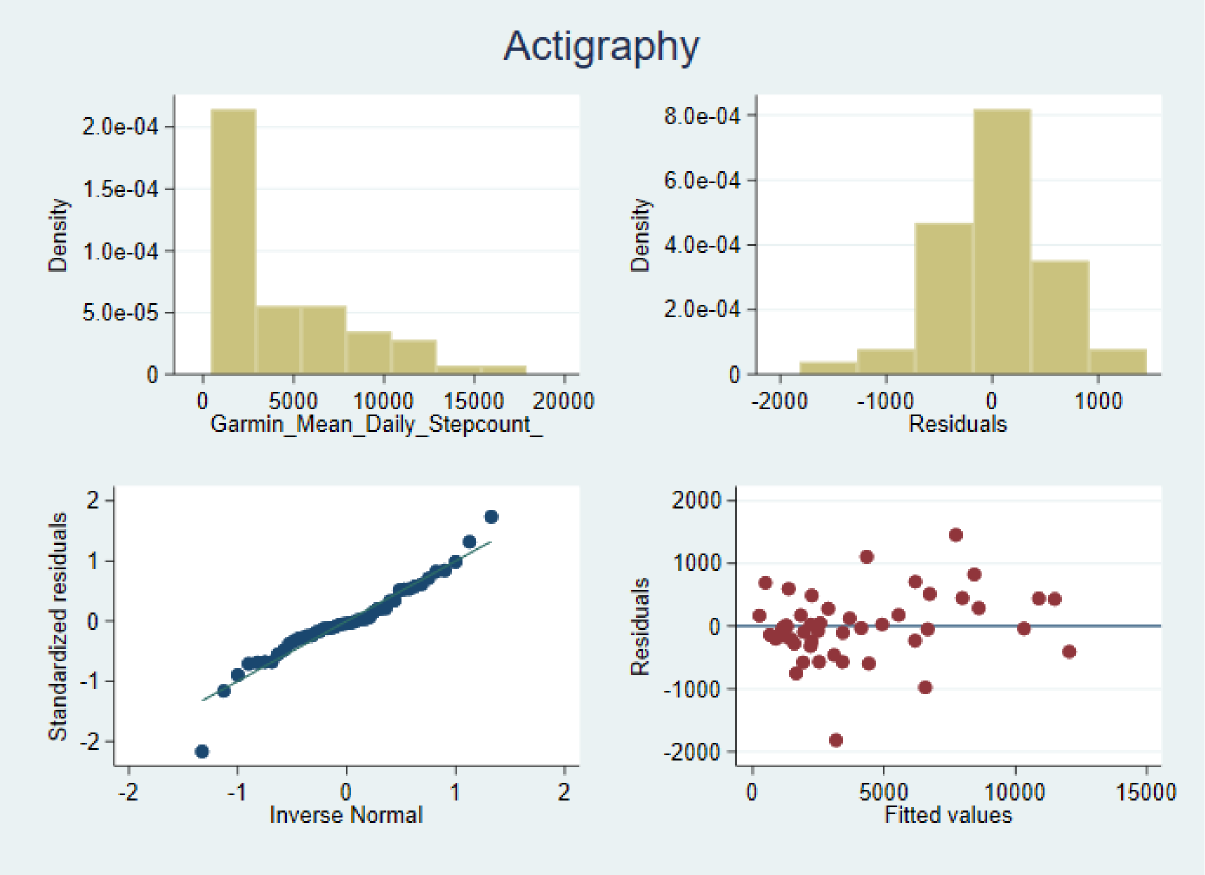

FIGURE 9: HISTOGRAMS AND MODEL RESIDUAL PLOTS FOR (ACTIGRAPHY) MEAN NUMBER OF STEPS (DAILY)

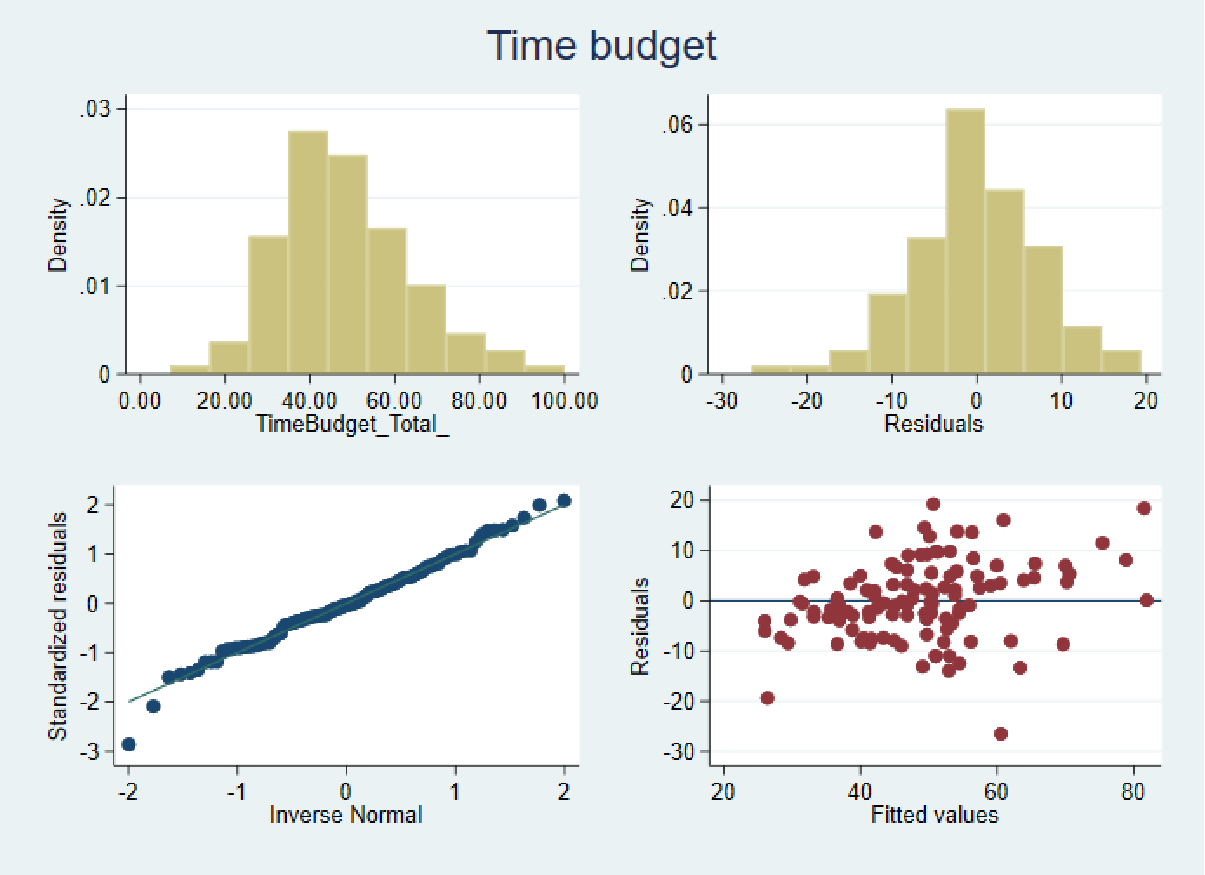

FIGURE 10: HISTOGRAMS AND MODEL RESIDUAL PLOTS FOR THE TIME BUDGET SCORE

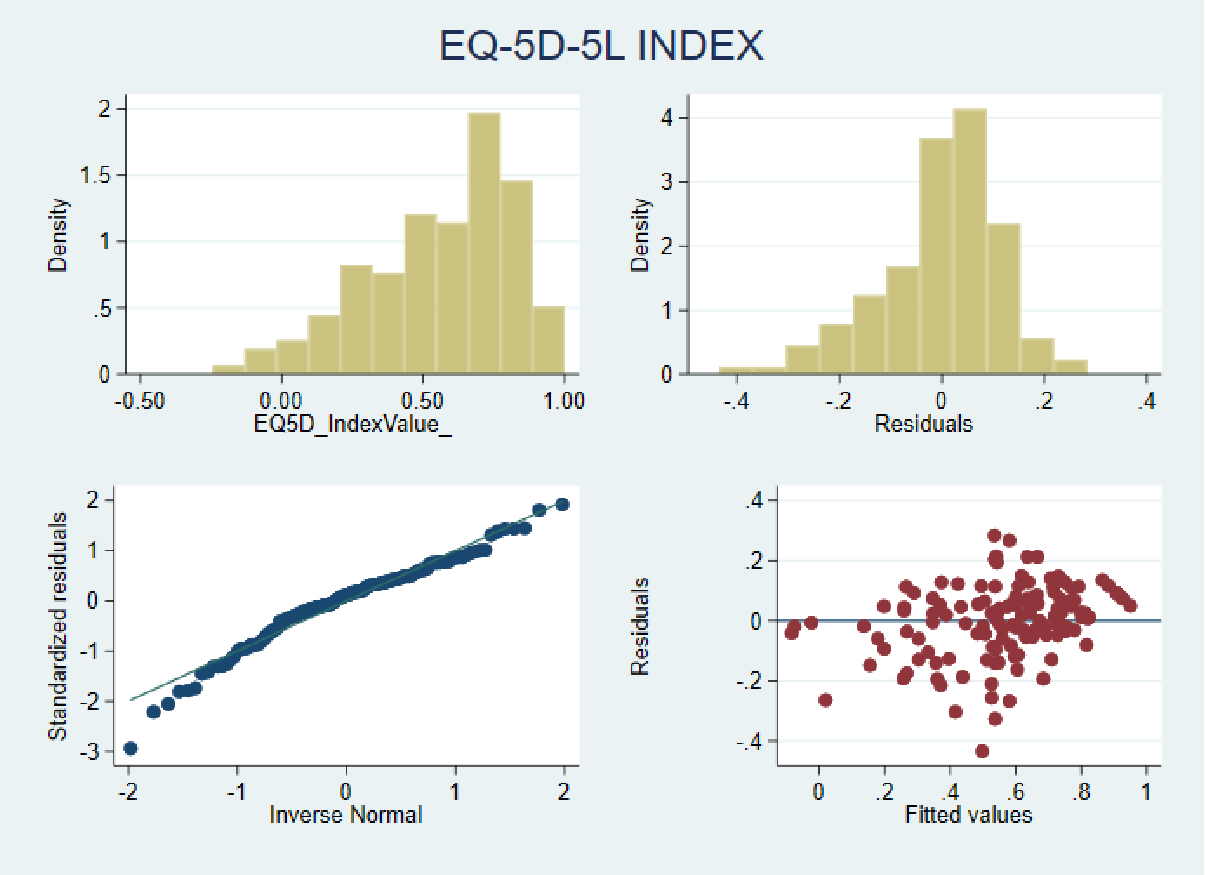

FIGURE 11: HISTOGRAMS AND MODEL RESIDUAL PLOTS FOR THE EQ-5D-5L INDEX

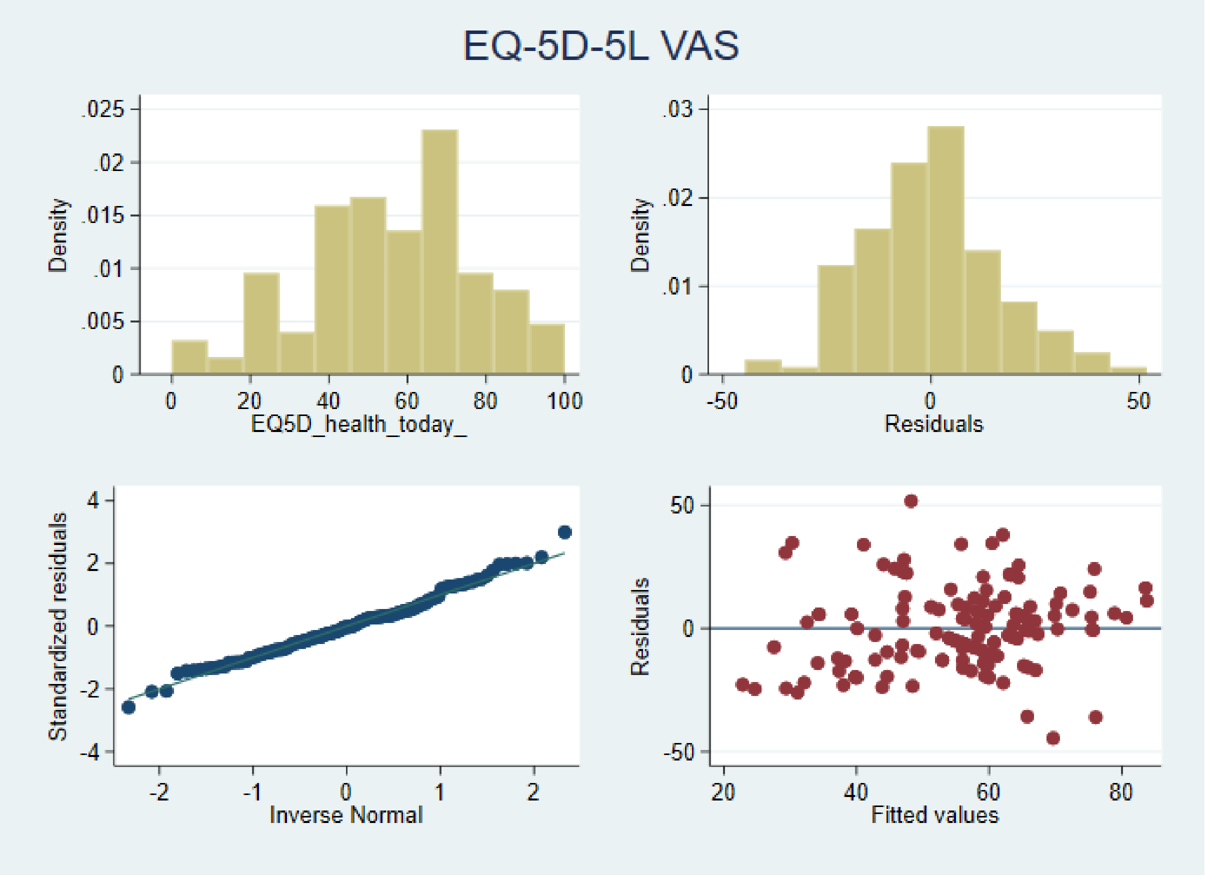

FIGURE 12: HISTOGRAMS AND MODEL RESIDUAL PLOTS FOR THE EQ5D VAS SCORE

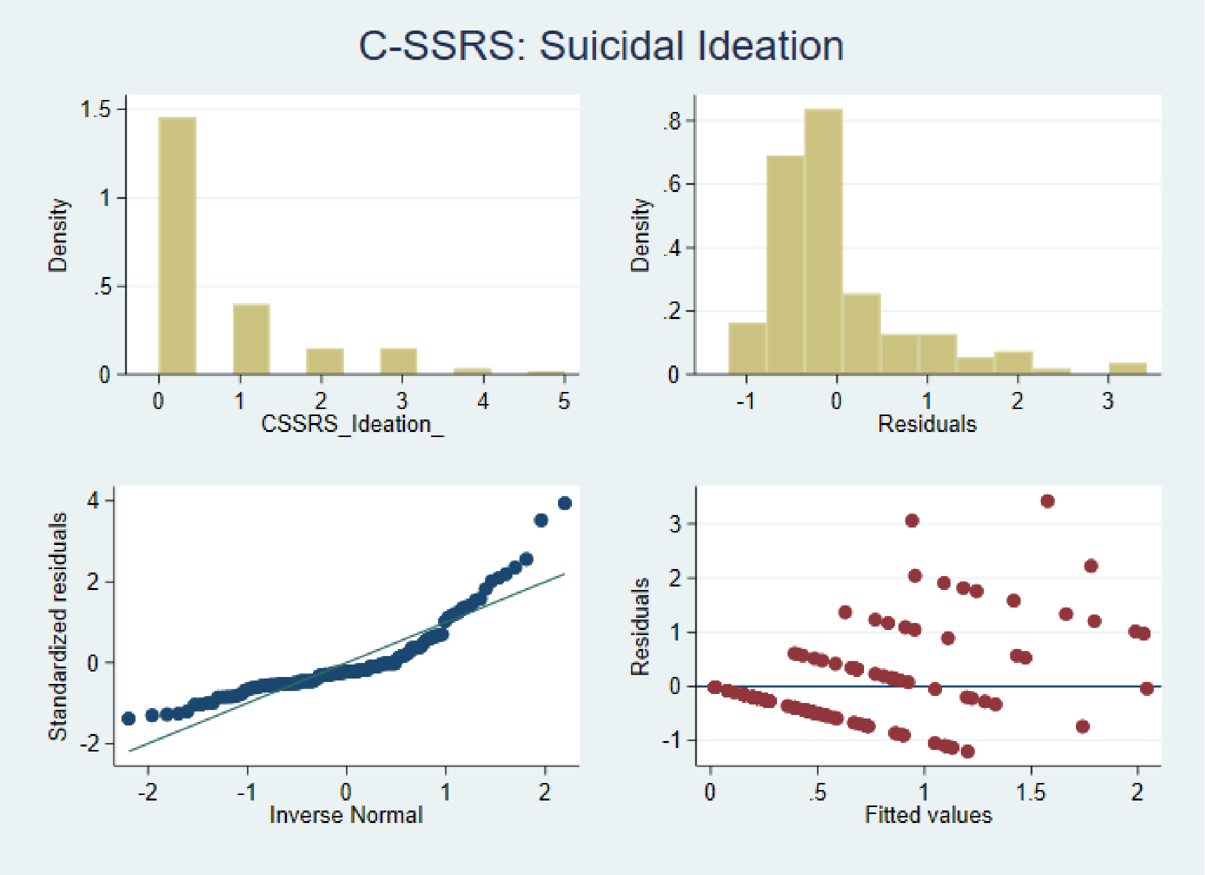

FIGURE 13: HISTOGRAMS AND MODEL RESIDUAL PLOTS FOR THE COLUMBIA SUICIDE SEVERITY RATING SCALE (C-SSRS) TOTAL SCORE

The histogram of the residuals shows that the assumption of normally distributed errors is violated. This model was fitted with continuous baseline but it did not improve the model fit. The outcome was also log transformed and the model fitted on the transformed variables, however this did not improve the results either. Therefore, the results should be reported with caution.

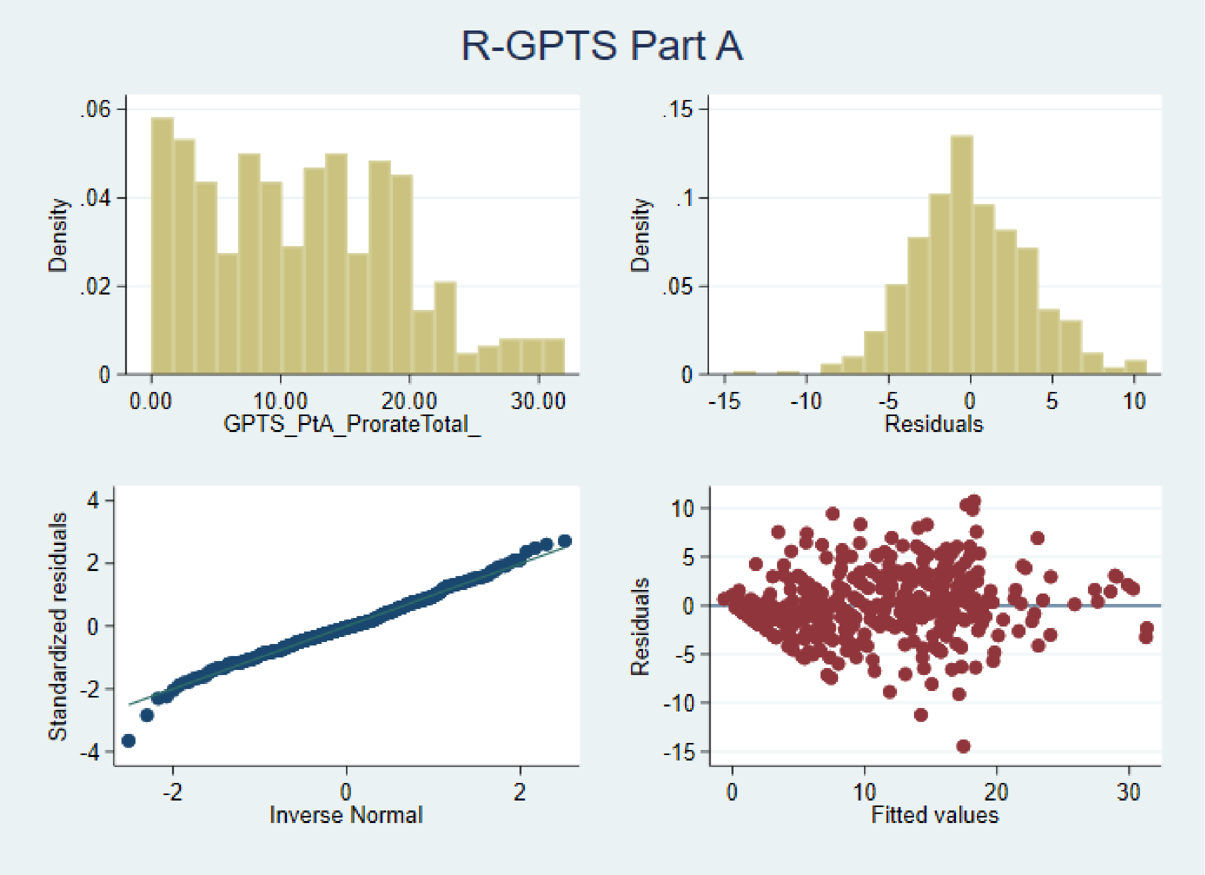

FIGURE 14: HISTOGRAMS AND MODEL RESIDUAL PLOTS FOR THE R-GPTS-A (SOCIAL REFERENCE) SCORE

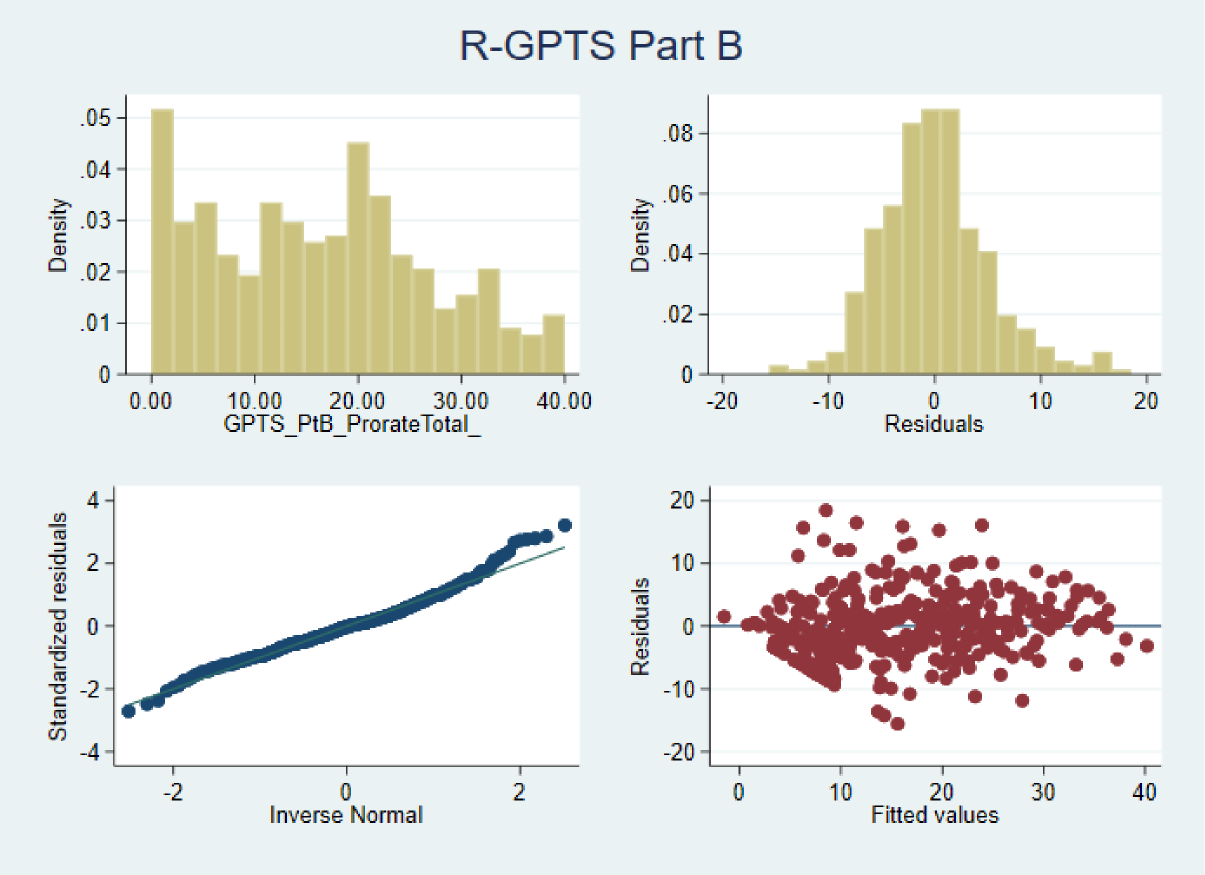

FIGURE 15: HISTOGRAMS AND MODEL RESIDUAL PLOTS FOR THE R-GPTS-B (PERSECUTION) SCORE

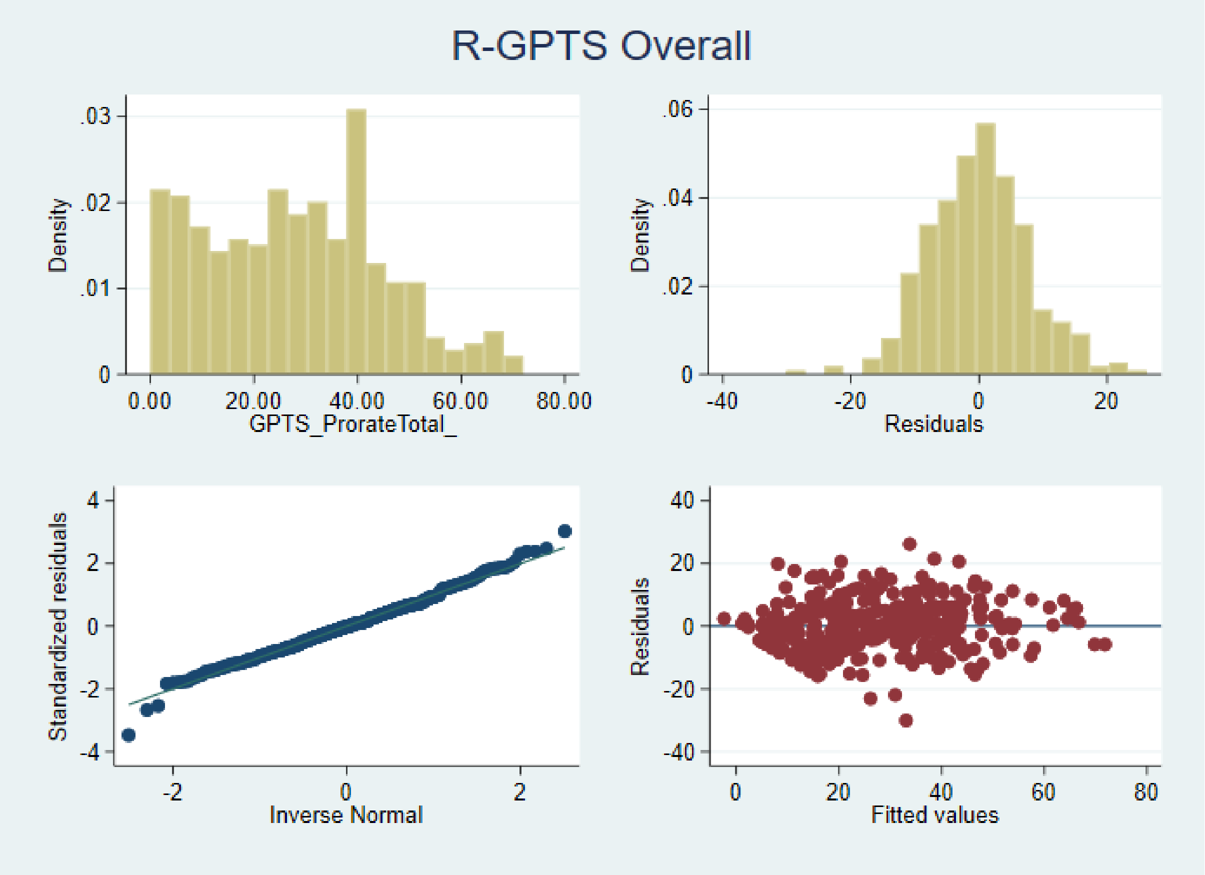

FIGURE 16: HISTOGRAMS AND MODEL RESIDUAL PLOTS FOR THE R-GPTS (OVERALL) SCORE

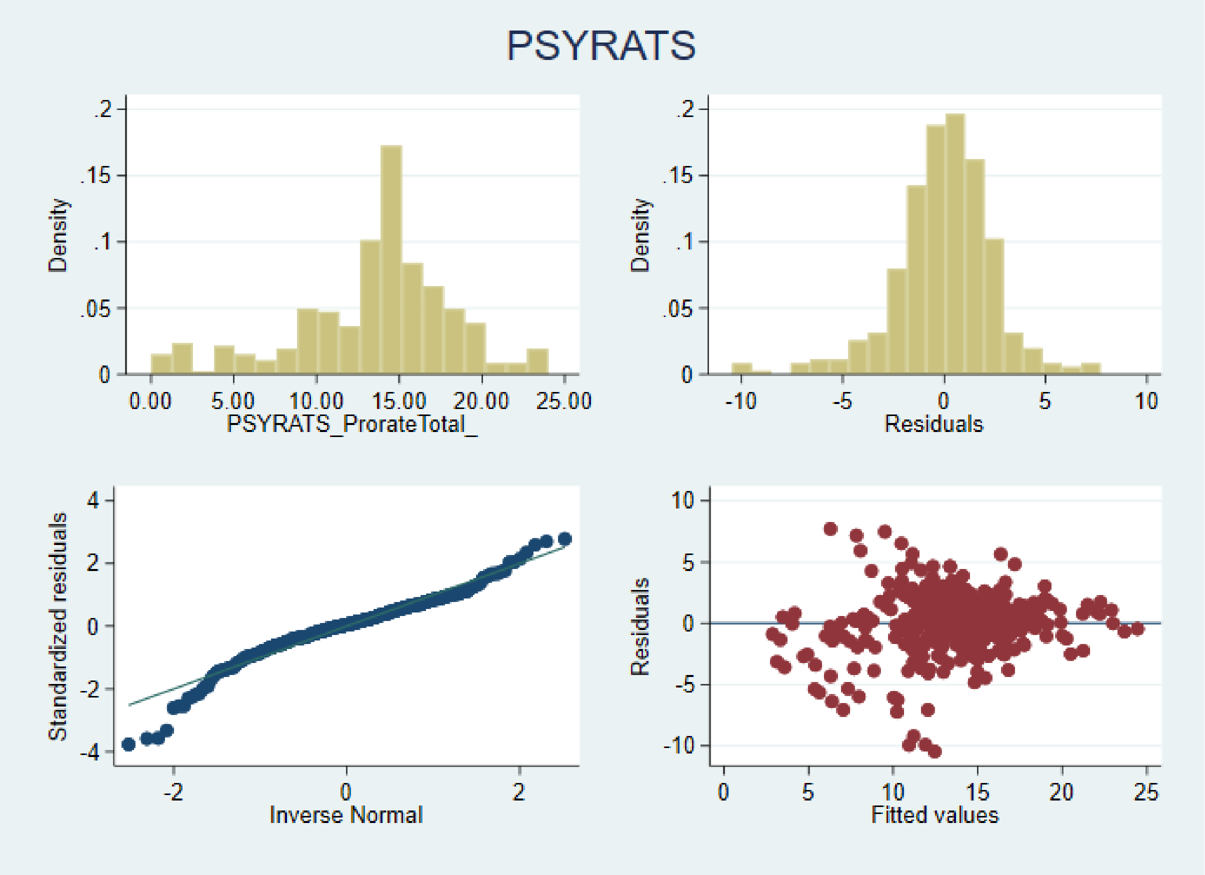

FIGURE 17: HISTOGRAMS AND MODEL RESIDUAL PLOTS FOR THE DELUSION SEVERITY (PSYRATS) SCORE

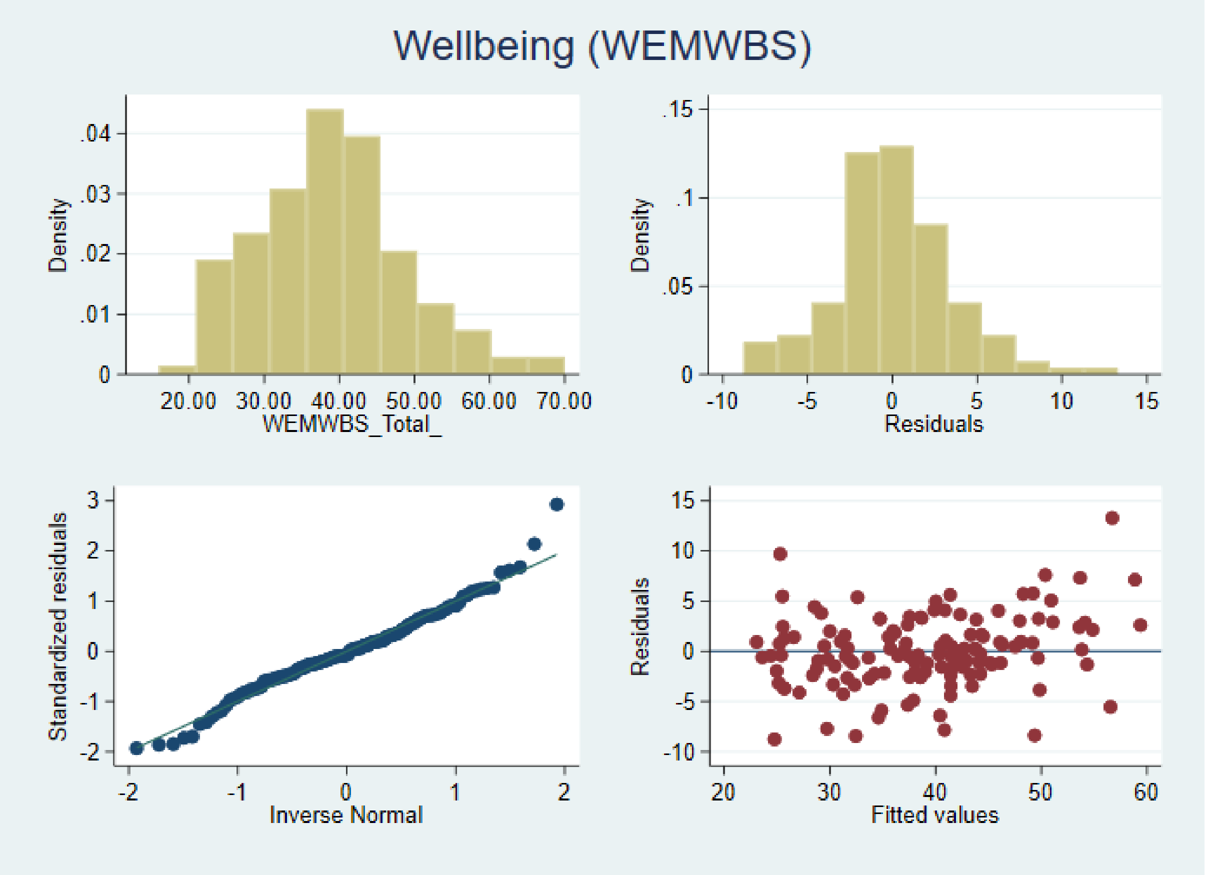

FIGURE 18: HISTOGRAMS AND MODEL RESIDUAL PLOTS FOR THE WELLBEING (WEMWBS) SCORE

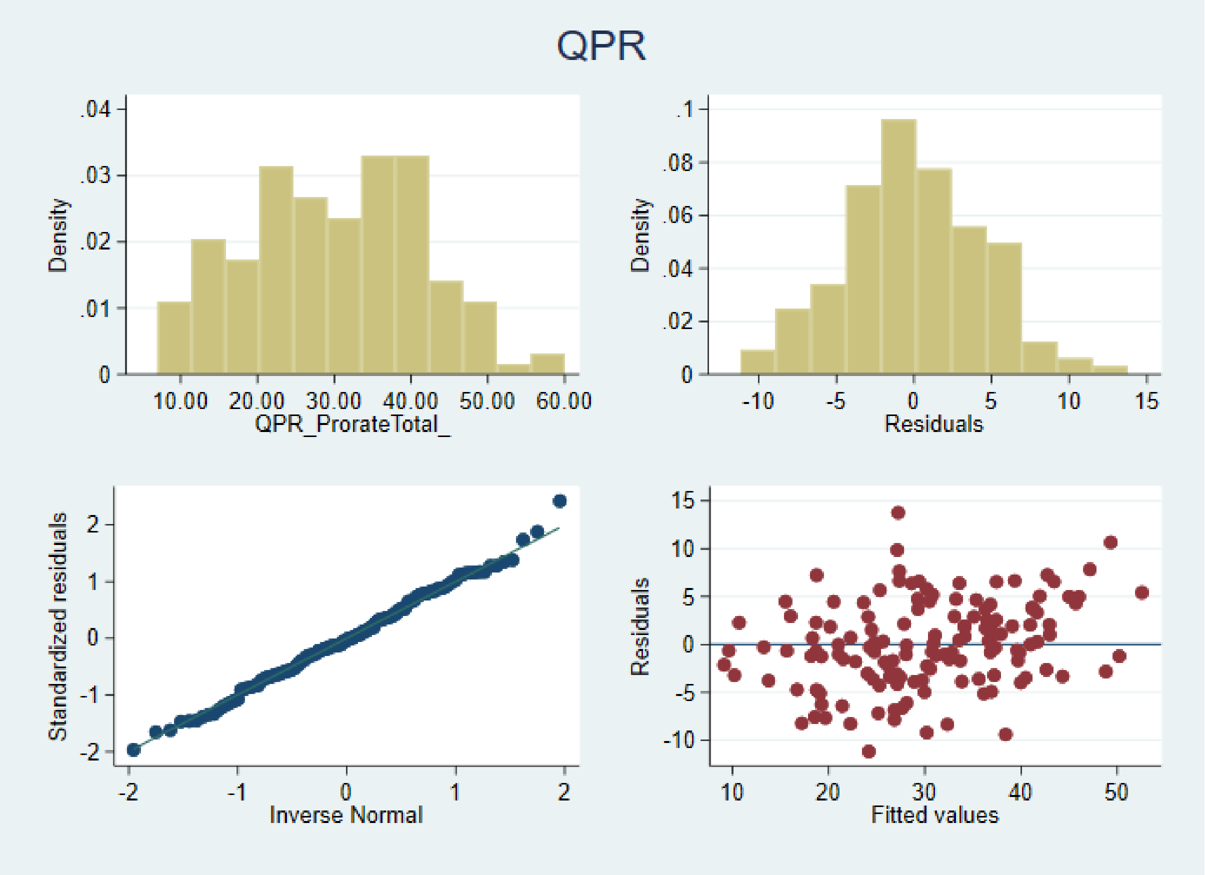

FIGURE 19: HISTOGRAMS AND MODEL RESIDUAL PLOTS FOR THE PROCESS OF RECOVERY (QPR) TOTAL SCORE

6.2 Appendix II. O-BAT Summary Tables

TABLE 12: SUMMARY OF STEPS COMPLETED FOR THE REAL WORLD DISTRESS OUTCOME (O-BAT), BY RANDOMISED GROUP

	VRCB (N=39)		VRMR (N=41)		Total (N=80)	
	**Baseline**	**4 weeks**	**Baseline**	**4 weeks**	**Baseline**	**4 weeks**
**Completed level 1**	28 (71.8)	18 (46.2)	24 (58.5)	19 (46.3)	52 (65.0)	37 (46.3)
**Distress level**						
Mean (SD)	4.3 (2.3)	3.9 (2.8)	4.6 (2.4)	3.9 (2.2)	4.5 (2.3)	3.9 (2.4)
Median (IQR)	4.0 (3.0 to 6.0)	4.5 (1.0 to 6.0)	4.2 (2.5 to 6.0)	4.0 (2.0 to 5.0)	4.0 (3.0 to 6.0)	4.0 (2.0 to 6.0)
Range	[0.0 to 8.9]	[0.0 to 8.0]	[1.0 to 9.0]	[0.0 to 8.0]	[0.0 to 9.0]	[0.0 to 8.0]
**Completed level 2**	24 (61.5)	15 (38.5)	19 (46.3)	17 (41.5)	43 (53.8)	32 (40.0)
**Distress level**						
Mean (SD)	5.6 (2.5)	4.7 (2.6)	5.8 (2.0)	4.2 (2.3)	5.7 (2.3)	4.4 (2.4)
Median (IQR)	5.8 (3.5 to 8.0)	5.1 (2.5 to 7.0)	6.0 (4.0 to 7.6)	4.0 (3.0 to 5.0)	6.0 (4.0 to 8.0)	4.5 (2.8 to 6.0)
Range	[0.0 to 9.0]	[0.0 to 8.0]	[2.0 to 10.0]	[1.0 to 9.0]	[0.0 to 10.0]	[0.0 to 9.0]
**Completed level 3**	12 (30.8)	12 (30.8)	15 (36.6)	15 (36.6)	27 (33.8)	27 (33.8)
**Distress level**						
Mean (SD)	4.6 (3.5)	4.4 (2.9)	6.4 (2.1)	4.8 (1.9)	5.6 (2.9)	4.6 (2.3)
Median (IQR)	6.0 (0.5 to 7.2)	5.0 (1.5 to 7.0)	7.0 (5.0 to 8.0)	5.0 (4.0 to 5.8)	7.0 (4.0 to 8.0)	5.0 (3.0 to 6.0)
Range	[0.0 to 9.0]	[0.0 to 8.0]	[2.0 to 9.2]	[2.0 to 9.6]	[0.0 to 9.2]	[0.0 to 9.6]
**Completed level 4**	6 (15.4)	7 (17.9)	8 (19.5)	10 (24.4)	14 (17.5)	17 (21.3)
**Distress level**						
Mean (SD)	5.3 (2.0)	3.6 (3.1)	7.1 (1.9)	4.8 (2.8)	6.3 (2.1)	4.3 (2.9)
Median (IQR)	5.8 (4.3 to 7.0)	2.0 (1.0 to 7.0)	7.5 (5.0 to 9.0)	5.0 (2.0 to 7.0)	6.8 (5.0 to 8.0)	4.0 (2.0 to 7.0)
Range	[2.0 to 7.0]	[0.0 to 8.0]	[5.0 to 9.0]	[1.0 to 9.0]	[2.0 to 9.0]	[0.0 to 9.0]
**Completed level 5**	4 (10.3)	6 (15.4)	5 (12.2)	10 (24.4)	9 (11.3)	16 (20.0)
**Distress level**						
Mean (SD)	6.6 (3.2)	4.2 (3.7)	7.3 (1.9)	4.9 (2.8)	7.0 (2.4)	4.6 (3.0)
Median (IQR)	8.1 (4.9 to 8.3)	4.0 (1.0 to 7.0)	7.0 (6.0 to 9.0)	5.0 (2.0 to 8.0)	8.0 (6.0 to 8.5)	5.0 (2.0 to 7.5)
Range	[1.8 to 8.5]	[0.0 to 9.0]	[5.0 to 9.5]	[1.0 to 8.5]	[1.8 to 9.5]	[0.0 to 9.0]
**Total Completed[1]**	74	58	71	71	145	129
**Distress level**						
Mean (SD)	5.0 (2.6)	4.2 (2.8)	5.8 (2.3)	4.4 (2.3)	5.4 (2.5)	4.3 (2.5)
Median (IQR)	5.0 (3.0 to 7.0)	5.0 (1.0 to 7.0)	6.0 (4.0 to 8.0)	4.0 (3.0 to 6.0)	5.5 (4.0 to 7.4)	4.0 (2.0 to 6.0)
Range	[0.0 to 9.0]	[0.0 to 9.0]	[1.0 to 10.0]	[0.0 to 9.6]	[0.0 to 10.0]	[0.0 to 9.6]

[1] Summary of all levels completed by each participant and overall level of distress

TABLE 13: SUMMARY OF HIGHEST STEP COMPLETED FOR THE REAL WORLD DISTRESS OUTCOME (O-BAT), BY RANDOMISED GROUP

Highest step completed	VRCB (N=39)		VRMR (N=41)		Total (N=80)	
	Baseline	4 weeks	Baseline	4 weeks	Baseline	4 weeks
**Completed level 1**	4 (10.3)	3 (7.7)	5 (12.2)	2 (4.9)	9 (11.3)	5 (6.3)
**Distress level**						
Mean (SD)	5.9 (1.4)	5.3 (2.9)	6.6 (2.8)	5.5 (2.1)	6.3 (2.2)	5.4 (2.3)
Median (IQR)	6.0 (5.0 to 6.7)	7.0 (2.0 to 7.0)	7.5 (6.0 to 8.5)	5.5 (4.0 to 7.0)	6.0 (6.0 to 7.5)	7.0 (4.0 to 7.0)
Range	[4.0 to 7.4]	[2.0 to 7.0]	[2.0 to 9.0]	[4.0 to 7.0]	[2.0 to 9.0]	[2.0 to 7.0]
**Completed level 2**	12 (30.8)	3 (7.7)	4 (9.8)	2 (4.9)	16 (20.0)	5 (6.3)
**Distress level**						
Mean (SD)	6.4 (2.3)	7.0 (1.7)	8.1 (1.9)	6.0 (4.2)	6.8 (2.3)	6.6 (2.5)
Median (IQR)	7.0 (5.0 to 8.0)	8.0 (5.0 to 8.0)	8.5 (6.8 to 9.5)	6.0 (3.0 to 9.0)	8.0 (5.3 to 8.4)	8.0 (5.0 to 8.0)
Range	[2.0 to 9.0]	[5.0 to 8.0]	[5.5 to 10.0]	[3.0 to 9.0]	[2.0 to 10.0]	[3.0 to 9.0]
**Completed level 3**	6 (15.4)	5 (12.8)	7 (17.1)	5 (12.2)	13 (16.3)	10 (12.5)
**Distress level**						
Mean (SD)	5.2 (4.1)	5.7 (2.7)	7.7 (1.0)	6.1 (2.0)	6.5 (3.0)	5.9 (2.3)
Median (IQR)	7.0 (0.0 to 8.0)	6.6 (6.0 to 7.0)	8.0 (7.0 to 8.0)	5.0 (5.0 to 6.0)	7.9 (7.0 to 8.0)	6.0 (5.0 to 7.0)
Range	[0.0 to 9.0]	[1.0 to 8.0]	[6.0 to 9.2]	[5.0 to 9.6]	[0.0 to 9.2]	[1.0 to 9.6]
**Completed level 4**	2 (5.1)	0	3 (7.3)		5 (6.3)	1 (1.3)
**Distress level**						
Mean (SD)	4.3 (3.2)	-	7.0 (2.0)		5.9 (2.6)	7.0 (.)
Median (IQR)	4.3 (2.0 to 6.5)	-	7.0 (5.0 to 9.0)		6.5 (5.0 to 7.0)	7.0 (7.0 to 7.0)
Range	[2.0 to 6.5]	-	[5.0 to 9.0]		[2.0 to 9.0]	[7.0 to 7.0]
**Completed level 5**	4 (10.3)	6 (15.4)	5 (12.2)	10 (24.4)	9 (11.3)	16 (20.0)
**Distress level**						
Mean (SD)	6.6 (3.2)	4.2 (3.7)	7.3 (1.9)	4.9 (2.8)	7.0 (2.4)	4.6 (3.0)
Median (IQR)	8.1 (4.9 to 8.3)	4.0 (1.0 to 7.0)	7.0 (6.0 to 9.0)	5.0 (2.0 to 8.0)	8.0 (6.0 to 8.5)	5.0 (2.0 to 7.5)
Range	[1.8 to 8.5]	[0.0 to 9.0]	[5.0 to 9.5]	[1.0 to 8.5]	[1.8 to 9.5]	[0.0 to 9.0]
**Total Completed[1]**	28 (71.8)	18 (46.2)	24 (58.5)	19 (46.3)	52 (65.0)	37 (46.3)
**Distress level**						
Mean (SD)	5.9 (2.7)	5.4 (2.9)	7.4 (1.8)	5.4 (2.5)	6.6 (2.5)	5.4 (2.7)
Median (IQR)	6.8 (4.5 to 8.0)	6.8 (2.0 to 7.0)	8.0 (6.0 to 9.0)	5.0 (3.1 to 8.0)	7.2 (5.8 to 8.1)	6.0 (3.1 to 7.0)
Range	[0.0 to 9.0]	[0.0 to 9.0]	[2.0 to 10.0]	[1.0 to 9.6]	[0.0 to 10.0]	[0.0 to 9.6]

[1] Summary over all maximum levels completed by each participant and level of distress corresponding to these

6.3 Appendix III. Reviewer requested subgroup analysis on gender (Post-hoc analysis)

Below are the results of a subgroup analysis of Gender on the primary outcome of Persecutory belief conviction at 4 weeks. The P value reported is for the gender x randomised group interaction and shows that there is no evidence of a statistically significant interaction between the treatment groups and gender.

TABLE 14: SUBGROUP ANALYSIS FOR PRIMARY OUTCOME AT 4 WEEKS

Subgroup analysis on Sex	VRCB (N=39)	VRMR (N=41)	Interaction effect [95% CI][1]	Test of Interaction (P value)
**Male, mean (sd) [n]**	53.1 (23.0) [24]	59.2 (22.8) [23]	-4.52 [-18.24 to 9.20]	0.863
**Female, mean (sd) [n]**	50.4 (25.8) [14]	52.9 (28.2) [16]	-2.62 [-19.77 to 14.54]	

[1] VRCB versus VRMR: Linear regression model for the primary outcome modelled against treatment group, outcome score at baseline and an interaction between randomised group and Gender.
Level of significance = 0.05

Figure. Conviction in the persecutory delusion over time by allocation group.

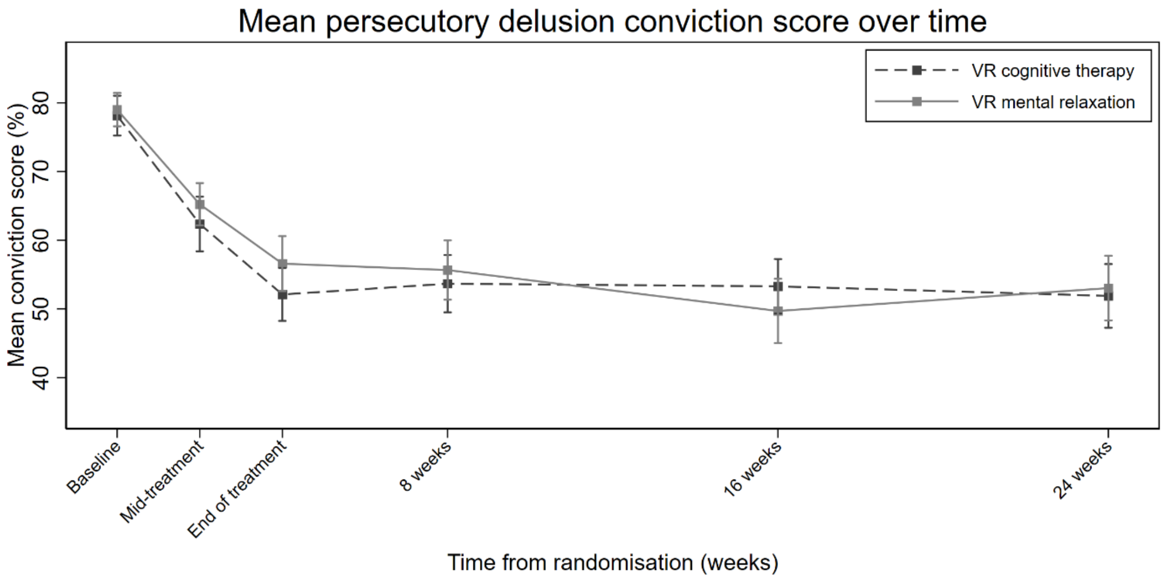

Note: Bars indicate standard errors of the mean
